# Microwave-assisted synthesis of 5,6-dihydroindolo[1,2-*a*]quinoxaline derivatives through copper-catalyzed intramolecular *N*-arylation

**DOI:** 10.3762/bjoc.9.285

**Published:** 2013-11-14

**Authors:** Fei Zhao, Lei Zhang, Hailong Liu, Shengbin Zhou, Hong Liu

**Affiliations:** 1 CAS Key Laboratory of Receptor Research, Shanghai Institute of Materia Medica, Chinese Academy of Sciences, 555 Zuchongzhi Road, Shanghai 201203, P. R. China

**Keywords:** copper, 5,6-dihydroindolo[1,2-*a*]quinoxaline, intramolecular N-arylation, microwave irradiation

## Abstract

An efficient and practical protocol has been developed to synthesize 5,6-dihydroindolo[1,2-*a*]quinoxaline derivatives by CuI-catalyzed intramolecular *N*-arylation under microwave irradiation. This method rapidly afforded the tetracyclic products with good to excellent yields (83–97%) in short reaction times (45–60 min).

## Introduction

**Figure 1:**
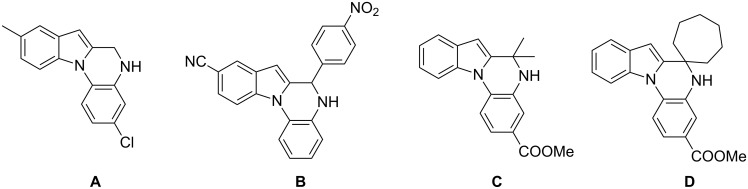
Representative biologically relevant examples of 5,6-dihydroindolo[1,2-*a*]quinoxaline derivatives.

The indole scaffold is considered as a privileged structure because of its ubiquitous presence in a large number of natural products and pharmaceutical agents [1–6]. In particular, indole-fused heterocycles have received much attention because of their applications in medicinal chemistry [7–11]. Among them, the tetracyclic ring system of 5,6-dihydroindolo[1,2-*a*]quinoxalines forms an important class of compounds because of their diverse range of pharmacological properties (Figure 1). For example, compound **A** shows pronounced antihistaminic activity [12]. Compound **B** is identified as a promising antifungal reagent against phytopathogenic fungi in vitro [13], and both compounds **C** and **D** exhibit a good inhibitory activity against VEGFR-3 kinase cells [14]. Therefore, an efficient access to this fused tetracyclic architecture is highly desirable for drug discovery.

Traditional copper-catalyzed Ullmann-type C–N coupling has been a powerful method to form the carbon–nitrogen bond [15–19]. However, the utility of the reaction is limited by the necessary high temperatures, the requirement of stoichiometric quantities of copper catalyst and low to moderate yields [20]. A recent breakthrough to overcome these drawbacks involves the use of appropriate ligands such as diamines and amino acids [21–24] that can enhance the activity of the copper catalysts and accelerate the reactions. As a result, the copper-catalyzed *N*-arylation has been extensively utilized for C–N coupling, especially for the arylation of N-containing heterocycles such as indoles, imidazoles, indazoles, pyrroles, pyrazoles and triazoles [25–28] to construct more fused heterocycles.

In recent years, several approaches toward the synthesis of 5,6-dihydroindolo[1,2-*a*]quinoxaline derivatives have been reported [29–42]: (a) Ru- and Au-catalyzed cascade reactions between 2-(1*H*-indol-1-yl)anilines and alkynes [34,42]. (b) AlCl_3_-catalyzed Pictet–Spengler reactions between 2-(1*H*-indol-1-yl)anilines and aromatic aldehydes [38]. (c) Pd-catalyzed regioselective C–H olefination/cyclization sequences from indole-substituted anilines and electron-deficient terminal alkenes [37]. However, these methods require expensive metal catalysts, long reaction time and produce only moderate yields. In this study, we tried to overcome these limitations by using copper-catalyzed C–N coupling and microwave-assisted organic synthesis, and we present an efficient and practical protocol, which rapidly synthesized 5,6-dihydroindolo[1,2-*a*]quinoxaline derivatives by copper-catalyzed intramolecular *N*-arylation with good to excellent yields.

## Results and Discussion

**Scheme 1:**
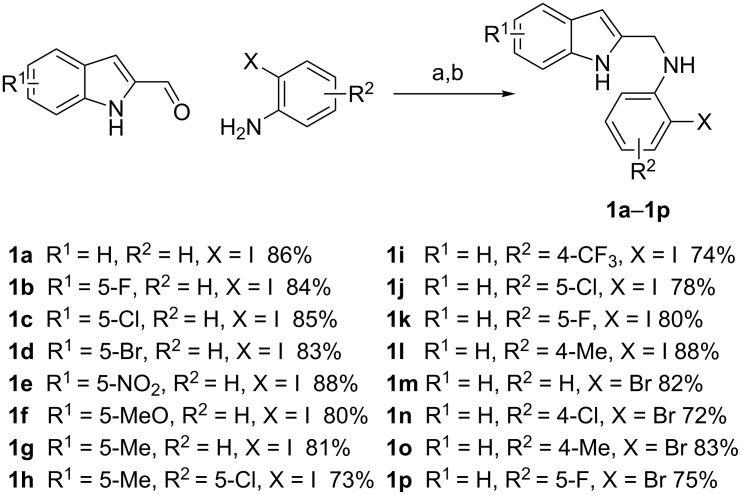
Reagents and conditions: (a) CF_3_COOH, anhydrous dichloromethane, reflux; (b) NaBH_4_, MeOH.

The required cyclization precursors **1**, aryl substituted (1*H*-indol-2-yl)methanamines, were easily prepared by reductive amination of 1*H*-indole-2-carbaldehydes with 2-haloanilines in good yields (Scheme 1).

Initial screening experiments were performed by employing **1a** as the model substrate in order to optimize the intramolecular cyclization conditions for ligands, bases, solvents, temperature and time. As shown in Table 1, the reaction was initially carried out with CuI as the catalyst, **L1** as the ligand, and K_3_PO_4_ as the base in toluene at 110 °C heated in an oil bath for 10 h. Unfortunately, most of the substrate **1a** was recovered (Table 1, entry 1) and the desired product was obtained only in low yield (38%). Considering that microwave-assisted organic synthesis (MAOS) is time- and energy-saving [43–45], we then chose this technology to conduct the intramolecular *N*-arylation. As a result, a similar yield was obtained under the same catalytic conditions when **1a** was subjected to microwave irradiation for just one hour (Table 1, entry 2). Then, we tried to optimize the reaction conditions under microwave heating. At first, various ligands were evaluated. Among them, **L6** was the most effective ligand for the *N*-arylation (Table 1, entries 3–7). Because of the deiodination of the reactant, product **2a** was obtained only in moderate yield (52%). Therefore, to eliminate the deiodination byproduct, we decreased the reaction temperature to 90 °C, and a slightly higher yield was obtained (Table 1, entry 8). With L-proline as the best ligand, a further screening of the solvents revealed that increasing the polarity of the solvent had a positive effect on the reaction yield, and DMSO displayed as the best choice to promote the transformation with 85% yield (Table 1, entries 9–11). Next, an examination of the bases revealed that the moderately strong base K_2_CO_3_ produced the best yield (92%), both a weaker base (K_3_PO_4_) and a stronger base (Cs_2_CO_3_) resulted in decreased yields (Table 1, entries 11–13). In addition, a study of the reaction time proved that 45 minutes was just enough to complete the transformation with an excellent yield (Table 1, entry 14), while a further reduction of the reaction time led to a decreased yield (Table 1, entry 15). In this way, the optimal reaction conditions were identified to be the catalytic system of CuI/L-proline/K_2_CO_3_ in DMSO under microwave irradiation for 45 minutes.

**Table 1:** Optimization of the reaction conditions for the Cu-catalyzed synthesis of 5,6-dihydroindolo[1,2-*a*]quinoxaline (**2a**).^a^

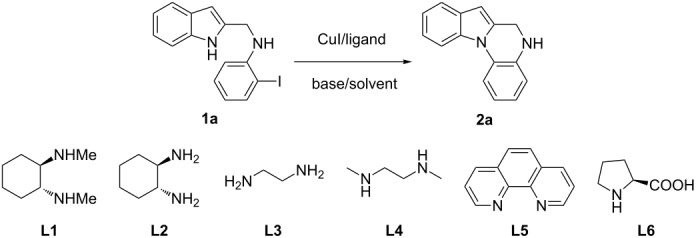				

entry	ligand	base	solvent/temperature/time	yield (%)^b^

1^c^	**L1**	K_3_PO_4_	toluene/110 °C/10 h	38^d^
2	**L1**	K_3_PO_4_	toluene/110 °C/1 h	36^d^
3	**L2**	K_3_PO_4_	toluene/110 °C/1 h	35^d^
4	**L3**	K_3_PO_4_	toluene/110 °C/1 h	10^d^
5	**L4**	K_3_PO_4_	toluene/110 °C/1 h	trace^d^
6	**L5**	K_3_PO_4_	toluene/110 °C/1 h	trace^d^
7	**L6**	K_3_PO_4_	toluene/110 °C/1 h	52
8	**L6**	K_3_PO_4_	toluene/90 °C/1 h	58
9	**L6**	K_3_PO_4_	1,4-dioxane/90 °C/1 h	64
10	**L6**	K_3_PO_4_	CH_3_CN/90 °C/1 h	72
11	**L6**	K_3_PO_4_	DMSO/90 °C/1 h	85
12	**L6**	K_2_CO_3_	DMSO/90 °C/1 h	92
13	**L6**	Cs_2_CO_3_	DMSO/90 °C/1 h	88
14	**L6**	K_2_CO_3_	DMSO/90 °C/45 min	92
15	**L6**	K_2_CO_3_	DMSO/90 °C/30 min	80

^a^Unless noted, reactions were performed with **1a** (0.25 mmol), CuI (0.025 mmol), ligand (0.05 mmol), and base (0.5 mmol) in solvent (2 mL) at the indicated temperature under microwave irradiation (sealed vessel at fixed power, 30 W). ^b^Isolated yield. ^c^Heated with oil bath. ^d^**1a** was recovered.

After determining the optimal reaction conditions, we then examined the general applicability of the process. First, the substituents of the indole moiety were explored (Table 2). Halogens (F, Cl, Br) were tolerated well and high yields (93–94%) were obtained (Table 2, entries 2–4). The substrate with an electron-withdrawing nitro group also afforded the product with an excellent yield (97%) within 45 minutes (Table 2, entry 5). The protocol was also compatible with substrates with electron-donating substituents such as methyl and methoxy groups (Table 2, entries 6 and 7) which achieved high yields (90–91%) within one hour to have the substrates completely consumed. We attributed this to the weakened acidity of the indole NH, which is caused by the electron-donating substituents. In particular, antihistamine reagent **A** (**2a**) was synthesized in a total yield of 67% (Table 2, entry 8), while it took four steps to afford this molecule with only 24% total yield in the original literature [12].

**Table 2:** Synthesis of 5,6-dihydroindolo[1,2-*a*]quinoxalines by CuI-catalyzed intramolecular N-arylation.^a^

entry	substrate		product		yield (%)^b^

1	**1a**	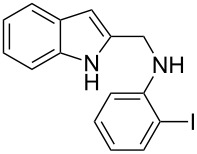	**2a**	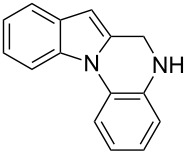	92
2	**1b**	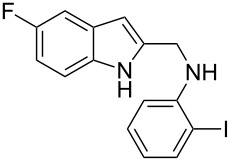	**2b**	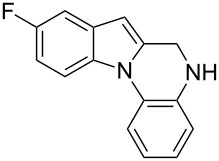	94
3	**1c**	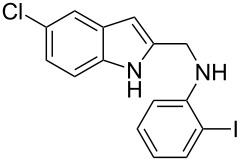	**2c**	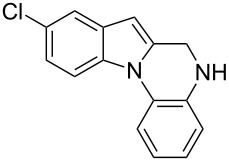	93
4	**1d**	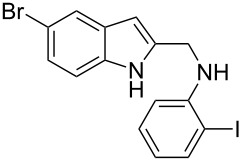	**2d**	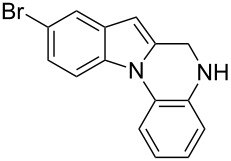	94
5	**1e**	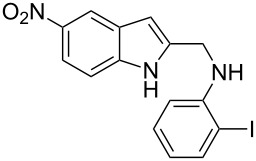	**2e**	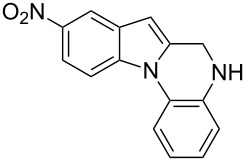	97
6^c^	**1f**	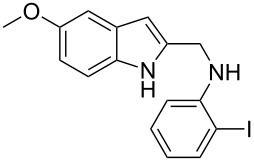	**2f**	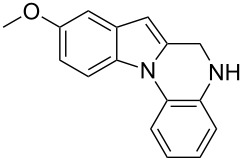	90
7^c^	**1g**	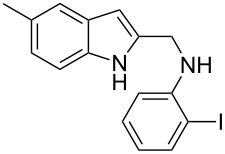	**2g**	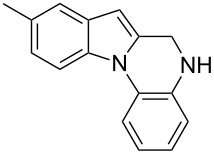	91
8	**1h**	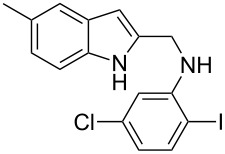	**2h**	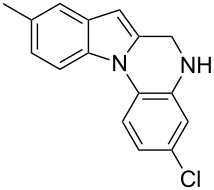	92
9	**1i**	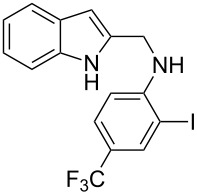	**2i**	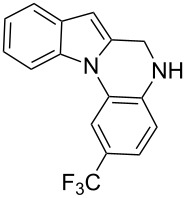	96
10	**1j**	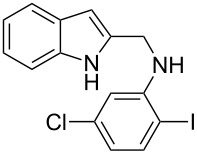	**2j**	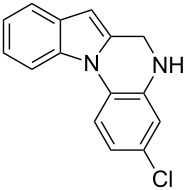	95
11	**1k**	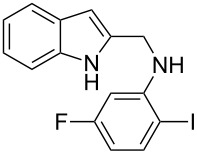	**2k**	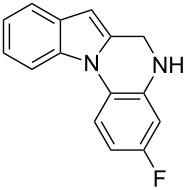	91
12^c^	**1l**	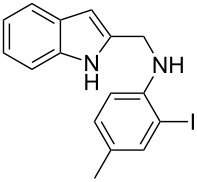	**2l**	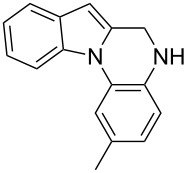	88
13^d^	**1m**	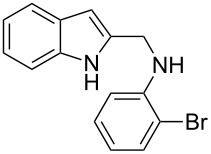	**2a**	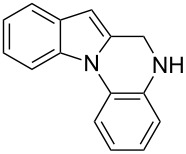	85
14^d^	**1n**	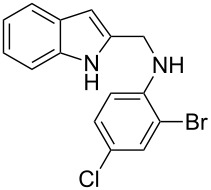	**2n**	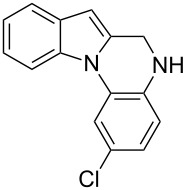	84
15^d^	**1o**	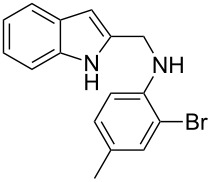	**2l**	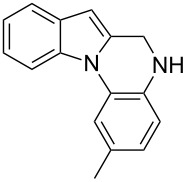	83
16^d^	**1p**	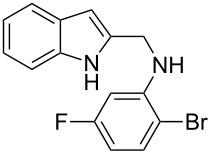	**2k**	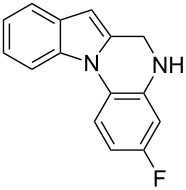	86

^a^Unless noted, reactions were performed with **1** (0.25 mmol), CuI (0.025 mmol), L-proline (0.05 mmol) and K_2_CO_3_ (0.5 mmol) in DMSO (2 mL) at 90 °C (MW irradiation, sealed vessel at fixed power, 30 W). ^b^Isolated yield. ^c^The reaction was run for one hour. ^d^The reaction was performed at 140 °C with Cs_2_CO_3_ as the base for one hour.

Next, the approach was investigated with respect to the structural variation of the aryl iodide moiety. The reaction proceeded smoothly with high yields for substrates with electron deficient aryl iodides, whereas methyl-substituted substrate **2l** required additional 15 minutes to complete the transformation (Table 2, entries 9–12). This indicated that electron-deficient aryl iodides display a better reactivity than electron-rich ones. A further extension of the methodology to various substrates linked with bromobenzene was also briefly investigated. In light of a lower reactivity of bromobenzenes compared to iodobenzenes in C–N coupling reactions, we replaced K_2_CO_3_ with the stronger base Cs_2_CO_3_, and elevated the reaction temperature from 90 °C to 140 °C. As a result, all these bromo-substituted substrates provided the desired products with good yields within one hour (Table 2, entries 13–16). These findings broadened the substrate scope of the methodology.

## Conclusion

In summary, we have developed a simple and efficient CuI-catalyzed methodology for the synthesis of 5,6-dihydroindolo[1,2-*a*]quinoxaline derivatives. This approach rapidly achieved the tetracyclic products with good to excellent yields in short reaction time under microwave irradiation. We anticipate that these important heterocyclic compounds that incorporate the bioactive indole motif may find their pharmaceutical applications after further investigations.

## Experimental

General procedure for the synthesis of 5,6-dihydroindolo[1,2-*a*]quinoxalines: A high-pressure microwave vessel was loaded with **1** (0.25 mmol, 1.0 equiv), CuI (0.025 mmol, 4.8 mg, 0.1 equiv), L-proline (0.05 mmol, 5.8 mg, 0.2 equiv), and the base indicated (0.5 mmol, 2.0 equiv) in DMSO (2 mL). The vessel was degassed, refilled with argon, and sealed. The mixture was heated to the temperature indicated for the indicated time under microwave irradiation (fixed power, 30 W). After cooling, the reaction mixture was washed with water, and then extracted with ethyl acetate. The organic extracts were washed with brine, dried over Na_2_SO_4_, and concentrated. The residue was purified by flash chromatography (petroleum ether/EtOAc 16:1 as eluent) to give **2**.

## Supporting Information

File 1 General information, experimental details, characterization data and copies of ^1^H and ^13^C NMR spectra.
