# Supplementary material for: Microwave-assisted synthesis of 5,6-dihydroindolo[1,2-a]quinoxaline derivatives through copper-catalyzed intramolecular N-arylation
Source: Beilstein J Org Chem. 2013 Nov 14;9:2463–9. doi: 10.3762/bjoc.9.285 (PMC3869252; doi:10.3762/bjoc.9.285)

Supporting information

for

Microwave-assisted synthesis of 5,6-dihydroindolo[1,2-*a*]quinoxaline derivatives by copper-catalyzed intramolecular N-arylation

Fei Zhao, Lei Zhang, Hailong Liu, Shengbin Zhou and Hong Liu*

Address: CAS Key Laboratory of Receptor Research, Shanghai Institute of Materia Medica, Chinese Academy of Sciences, 555 Zuchongzhi Road, Shanghai 201203, P. R. China

Email: Hong Liu* - hliu@mail.shcnc.ac.cn
* Corresponding author

General information, experimental details, characterization data and copies of ^{1}H and ^{13}C NMR spectra

General information…………………………………………………..………….. S2

Preparation and characterization data of the materials (**1a**–**1p**) ……………….. S2–S7

Preparation and characterization data of compounds **2** ………………………. S7–S11

^{1}H and ^{13}C NMR spectra …………………………………………………….. S12–S40

General information

The reagents were purchased from commercial suppliers and used without further purification. All of the microwave-assisted reactions were performed in sealed tubes (capacity 10 mL) under nitrogen atmosphere under microwave heating system (CEM Corp.) at the specified temperature using the standard mode of operation. CH_2Cl_2 used in reactions was reagent grade and distilled from CaH_2. Analytical thin-layer chromatography (TLC) was performed on HSGF 254 (0.15–0.2 mm thickness), visualized by irradiation with UV light (254 nm). Column chromatography was performed using silica gel FCP 200–300. Melting points were measured with a micro melting point apparatus. Nuclear magnetic resonance spectra were recorded on a Brucker AMX-300 or 400 MHz instrument (TMS as IS). Chemical shifts were reported in parts per million (ppm, δ) downfield from tetramethylsilane. Proton coupling patterns were described as singlet (s), doublet (d), triplet (t), quartet (q), multiplet (m), and broad (br). Low- and high-resolution mass were measured by the EI method with a Tsou-EI mass spectrometer.

Preparation and characterization data of the materials (1a–1p)

Procedure for the preparation of *N*-(1*H*-indol-2-ylmethyl)-2-iodoaniline (1a).

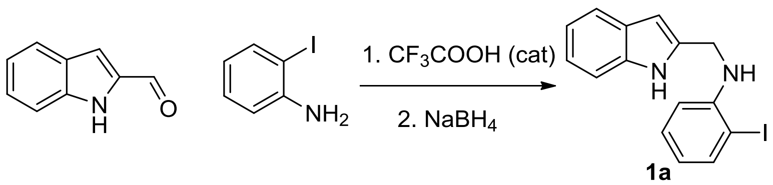

1a

A mixture of 1*H*-indole-2-carbaldehyde (1 mmol, 145.16 mg, 1.0 equiv) and 2-iodoaniline (1.1 mmol, 240.93 mg, 1.1 equiv) was dissolved in anhydrous dichloromethane (20 mL), trifluoroacetic acid (0.2 mmol, 15 µL, 0.2 equiv) was added under nitrogen, and the resulting mixture was heated to reflux for 4 h. Then the solvent was evaporated, and the residue was dissolved in anhydrous methanol (15 mL), $NaBH_4$ (4 mmol, 151 mg, 4.0 equiv) was added portionwise. After addition, the mixture was stirred for 30 min and concentrated under vacuum, the reaction mixture was washed with a saturated solution of NH_4Cl, and then extracted with ethyl

acetate. The organic extracts were washed with brine, dried over Na_2SO_4, and concentrated. The residue was purified by flash chromatography (petroleum ether/EtOAc = 16:1 as eluent) to give **1a**. White solid (299.4 mg, 86%). Mp 93–95 °C. ^{1}H NMR (400 MHz, $CDCl_3$) δ 8.29 (s, 1H), 7.71 (dd, *J* = 7.8, 1.4 Hz, 1H), 7.62–7.59 (m, 1H), 7.33 (dd, *J* = 8.0, 0.8 Hz, 1H), 7.21–7.08 (m, 3H), 6.67 (dd, *J* = 8.2, 1.3 Hz, 1H), 6.57–6.46 (m, 2H), 4.63 (s, 1H), 4.56 (d, *J* = 4.3 Hz, 2H); ^{13}C NMR (126 MHz, $CDCl_3$) δ 147.1, 139.2, 136.1, 129.8, 128.7, 121.9, 120.4, 120.1, 119.9, 111.5, 111.0, 100.4, 85.9, 42.7; EIMS (*m*/*z*, relative intensity): 348 (M^+, 12), 221 (26), 130 (100), 110 (10); HRMS (EI) calcd for $C_{15}H_{13}IN_2$ (M^+) 348.0123, found: 348.0122.

Compounds 1b–1p were prepared following the similar procedure carried out for 1a.

***N*-(5-fluoro-1*H*-indol-2-ylmethyl)-2-iodoaniline (1b):** White solid (307.6 mg, 84%). Mp 80–81 °C. ^{1}H NMR (400 MHz, $CDCl_3$) δ 8.24 (s, 1H), 7.69 (dd, *J* = 7.8, 1.3 Hz, 1H), 7.23–7.11 (m, 3H), 6.88 (td, *J* = 9.1, 2.5 Hz, 1H), 6.61 (dd, *J* = 8.2, 1.1 Hz, 1H), 6.53–6.45 (m, 1H), 6.40 (s, 1H), 4.50 (s, 2H); ^{13}C NMR (126 MHz, $CDCl_3$) δ 157.1 (d, $J_{C–F}$ = 234.4 Hz), 146.0, 138.2, 137.0, 131.5, 128.8, 128.1 (d, $J_{C–F}$ = 10.3 Hz), 119.1, 110.6 (d, $J_{C–F}$ = 9.8 Hz), 110.5, 109.1 (d, $J_{C–F}$ = 26.2 Hz), 104.2 (d, $J_{C–F}$ = 23.4 Hz), 99.3 (d, $J_{C–F}$ = 4.5 Hz), 84.9, 41.7; EIMS (*m*/*z*, relative intensity): 366 (M^+, 14), 239 (34), 219 (21), 148 (100); HRMS (EI) calcd for $C_{15}H_{12}FIN_2$ (M^+) 366.0029, found: 366.0030.

***N*-(5-chloro-1*H*-indol-2-ylmethyl)-2-iodoaniline (1c):** White solid (325.2 mg, 85%). Mp 83–84 °C. ^{1}H NMR (400 MHz, $CDCl_3$) δ 8.27 (s, 1H), 7.68 (dd, *J* = 7.8, 1.5 Hz, 1H), 7.52 (d, *J* = 2.0 Hz, 1H), 7.19–7.05 (m, 3H), 6.59 (dd, *J* = 8.2, 1.4 Hz, 1H), 6.53–6.44 (m, 1H), 6.38 (s, 1H), 4.61 (s, 1H), 4.50 (d, *J* = 2.4 Hz, 2H); ^{13}C NMR (126 MHz, $CDCl_3$) δ 147.0, 139.2, 137.7, 134.3, 129.81, 129.78, 125.6, 122.1, 120.1, 119.8, 112.0, 111.4, 99.8, 85.9, 77.4, 77.2, 76.9, 42.6; EIMS (*m*/*z*, relative intensity): 384 (M^+, (Cl^{37}), 3), 382 (M^+, (Cl^{35}), 10), 253 (58), 219 (50), 179 (100), 164 (56); HRMS (EI) calcd for $C_{15}H_{12}ClIN_2$ (M^+) 381.9734, found: 381.9727.

***N*-(5-bromo-1*H*-indol-2-ylmethyl)-2-iodoaniline (1d):** White solid (354.5 mg, 83%). Mp 87–88 °C. ^{1}H NMR (400 MHz, $CDCl_3$) δ 8.31 (s, 1H), 7.71–7.63 (m, 2H), 7.24–7.18 (m, 1H), 7.17–7.09 (m, 2H), 6.59 (dd, *J* = 8.2, 1.4 Hz, 1H), 6.52–6.45 (m, 1H), 6.40–6.34 (m, 1H), 4.62 (s, 1H), 4.51 (s, 2H); ^{13}C NMR (126 MHz, $CDCl_3$) δ 147.0, 139.2, 137.6, 134.6, 130.5, 129.8, 124.62, 122.8, 120.1, 113.2, 112.4, 111.4, 99.7, 85.9, 42.6; EIMS (*m*/*z*, relative intensity): 428 (M^+, (Br^{81}), 10), 426 (M^+, (Br^{79}), 10), 301 (Br^{81}, 31), 299 (Br^{79}, 31), 208 (100), 129 (28), 110 (24); HRMS (EI) calcd for $C_{15}H_{12}BrIN_2$ (M^+) 425.9229, found: 425.9225.

***N*-(5-nitro-1*H*-indol-2-ylmethyl)-2-iodoaniline (1e):** Yellow solid (346.0 mg, 88%). Mp 175–177 °C. ^{1}H NMR (500 MHz, DMSO) δ 11.79 (s, 1H), 8.48 (d, *J* = 2.1 Hz, 1H), 7.95 (dd, *J* = 9.0, 2.2 Hz, 1H), 7.65 (dd, *J* = 7.7, 1.1 Hz, 1H), 7.51 (d, *J* = 9.0 Hz, 1H), 7.16–7.03 (m, 1H), 6.64 (d, *J* = 8.0 Hz, 1H), 6.60 (s, 1H), 6.46–6.34 (m, 1H), 5.54 (t, *J* = 5.9 Hz, 1H), 4.59 (d, *J* = 5.9 Hz, 2H); ^{13}C NMR (126 MHz, DMSO) δ 147.2, 142.1, 140.6, 139.5, 138.9, 129.3, 127.4, 118.7, 116.7, 116.2, 111.5, 111.1, 101.7, 85.0, 40.9; EIMS (*m*/*z*, relative intensity): 393 (M^+, 20), 266 (58), 219 (84), 175 (100); HRMS (EI) calcd for $C_{15}H_{12}IN_3O_2$ (M^+) 392.9974, found: 392.9963.

***N*-(5-methoxy-1*H*-indol-2-ylmethyl)-2-iodoaniline (1f):** White solid (302.6 mg. 80%). Mp 117–118 °C. ^{1}H NMR (400 MHz, $CDCl_3$) δ 8.21 (s, 1H), 7.73 (dd, *J* = 7.8, 1.5 Hz, 1H), 7.26–7.14 (m, 2H), 7.09 (d, *J* = 2.4 Hz, 1H), 6.85 (dd, *J* = 8.8, 2.5 Hz, 1H), 6.68 (dd, *J* = 8.2, 1.4 Hz, 1H), 6.57–6.47 (m, 1H), 6.43 (s, 1H), 4.64 (s, 1H), 4.55 (s, 2H), 3.88 (s, 3H); ^{13}C NMR (126 MHz, $CDCl_3$) δ 154.4, 147.1, 139.2, 136.8, 131.2, 129.8, 129.2, 119.9, 112.0, 111.7, 111.4, 102.3, 100.2, 85.8, 56.0, 42.8; EIMS (*m*/*z*, relative intensity): 378 (M^+, 16), 251 (14), 219 (12), 160 (100); HRMS (EI) calcd for $C_{16}H_{15}IN_2O$ (M^+)378.0229, found: 378.0222.

***N*-(5-methyl-1*H*-indol-2-ylmethyl)-2-iodoaniline (1g):** White solid (293.4 mg, 81%). Mp 118–120 °C. ^{1}H NMR (400 MHz, $CDCl_3$) δ 8.18 (s, 1H), 7.77 (dd, *J* = 7.8, 1.4 Hz, 1H), 7.45 (s, 1H), 7.29–7.19 (m, 2H), 7.07 (dd, *J* = 8.2, 1.1 Hz, 1H), 6.71 (dd, *J* = 8.2, 1.2 Hz, 1H), 6.62–6.52 (m, 1H), 6.46 (d, *J* = 1.0 Hz, 1H), 4.67 (s, 1H), 4.56 (d, *J* = 4.7 Hz, 2H), 2.52 (s, 3H); ^{13}C NMR (101 MHz, $CDCl_3$) δ 146.1, 138.2, 135.1, 133.3,

128.7, 128.3, 127.9, 122.4, 119.1, 118.8, 110.43, 109.7, 98.8, 84.8, 41.7, 20.6; EIMS (*m*/*z*, relative intensity): 362 (M^+, 12), 235 (17), 219 (6), 144 (100); HRMS (EI) calcd for $C_{16}H_{15}IN_2$ (M^+) 362.0280, found: 362.0289.

***N*-(5-methyl-1*H*-indol-2-ylmethyl)-5-chloro-2-iodoaniline (1h):** White solid (289.6 mg, 73%). Mp 99–101 °C. ^{1}H NMR (400 MHz, $CDCl_3$) δ 8.08 (s, 1H), 7.59 (d, *J* = 8.3 Hz, 1H), 7.41 (d, *J* = 0.7 Hz, 1H), 7.28–7.21 (m, 1H), 7.04 (dd, *J* = 8.3, 1.4 Hz, 1H), 6.65 (d, *J* = 2.3 Hz, 1H), 6.52 (dd, *J* = 8.3, 2.3 Hz, 1H), 6.43 (d, *J* = 1.1 Hz, 1H), 4.63 (t, *J* = 4.9 Hz, 1H), 4.48 (d, *J* = 5.2 Hz, 2H), 2.47 (s, 3H); ^{13}C NMR (101 MHz, $CDCl_3$) δ 148.0, 139.7, 135.8, 135.1, 134.5, 129.4, 128.8, 123.7, 120.2, 119.6, 111.2, 110.7, 100.6, 82.6, 42.5, 21.6; EIMS (*m*/*z*, relative intensity): 398 (M^+, (Cl^{37}), 5), 396 (M^+, (Cl^{35}), 15), 267 (76), 144 (100); HRMS (EI) calcd for $C_{16}H_{14}ClIN_2$ (M^+) 395.9890, found: 395.9891.

***N*-(1*H*-indol-2-ylmethyl)-4-trifluoromethyl-2-iodoaniline (1i):** White solid (308 mg, 74%). Mp 124–126 °C. ^{1}H NMR (400 MHz, $CDCl_3$) δ 8.21 (s, 1H), 7.92 (d, *J* = 1.4 Hz, 1H), 7.60 (d, *J* = 7.8 Hz, 1H), 7.40 (dd, *J* = 8.6, 1.5 Hz, 1H), 7.36–7.33 (m, 1H), 7.21–7.09 (m, 2H), 6.67 (d, *J* = 8.6 Hz, 1H), 6.50 (d, *J* = 1.1 Hz, 1H), 4.96 (s, 1H), 4.60 (d, *J* = 5.2 Hz, 2H); ^{13}C NMR (126 MHz, $CDCl_3$) δ 149.5, 136.2 (q, $J_{C–F}$ = 3.9 Hz), 134.8, 128.5, 127.1 (q, $J_{C–F}$ = 3.8 Hz), 123.7 (q, $J_{C–F}$ = 270.8 Hz), 122.3, 121.3 (q, $J_{C–F}$ = 32.3 Hz), 120.6, 120.3, 111.1, 110.2, 101.1, 84.2, 42.5; EIMS (*m*/*z*, relative intensity): 416 (M^+, 12), 289 (17), 130 (100); HRMS (EI) calcd for $C_{16}H_{12}F_3IN_2$ (M^+) 415.9997, found: 415.9984.

***N*-(1*H*-indol-2-ylmethyl)-5-chloro-2-iodoaniline (1j):** White solid (298.5 mg, 78%). Mp 94–96 °C. ^{1}H NMR (400 MHz, CDCl3) δ 8.21 (s, 1H), 7.64 (d, J = 7.9 Hz, 1H), 7.61 (d, *J* = 8.3 Hz, 1H), 7.38 (dd, *J* = 8.0, 0.8 Hz, 1H), 7.26–7.20 (m, 1H), 7.20–7.14 (m, 1H), 6.67 (d, *J* = 2.3 Hz, 1H), 6.57–6.51 (m, 2H), 4.67 (s, 1H), 4.52 (d, *J* = 4.9 Hz, 2H); ^{13}C NMR (101 MHz, $CDCl_3$) δ 148.0, 139.7, 136.2, 135.8, 135.1, 128.5, 122.2, 120.6, 120.2, 119.7, 111.2, 111.1, 101.0, 82.7, 42.5; EIMS (*m*/*z*, relative intensity): 384 (M^+, (Cl^{37}), 2), 382 (M^+, (Cl^{35}), 5), 253 (35), 130 (100), 127 (93); HRMS (EI) calcd for $C_{15}H_{12}ClIN_2$ (M^+) 381.9734, found: 381.9732.

***N*-(1*H*-indol-2-ylmethyl)-5-fluoro-2-iodoaniline (1k):** White solid (293.0 mg, 80%). Mp 101–103 °C. ^{1}H NMR (400 MHz, $CDCl_3$) δ 8.23 (s, 1H), 7.67–7.61 (m, 2H), 7.40–7.35 (m, 1H), 7.25–7.14 (m, 2H), 6.53 (dd, *J* = 2.0, 0.9 Hz, 1H), 6.44 (dd, *J* = 11.3, 2.8 Hz, 1H), 6.35–6.27 (m, 1H), 4.71 (s, 1H), 4.54 (s, 2H); ^{13}C NMR (126 MHz, $CDCl_3$) δ 164.5 (d, $J_{C–F}$ = 244.9 Hz), 148.5 (d, $J_{C–F}$ = 10.8 Hz), 139.6 (d, $J_{C–F}$= 9.6 Hz), 136.2, 135.1, 128.6, 122.2, 120.6, 120.2, 111.0, 106.7 (d, $J_{C–F}$ = 22.5 Hz), 101.0, 99.1 (d, $J_{C–F}$ = 27.3 Hz), 78.3, 42.6; EIMS (*m*/*z*, relative intensity): 366 (M^+, 21), 237 (100), 110 (54); HRMS (EI) calcd for $C_{15}H_{12}FIN_2$ (M^+) 366.0029, found: 366.0020.

***N*-(1*H*-indol-2-ylmethyl)-4-methyl-2-iodoaniline (1l):** White solid (318.7 mg, 88%). Mp 83–85 °C. ^{1}H NMR (300 MHz, $CDCl_3$) δ 8.19 (s, 1H), 7.60 (dd, *J* = 7.6, 0.5 Hz, 1H), 7.31 (d, *J* = 7.7 Hz, 1H), 7.22–7.08 (m, 3H), 7.01 (d, *J* = 8.2 Hz, 1H), 6.58 (dd, *J* = 8.2, 2.5 Hz, 1H), 6.44 (d, *J* = 0.9 Hz, 1H), 4.40 (s, 2H), 3.89 (s, 1H); ^{13}C NMR (126 MHz, $CDCl_3$) δ 146.8, 136.4, 136.0, 130.8, 130.0, 128.5, 123.4, 121.9, 120.4, 120.0, 113.3, 111.0, 101.8, 100.3, 42.3, 26.9; EIMS (*m*/*z*, relative intensity): 362 (M^+, 22), 233 (6), 130 (100); HRMS (EI) calcd for $C_{16}H_{15}IN_2$ (M^+) 362.0280, found: 362.0273.

***N*-(1*H*-indol-2-ylmethyl)-2-bromoaniline (1m):** White solid (247 mg, 82%). Mp 107–108 °C. ^{1}H NMR (400 MHz, $CDCl_3$) δ 8.24 (s, 1H), 7.69–7.64 (m, 1H), 7.54 (dd, *J* = 7.9, 1.5 Hz, 1H), 7.39–7.33 (m, 1H), 7.26–7.16 (m, 3H), 6.77 (dd, *J* = 8.2, 1.5 Hz, 1H), 6.73–6.66 (m, 1H), 6.54–6.50 (m, 1H), 4.80 (s, 1H), 4.56 (s, 2H); ^{13}C NMR (126 MHz, $CDCl_3$) δ 143.8, 135.1, 135.0, 131.6, 127.8, 127.6, 120.9, 119.4, 119.1, 118.0, 111.1, 110.0, 109.1, 99.3, 41.3; EIMS (*m*/*z*, relative intensity): 302 (M^+, (Br^{81}), 9), 300 (M^+, (Br^{79}), 9), 221 (Br^{81}, 12), 219 (Br^{79}, 12), 130 (100); HRMS (EI) calcd for $C_{15}H_{13}BrN_2$ (M^+) 300.0262, found: 300.0257.

***N*-(1*H*-indol-2-ylmethyl)-2-bromo-4-chloroaniline (1n):** White solid (241.7 mg, 72%). Mp 135–136 °C. ^{1}H NMR (300 MHz, CD_3OD) δ 7.46–7.38 (m, 2H), 7.31–7.25 (m, 1H), 7.08–7.00 (m, 2H), 6.98–6.93 (m, 1H), 6.69 (d, *J* = 8.8 Hz, 1H), 6.31 (s, 1H), 4.51 (s, 2H); ^{13}C NMR (101 MHz, MeOD) δ 145.5, 138.1, 137.7, 132.5, 129.8, 129.3,

122.5, 122.1, 120.8, 120.1, 113.6, 111.8, 110.1, 100.6, 42.5; EIMS (*m*/*z*, relative intensity): 336 (M^+, (Br^{81}), 6), 334 (M^+, (Br^{79}), 6), 130 (100); HRMS (EI) calcd for $C_{15}H_{12}BrClN_2$ (M^+) 333.9872, found: 333.9866.

***N*-(1*H*-indol-2-ylmethyl)-2-bromo-4-methylaniline (1o):** White solid (261.6 mg, 83%). Mp 132–133 °C. ^{1}H NMR (400 MHz, $CDCl_3$) δ 8.26 (s, 1H), 7.63 (d, *J* = 7.7 Hz, 1H), 7.37–7.28 (m, 2H), 7.24–7.10 (m, 2H), 6.96 (dd, *J* = 8.2, 1.4 Hz, 1H), 6.65 (d, *J* = 8.2 Hz, 1H), 6.49 (d, *J* = 0.9 Hz, 1H), 4.52 (s, 2H), 2.26 (s, 3H); ^{13}C NMR (101 MHz, $CDCl_3$) δ 142.5, 136.4, 136.0, 132.9, 129.3, 128.7, 121.8, 120.4, 120.0, 112.1, 111.0, 110.1, 100.1, 42.5, 20.2; EIMS (*m*/*z*, relative intensity): 316 (M^+, (Br^{81}), 4), 314 (M^+, (Br^{79}), 4), 279 (48), 264 (38), 187 (100), 130 (40); HRMS (EI) calcd for $C_{16}H_{15}BrN_2$ (M^+) 314.0419, found: 314.0403.

***N*-(1*H*-indol-2-ylmethyl)-2-bromo-5-fluoroaniline (1p):** White solid (239.4 mg, 75%). Mp 115–116 °C. ^{1}H NMR (500 MHz, $CDCl_3$) δ 8.17 (s, 1H), 7.65 (d, *J* = 7.8 Hz, 1H), 7.46–7.40 (m, 1H), 7.37 (d, *J* = 8.0 Hz, 1H), 7.27–7.21 (m, 1H), 7.21–7.15 (m, 1H), 6.55–6.51 (m, 1H), 6.49 (dd, *J* = 11.0, 2.8 Hz, 1H), 6.41 (td, *J* = 8.4, 2.8 Hz, 1H), 4.87 (s, 1H), 4.51 (d, *J* = 5.3 Hz, 2H); ^{13}C NMR (126 MHz, $CDCl_3$) δ 163.3 (d, $J_{C–F}$ = 244.2 Hz), 146.1 (d, $J_{C–F}$ = 11.2 Hz), 136.2, 135.2, 133.1 (d, $J_{C–F}$ = 10.0 Hz), 128.5, 122.1, 120.5, 120.2, 111.0, 105.5 (d, $J_{C–F}$ = 23.1 Hz), 104.0, 100.9, 99.5 (d, $J_{C–F}$ = 27.7 Hz), 42.1; EIMS (*m*/*z*, relative intensity): 320 (M^+, (Br^{81}), 7), 318 (M^+, (Br^{79}), 7), 239 (10), 130 (100); HRMS (EI) calcd for $C_{15}H_{12}BrFN_2$ (M^+) 318.0168, found: 318.0158.

Preparation and characterization data of of compounds 2

Procedure for the preparation of 5,6-dihydroindolo[1,2-*a*]quinoxaline (2a).

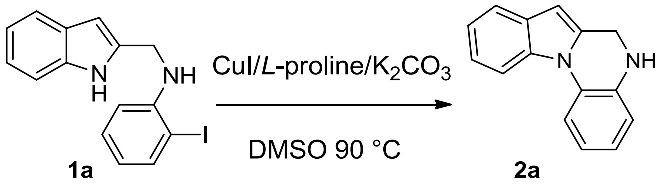

A high-pressure microwave vessel was loaded with the **1a** (0.25 mmol, 1.0 equiv), CuI (0.025 mmol, 4.8 mg, 0.1 equiv), L-proline (0.05 mmol, 5.8 mg, 0.2 equiv), and

K_2CO_3 (0.5 mmol, 69.1 mg, 2.0 equiv) in DMSO (2 mL). The vessel was degassed, refilled with argon, and sealed. The mixture was heated to 90 °C for 45 min under microwave irradiation (fixed power, 30 W). After cooling, the reaction mixture was washed with water, and then extracted with ethyl acetate. The organic extracts were washed with brine, dried over Na_2SO_4, and concentrated. The residue was purified by flash chromatography to give **2a**. White solid (92%). Mp 88–89 °C. ^{1}H NMR (400 MHz, $CDCl_3$) δ 8.03 (d, *J* = 8.1 Hz, 1H), 7.97–7.90 (m, 1H), 7.69 (d, *J* = 7.8 Hz, 1H), 7.35–7.27 (m, 1H), 7.27–7.21 (m, 1H), 7.10–7.00 (m, 2H), 6.92–6.85 (m, 1H), 6.42 (s, 1H), 4.48 (s, 2H), 4.01 (s, 1H); ^{13}C NMR (126 MHz, $CDCl_3$) δ 138.02, 134.79, 134.06, 129.90, 127.67, 124.06, 122.33, 120.97, 120.91, 120.11, 117.12, 116.13, 111.64, 98.60, 42.16; EIMS (*m*/*z*, relative intensity): 220 (M^+, 32), 218 (100), 190 (20), 109 (14); HRMS (EI) calcd for $C_{15}H_{12}N_2$ (M^+) 220.1000, found: 220.0986.

Compounds 2b−2n were prepared following the similar procedure carried out for 2a with the base, temperature and time indicated in Table 2.

9-fluoro-5,6-dihydroindolo[1,2-*a*]quinoxaline (2b): White solid (56.0 mg, 94%). Mp 78–79 °C. ^{1}H NMR (400 MHz, $CDCl_3$) δ 7.86 (dd, *J* = 9.1, 4.3 Hz, 1H), 7.79 (dd, *J* = 7.7, 1.6 Hz, 1H), 7.28–7.23 (m, 1H), 7.05–6.93 (m, 3H), 6.85 (dd, *J* = 7.4, 1.8 Hz, 1H), 6.32 (s, 1H), 4.44 (s, 2H), 3.61 (s, 1H); ^{13}C NMR (101 MHz, $CDCl_3$) δ 157.4 (d, *J* = 236.2 Hz), 136.9, 135.4, 129.7, 129.6 (d, *J* = 10.2 Hz), 126.4, 123.3, 119.2, 115.8, 115.2, 111.3 (d, *J* = 9.5 Hz), 109.2 (d, *J* = 25.6 Hz), 105.0 (d, *J* = 23.4 Hz), 97.6 (d, *J* = 4.2 Hz), 41.2; EIMS (*m*/*z*, relative intensity): 238 (M^+, 30), 236 (100), 208 (18), 118 (13); HRMS (EI) calcd for $C_{15}H_{11}FN_2$ (M^+) 238.0906, found: 238.0904.

9-chloro-5,6-dihydroindolo[1,2-*a*]quinoxaline (2c): White solid (59.2 mg, 93%). Mp 96–97 °C. ^{1}H NMR (400 MHz, $CDCl_3$) δ 7.84 (d, *J* = 8.9 Hz, 1H), 7.77 (dd, *J* = 7.7, 1.2 Hz, 1H), 7.56 (d, *J* = 2.1 Hz, 1H), 7.18 (dd, *J* = 8.9, 2.1 Hz, 1H), 7.05–6.93 (m, 2H), 6.85 (dd, *J* = 7.5, 1.5 Hz, 1H), 6.29 (s, 1H), 4.42 (s, 2H), 3.73 (s, 1H); ^{13}C NMR (101 MHz, $CDCl_3$) δ 137.0, 135.1, 131.4, 130.0, 126.2, 125.4, 123.5, 121.4, 119.4, 119.2, 116.0, 115.3, 111.5, 97.2, 41.1; EIMS (*m*/*z*, relative intensity): 256 (M^+, (Cl^{37}), 20), 254 (M^+, (Cl^{35}), 84), 252 (100), 218 (26), 190 (23); HRMS (EI) calcd for

$C_{15}H_{11}ClN_2$ (M^+) 254.0611, found: 254.0614.

9-bromo-5,6-dihydroindolo[1,2-*a*]quinoxaline (2d): White solid (70.3 mg, 94%). Mp 128–129 °C. ^{1}H NMR (400 MHz, $CDCl_3$) δ 7.84 (d, *J* = 8.9 Hz, 1H), 7.82–7.79 (m, 1H), 7.76 (d, *J* = 2.0 Hz, 1H), 7.35 (dd, *J* = 8.8, 2.0 Hz, 1H), 7.08–6.99 (m, 2H), 6.89 (dd, *J* = 7.5, 1.7 Hz, 1H), 6.33 (s, 1H), 4.47 (s, 2H), 3.73 (s, 1H); ^{13}C NMR (126 MHz, $CDCl_3$) δ 137.0, 135.0, 131.7, 130.6, 126.2, 124.0, 123.5, 122.5, 119.3, 116.1, 115.3, 113.0, 112.0, 97.1, 41.1; EIMS (*m*/*z*, relative intensity): 300 (M^+, (Br^{81}), 100), 298 (M^+, (Br^{79}), 100), 217 (42), 190 (29), 110 (22); HRMS (EI) calcd for $C_{15}H_{11}BrN_2$ (M^+) 298.0106, found: 298.0102.

9-nitro-5,6-dihydroindolo[1,2-*a*]quinoxaline (2e): Yellow solid (64.3 mg, 97%). Mp 181–182 °C. ^{1}H NMR (500 MHz, DMSO) δ 8.56 (d, *J* = 1.6 Hz, 1H), 8.13 (d, *J* = 9.2 Hz, 1H), 8.03 (dd, *J* = 9.1, 1.8 Hz, 1H), 7.89 (d, *J* = 7.9 Hz, 1H), 7.11–7.02 (m, 1H), 6.98 (d, *J* = 7.6 Hz, 1H), 6.94–6.84 (m, 1H), 6.69 (s, 1H), 6.28 (s, 1H), 4.38 (s, 2H); ^{13}C NMR (126 MHz, DMSO) δ 141.4, 139.7, 139.2, 135.8, 129.0, 125.6, 125.2, 118.8, 117.33, 117.27, 117.2, 116.2, 111.8, 100.2, 40.6; EIMS (*m*/*z*, relative intensity): 265 (M^+, 100), 233 (22), 218 (80), 190 (16); HRMS (EI) calcd for $C_{15}H_{11}N_3O_2$ (M^+) 265.0851, found: 265.0845.

9-methoxy-5,6-dihydroindolo[1,2-*a*]quinoxaline (2f): White solid (56.3 mg, 90%). Mp 109–110 °C. ^{1}H NMR (400 MHz, $CDCl_3$) δ 7.88–7.80 (m, 2H), 7.10 (d, *J* = 2.5 Hz, 1H), 7.02–6.97 (m, 2H), 6.90 (dd, *J* = 9.0, 2.6 Hz, 1H), 6.86–6.82 (m, 1H), 6.30 (s, 1H), 4.44 (s, 2H), 3.88 (s, 3H); ^{13}C NMR (126 MHz, $CDCl_3$) δ 154.8, 137.8, 135.3, 130.7, 129.3, 127.7, 123.9, 120.1, 116.6, 116.0, 112.4, 111.6, 103.2, 98.4, 55.9, 42.2; EIMS (*m*/*z*, relative intensity): 250 (M^+, 4), 248 (100), 218 (14), 205 (81); HRMS (EI) calcd for $C_{16}H_{14}N_2O$ (M^+) 250.1106, found: 250.1101.

9-methyl-5,6-dihydroindolo[1,2-*a*]quinoxaline (2g): White solid (53.3 mg, 91%). Mp 90–91 °C. ^{1}H NMR (400 MHz, $CDCl_3$) δ 7.86 (d, *J* = 8.5 Hz, 2H), 7.44 (d, *J* = 9.2 Hz, 1H), 7.09 (d, *J* = 8.5 Hz, 1H), 7.06–6.95 (m, 2H), 6.90–6.82 (m, 1H), 6.30 (s, 1H), 4.46 (s, 2H), 3.73 (s, 1H), 2.48 (s, 3H); ^{13}C NMR (126 MHz, $CDCl_3$) δ 137.9, 134.7, 132.5, 130.2, 127.8, 123.9, 123.8, 120.8, 120.1, 116.9, 116.1, 111.4, 98.2, 42.2,

21.5; EIMS (*m*/*z*, relative intensity): 234 (M^+, 28), 232 (100), 218 (8), 116 (15); HRMS (EI) calcd for $C_{16}H_{14}N_2$ (M^+) 234.1157, found: 234.1139.

3-chloro-9-methyl-5,6-dihydroindolo[1,2-*a*]quinoxaline (2h): White solid (61.8 mg, 92%). Mp 118–120 °C. ^{1}H NMR (400 MHz, $CDCl_3$) δ 7.78 (d, *J* = 8.5 Hz, 1H), 7.74 (d, *J* = 8.6 Hz, 1H), 7.43 (s, 1H), 7.10 (dd, *J* = 8.5, 1.6 Hz, 1H), 6.94 (dd, *J* = 8.6, 2.3 Hz, 1H), 6.80 (d, *J* = 2.3 Hz, 1H), 6.30 (s, 1H), 4.42 (s, 2H), 3.99 (s, 1H), 2.49 (s, 3H); ^{13}C NMR (126 MHz, $CDCl_3$) δ 138.8, 134.0, 132.3, 130.5, 130.2, 128.6, 126.2, 124.0, 120.9, 119.6, 117.5, 115.7, 111.2, 98.6, 41.9, 21.4; EIMS (*m*/*z*, relative intensity): 270 (M^+, (Cl^{37}), 22), 268 (M^+, (Cl^{35}), 69), 267 (100), 252 (7), 232 (10); HRMS (EI) calcd for $C_{16}H_{13}ClN_2$ (M^+) 268.0767, found: 268.0757.

2-(trifluoromethyl)-5,6-dihydroindolo[1,2-*a*]quinoxaline (2i): White solid (69.2 mg, 96%). Mp 98–100 °C. ^{1}H NMR (400 MHz, $CDCl_3$) δ 8.10 (d, *J* = 1.3 Hz, 1H), 7.93 (dd, *J* = 8.4, 0.6 Hz, 1H), 7.65 (d, *J* = 7.5 Hz, 1H), 7.36–7.29 (m, 1H), 7.28–7.19 (m, 2H), 6.86 (d, *J* = 8.2 Hz, 1H), 6.40 (d, *J* = 0.8 Hz, 1H), 4.51 (s, 2H), 3.57 (s, 1H); ^{13}C NMR (126 MHz, $CDCl_3$) δ 140.7, 134.0, 133.8, 130.1, 127.1, 124.7(q, $J_{C–F}$ = 269 Hz), 123.0, 121.7 (q, $J_{C–F}$ = 33 Hz), 121.5, 121.2, 121.1 (q, $J_{C–F}$ = 3.9 Hz), 115.5, 113.9 (q, $J_{C–F}$ = 3.8 Hz), 111.5, 99.5, 41.7; EIMS (*m*/*z*, relative intensity): 288 (M^+, 3), 144 (8), 130 (100); HRMS (EI) calcd for C_{16}H11F_3N_2 (M^+) 288.0874, found: 288.0868.

3-chloro-5,6-dihydroindolo[1,2-*a*]quinoxaline (2j): White solid (60.5 mg, 95%). Mp 104–105 °C. ^{1}H NMR (400 MHz, $CDCl_3$) δ 7.90 (d, *J* = 8.3 Hz, 1H), 7.77 (d, *J* = 8.6 Hz, 1H), 7.65 (dd, *J* = 7.8, 0.6 Hz, 1H), 7.32–7.25 (m, 1H), 7.24–7.18 (m, 1H), 6.94 (dd, *J* = 8.6, 2.3 Hz, 1H), 6.81 (d, *J* = 2.3 Hz, 1H), 6.38 (s, 1H), 4.43 (s, 2H), 3.95 (s, 1H); ^{13}C NMR (126 MHz, $CDCl_3$) δ 139.0, 134.1, 133.9, 129.9, 128.9, 126.1, 122.6, 121.2, 121.1, 119.7, 117.8, 115.8, 111.5, 99.0, 41.9; EIMS (*m*/*z*, relative intensity): 256 (M^+, (Cl^{37}), 20), 254 (M^+, (Cl^{35}), 72), 252 (100), 218 (22), 190 (24); HRMS (EI) calcd for $C_{15}H_{11}ClN_2$ (M^+) 254.0611, found: 254.0609.

3-fluoro-5,6-dihydroindolo[1,2-*a*]quinoxaline (2k): White solid (54.2 mg, 91%). Mp 87–88 °C. ^{1}H NMR (400 MHz, $CDCl_3$) δ 7.86 (dd, *J* = 8.3, 0.6 Hz, 1H), 7.76–

7.69 (m, 1H), 7.60 (dd, J = 7.8, 0.5 Hz, 1H), 7.26–7.19 (m, 1H), 7.19–7.13 (m, 1H), 6.62 (td, J = 8.6, 2.8 Hz, 1H), 6.48 (dd, J = 9.5, 2.8 Hz, 1H), 6.32 (d, J = 0.7 Hz, 1H), 4.35 (s, 2H), 3.79 (s, 1H); ^{13}C NMR (126 MHz, $CDCl_3$) δ 158.3 (d, $J_{C–F}$ = 241.9 Hz), 138.4 (d, $J_{C–F}$ = 10.1 Hz), 133.1, 132.9, 128.7, 122.8, 121.4, 120.0, 119.9, 116.7 (d, $J_{C–F}$ = 9.7 Hz), 110.3, 105.0 (d, $J_{C–F}$ = 22.8 Hz), 102.2 (d, $J_{C–F}$ = 25.9 Hz), 97.6, 40.9; EIMS (*m*/*z*, relative intensity): 238 (M^+, 54), 237 (100), 208 (14), 119 (13); HRMS (EI) calcd for $C_{15}H_{11}FN_2$ (M^+) 238.0906, found: 238.0897.

2-methyl-5,6-dihydroindolo[1,2-*a*]quinoxaline (2l): White solid (51.5 mg, 88%). Mp 79–81 °C. ^{1}H NMR (400 MHz, $CDCl_3$) δ 8.00 (d, J = 8.3 Hz, 1H), 7.72 (d, J = 0.7 Hz, 1H), 7.65 (d, J = 7.8 Hz, 1H), 7.32–7.26 (m, 1H), 7.23–7.18 (m, 1H), 6.85 (dd, J = 7.9, 0.9 Hz, 1H), 6.79–6.75 (m, 1H), 6.38 (s, 1H), 4.42 (s, 2H), 3.53 (s, 1H), 2.42 (s, 3H); ^{13}C NMR (101 MHz, $CDCl_3$) δ 134.6, 134.0, 133.0, 128.9, 128.7, 126.7, 123.5, 121.2, 120.0, 119.8, 116.8, 115.1, 110.7, 97.5, 41.4, 20.3; EIMS (*m*/*z*, relative intensity): 234 (M^+, 57), 233(100), 218 (14), 116 (16); HRMS (EI) calcd for $C_{16}H_{14}N_2$ (M^+) 234.1157, found: 234.1141.

2-chloro-5,6-dihydroindolo[1,2-*a*]quinoxaline (2n): White solid (53.5 mg, 84%). Mp 90–91 °C. ^{1}H NMR (400 MHz, $CDCl_3$) δ 7.91 (d, J = 8.4 Hz, 1H), 7.83 (d, J = 2.2 Hz, 1H), 7.62 (d, J = 7.7 Hz, 1H), 7.31–7.26 (m, 1H), 7.23–7.14 (m, 1H), 6.97 (dd, J = 8.4, 2.2 Hz, 1H), 6.78 (d, J = 8.4 Hz, 1H), 6.38 (d, J = 0.7 Hz, 1H), 4.46 (s, 2H), 3.47 (s, 1H); ^{13}C NMR (101 MHz, $CDCl_3$) δ 136.5, 134.3, 134.0, 130.0, 128.3, 124.7, 123.7, 122.8, 121.4, 121.2, 117.2, 116.8, 111.5, 99.3, 42.1; EIMS (*m*/*z*, relative intensity): 256 (M^+, (Cl^{37}), 18), 254 (M^+, (Cl^{35}), 62), 190 (18), 126 (12); HRMS (EI) calcd for $C_{15}H_{11}ClN_2$ (M^+) 254.0611, found: 254.0600.

^{1}H and ^{13}C NMR spectra

***N*-(1*H*-indol-2-ylmethyl)-2-iodoaniline (1a)**

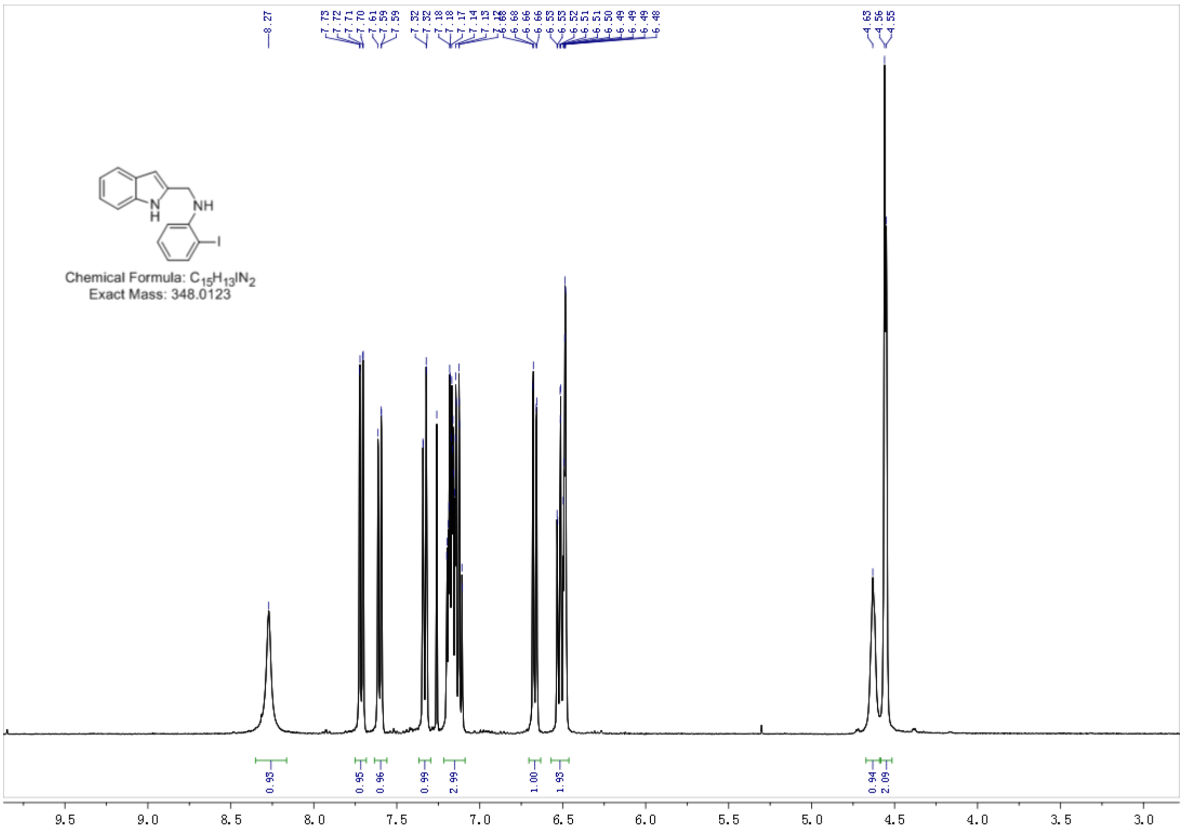

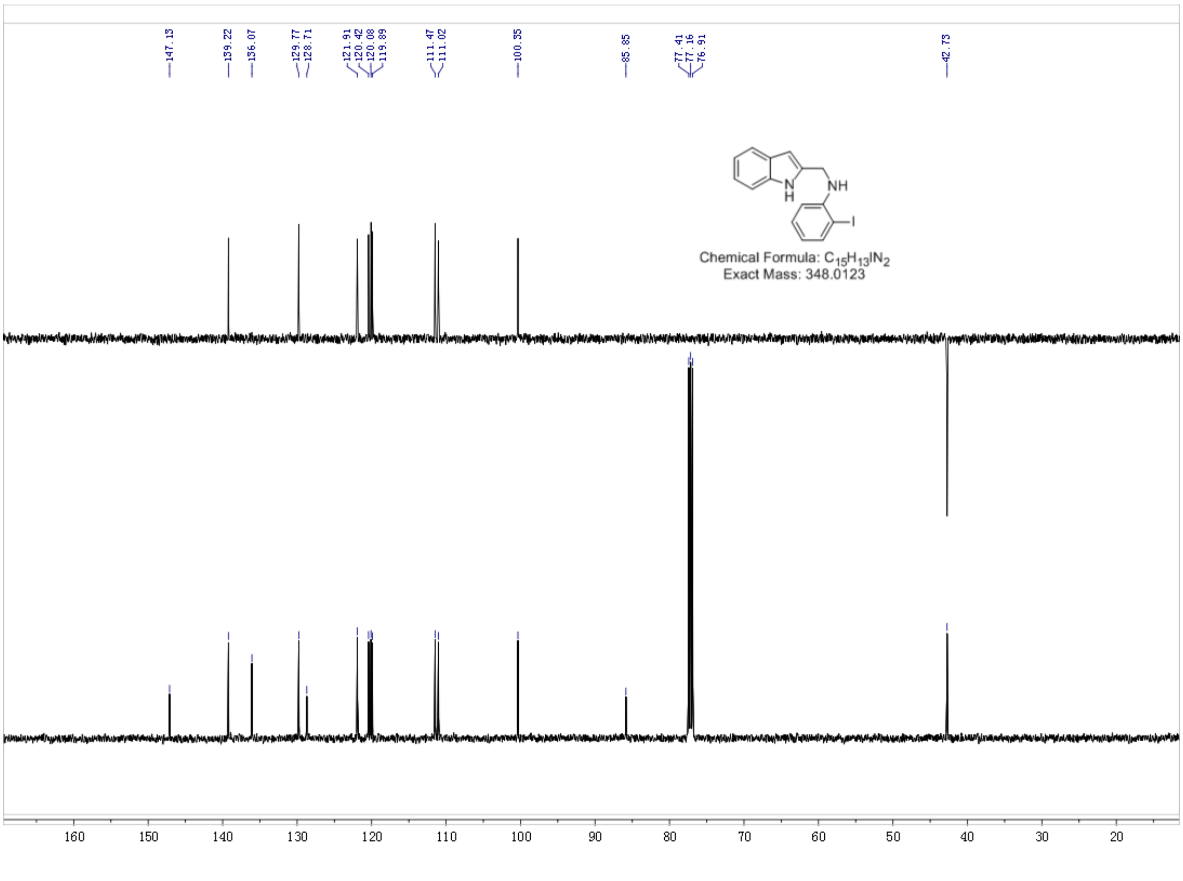

5,6-dihydroindolo[1,2-*a*]quinoxaline (2a)

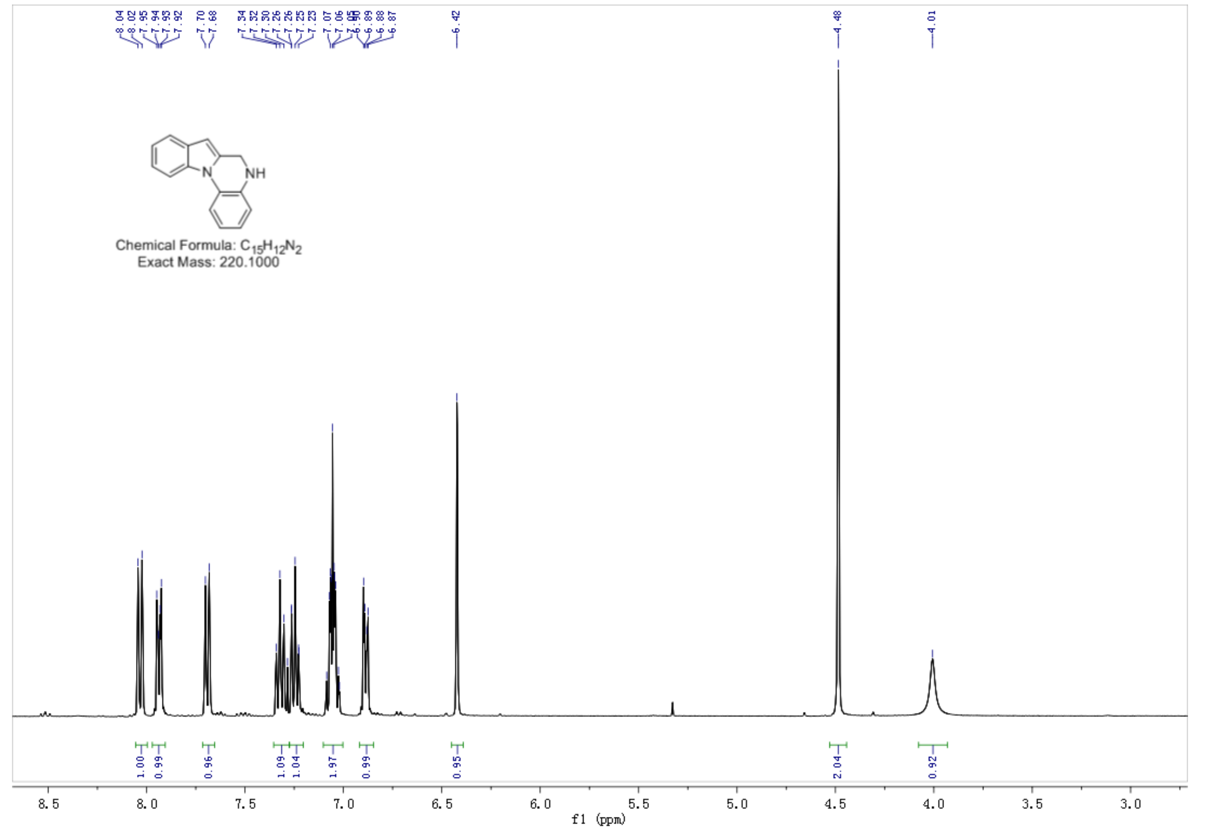

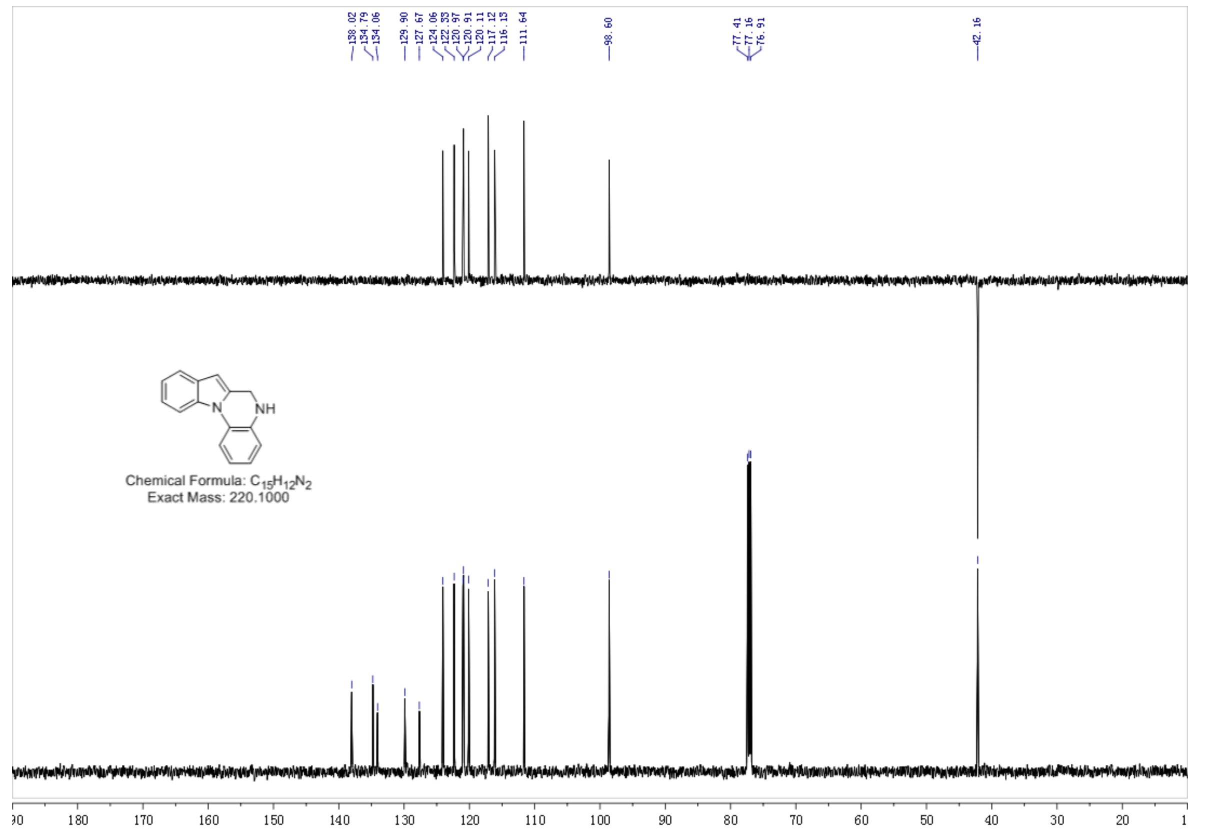

N-(5-fluoro-1H-indol-2-ylmethyl)-2-iodoaniline (1b)

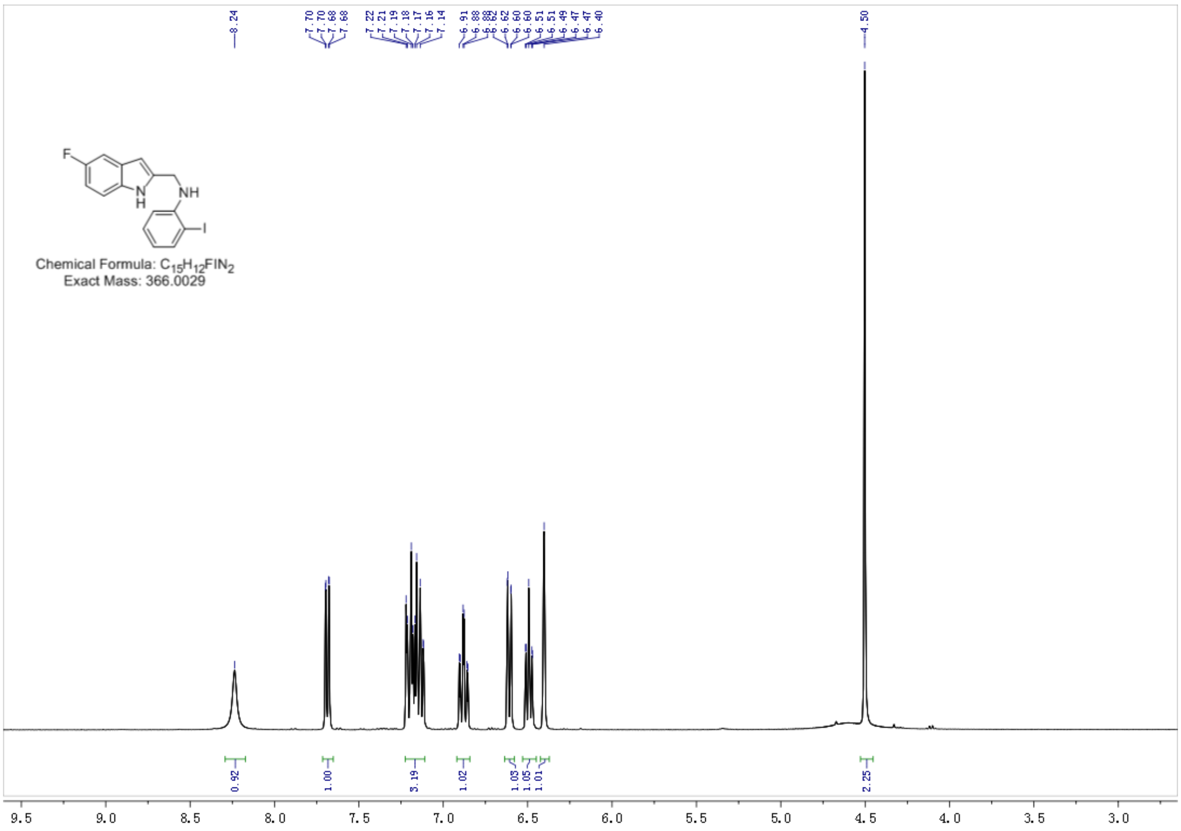

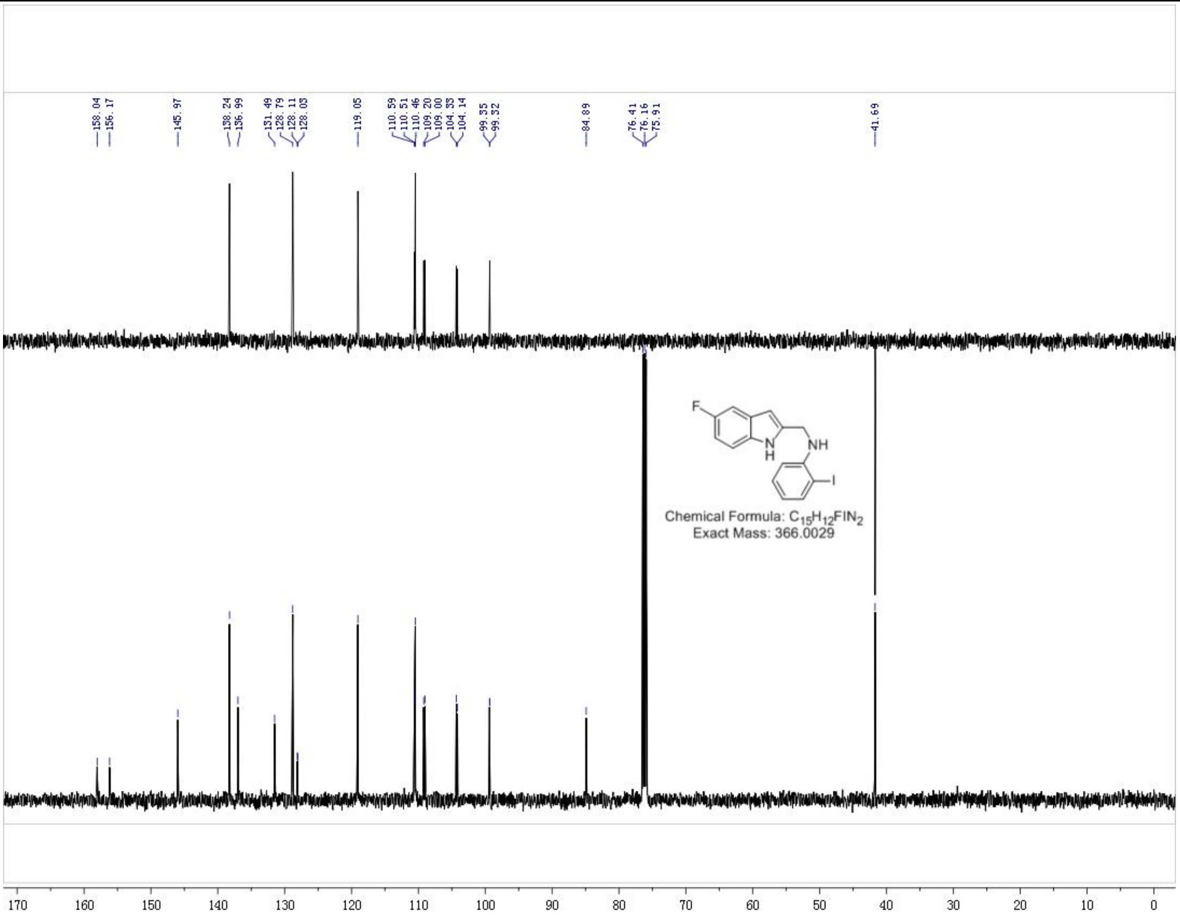

9-fluoro-5,6-dihydroindolo[1,2-*a*]quinoxaline (2b)

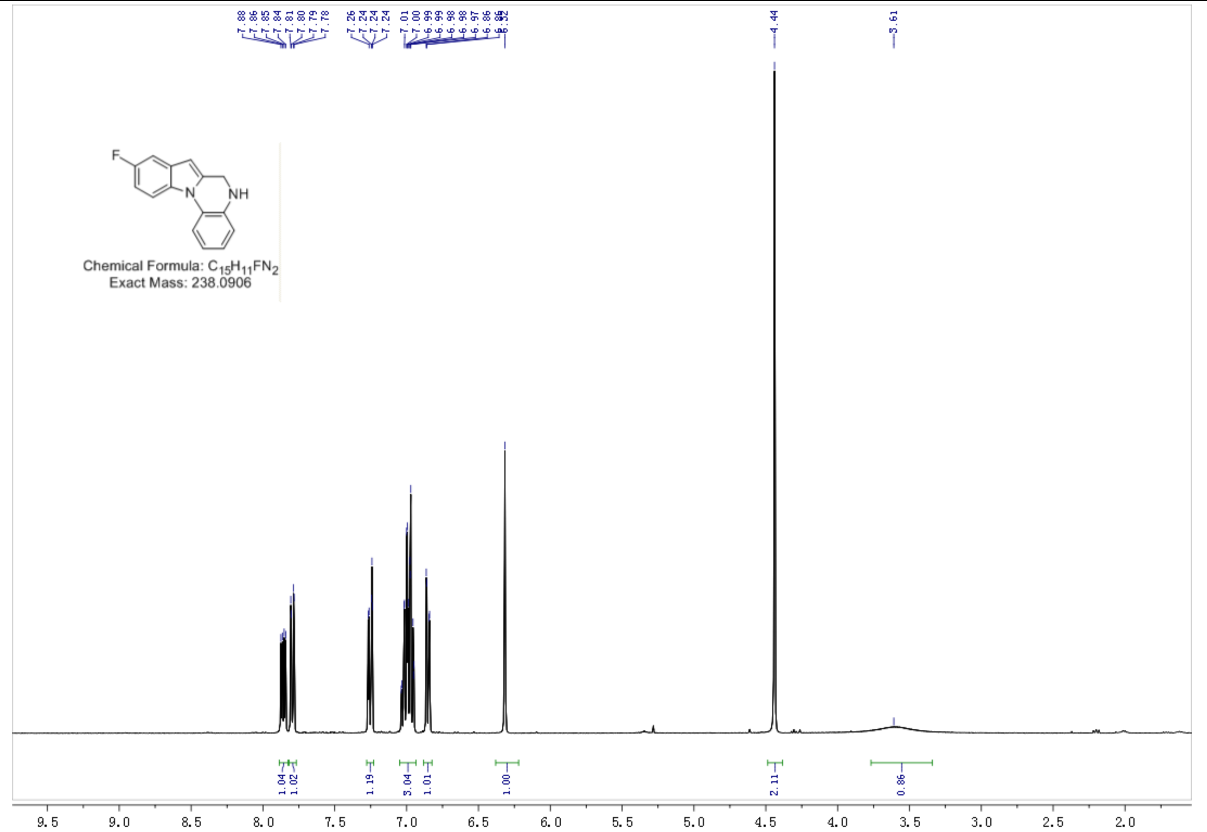

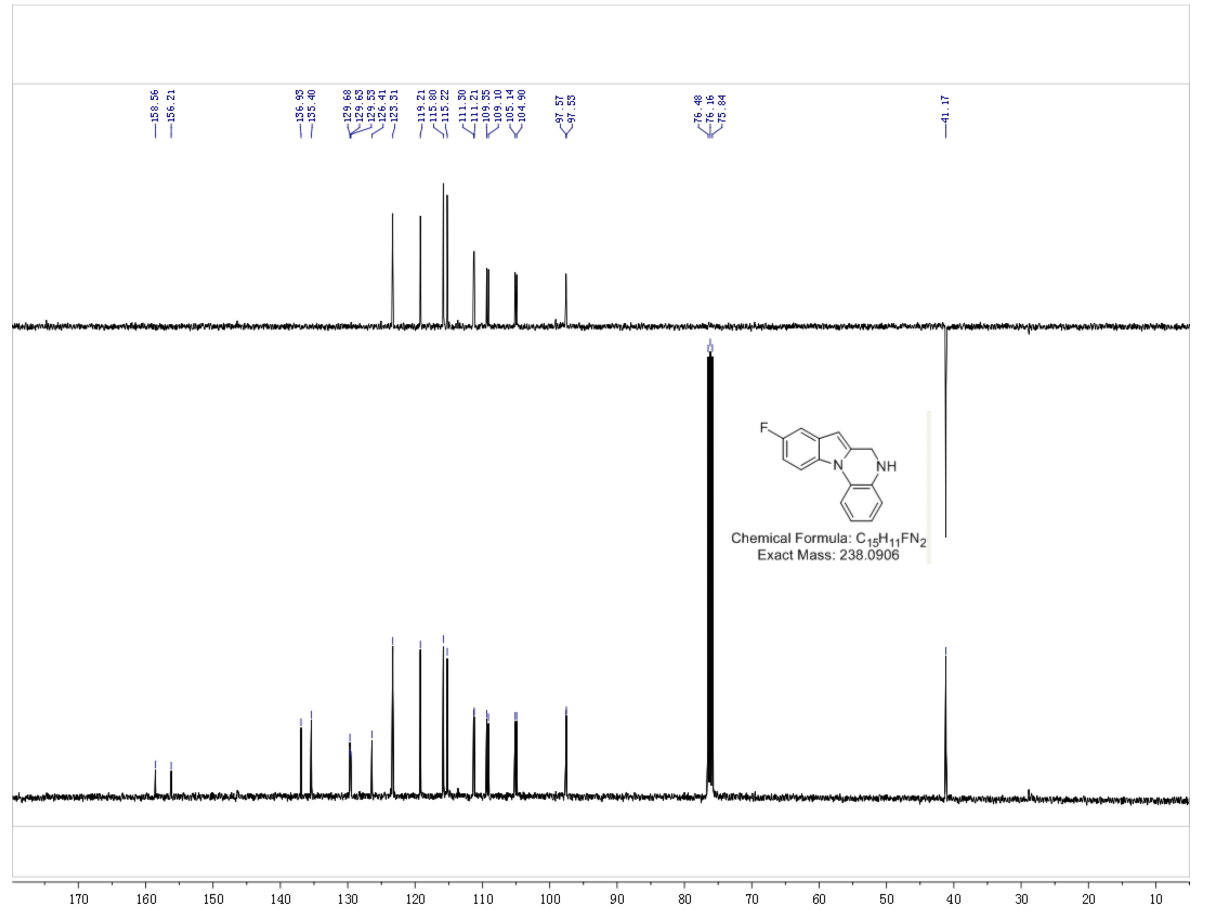

***N*-(5-chloro-1*H*-indol-2-ylmethyl)-2-iodoaniline (1c)**

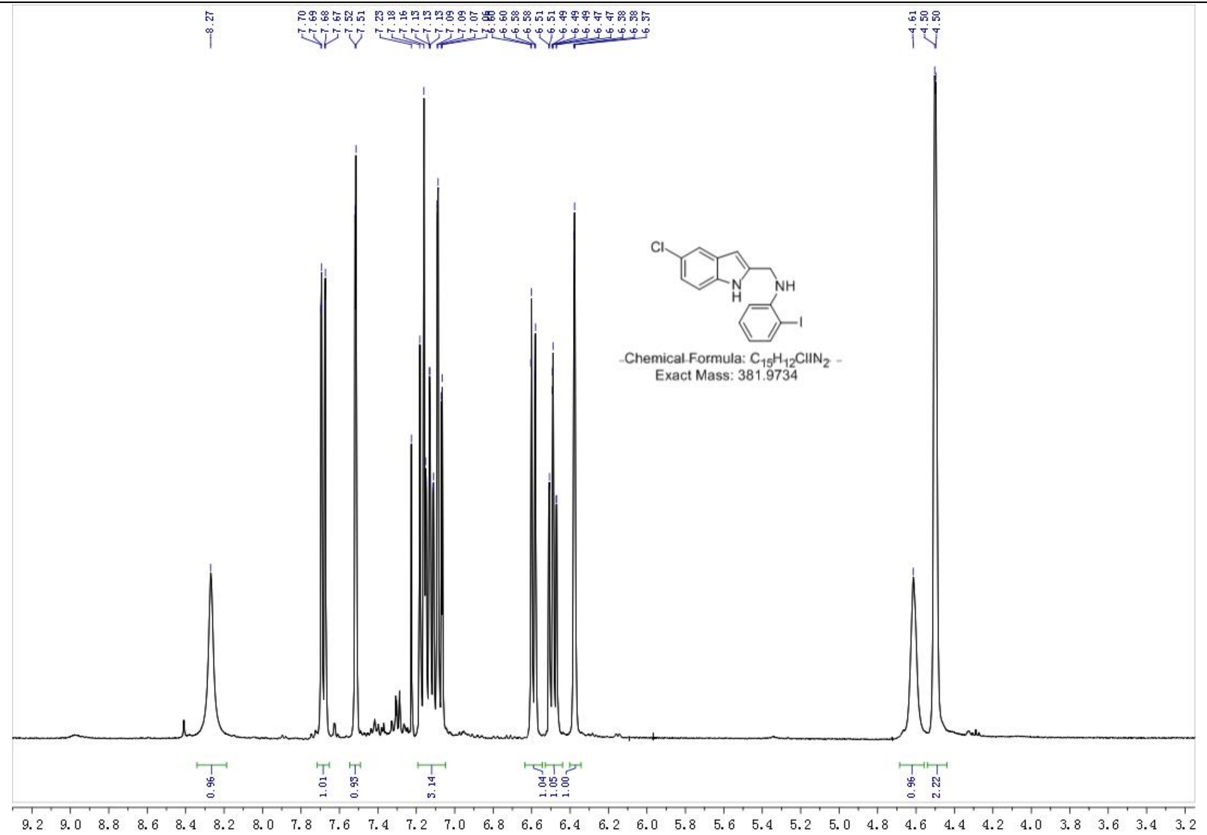

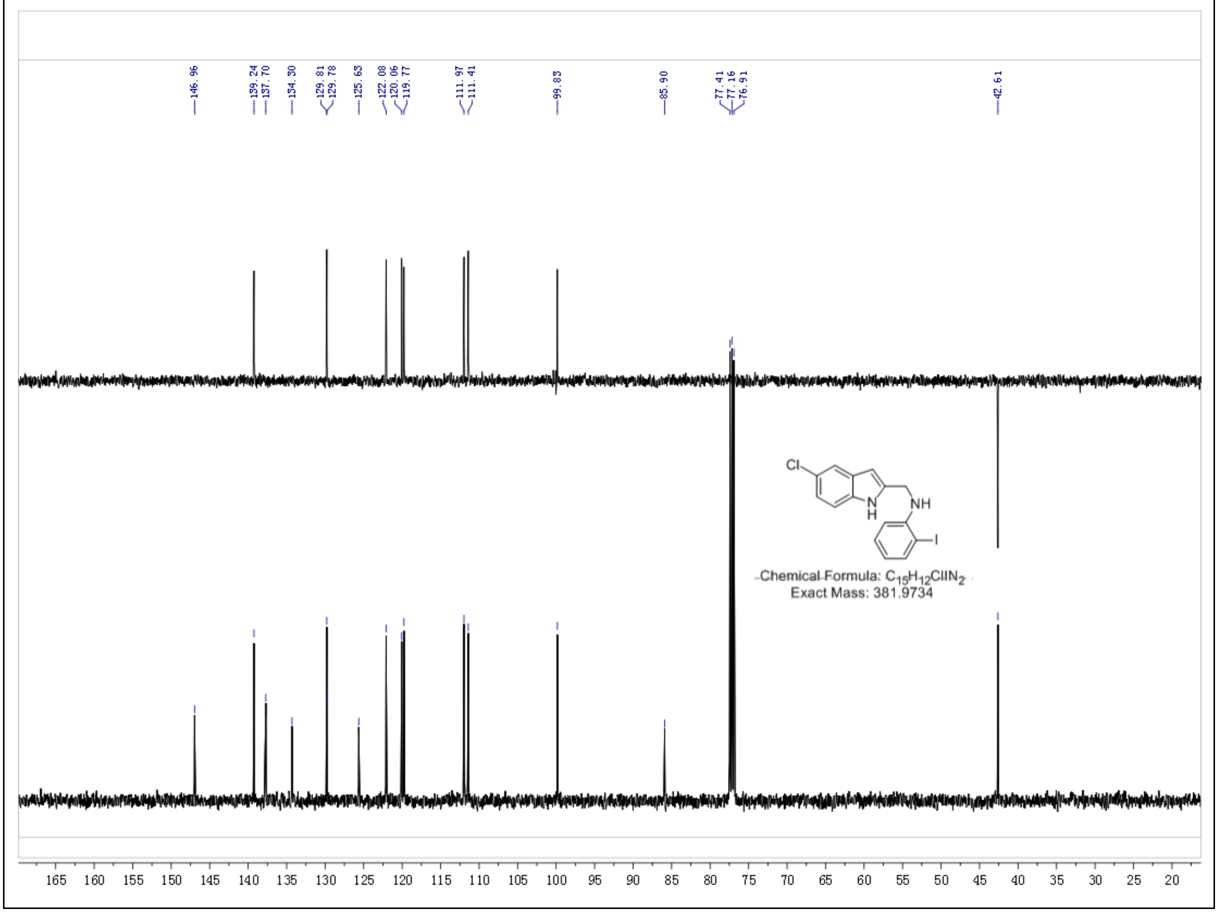

9-chloro-5,6-dihydroindolo[1,2-*a*]quinoxaline (2c)

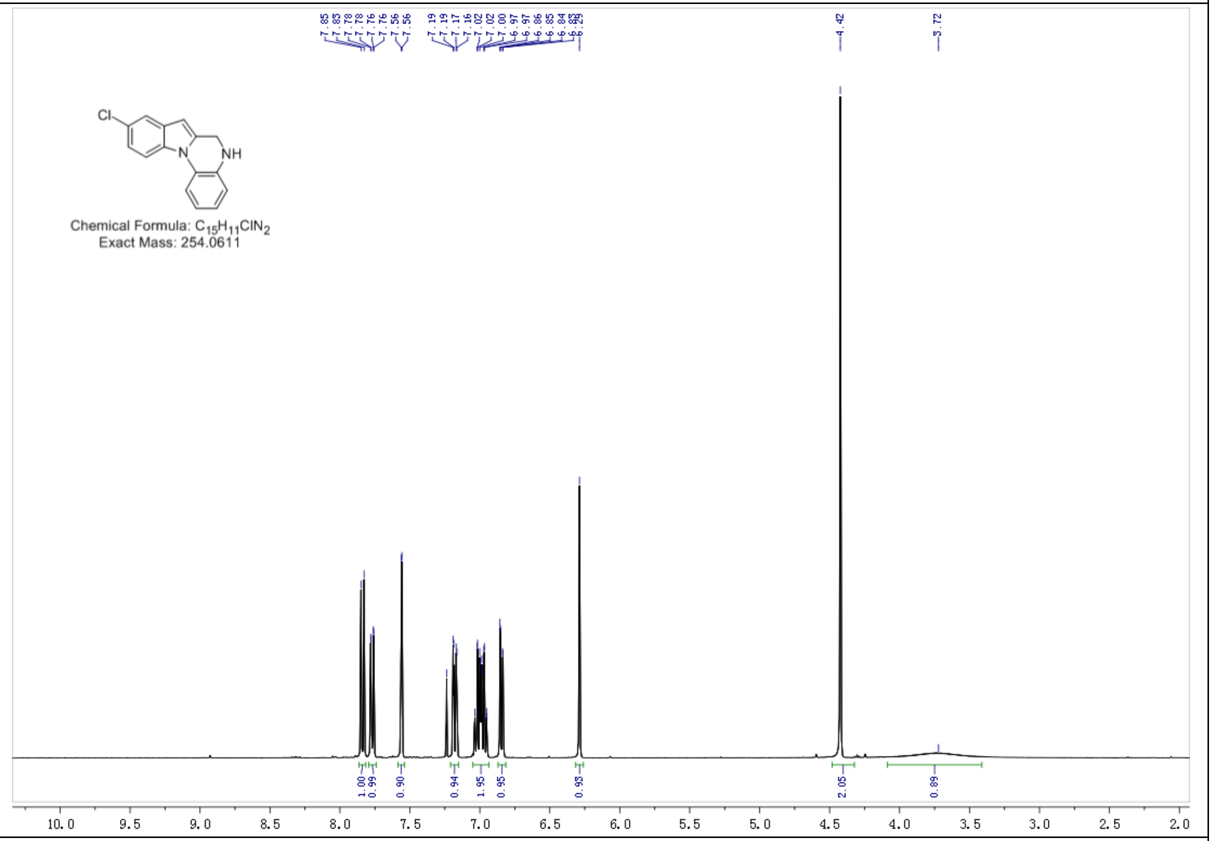

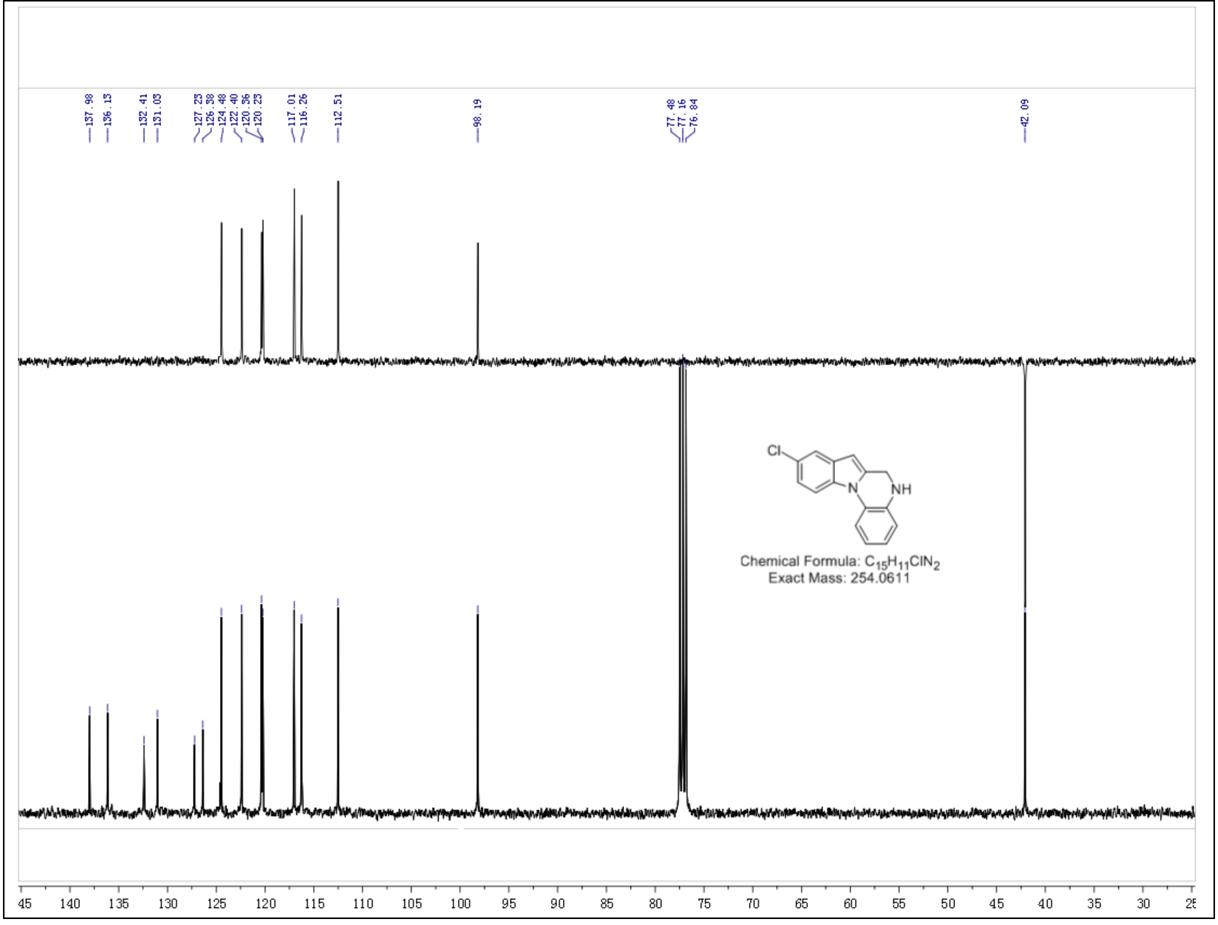

***N*-(5-bromo-1*H*-indol-2-ylmethyl)-2-iodoaniline (1d)**

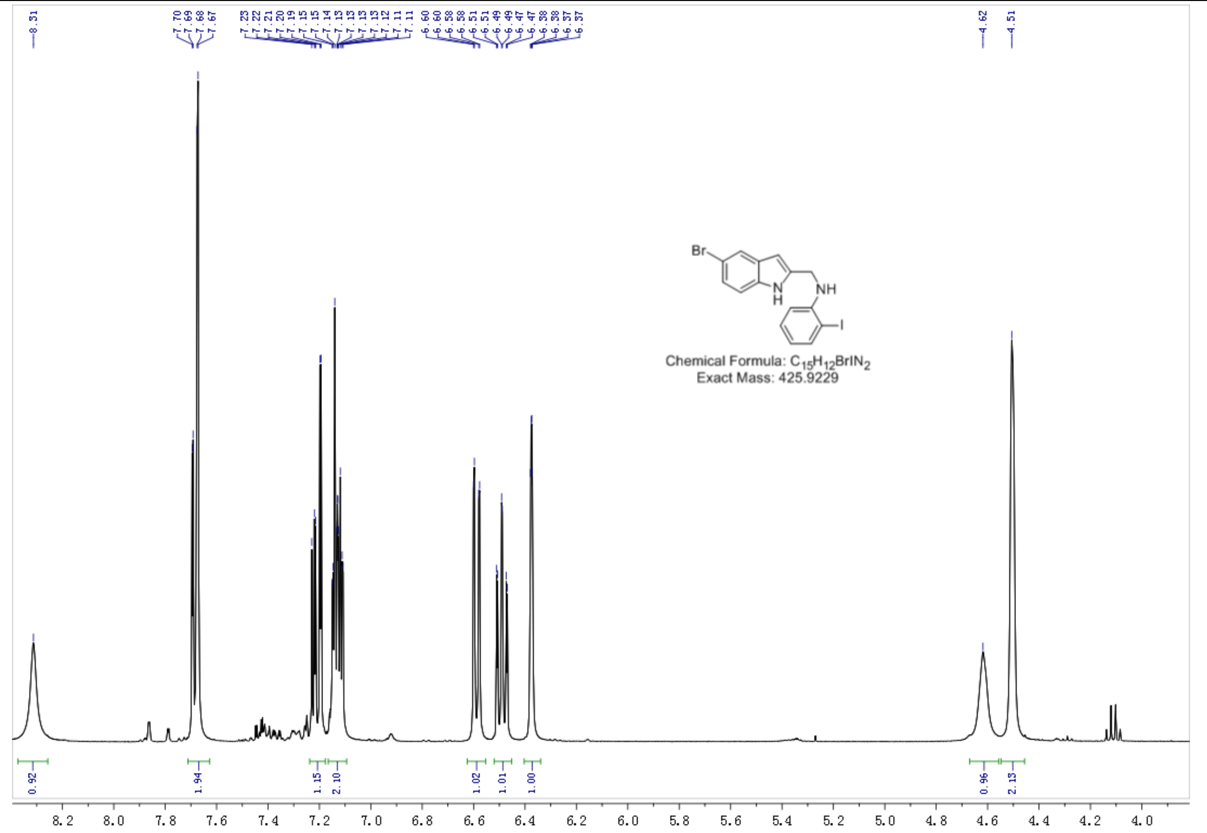

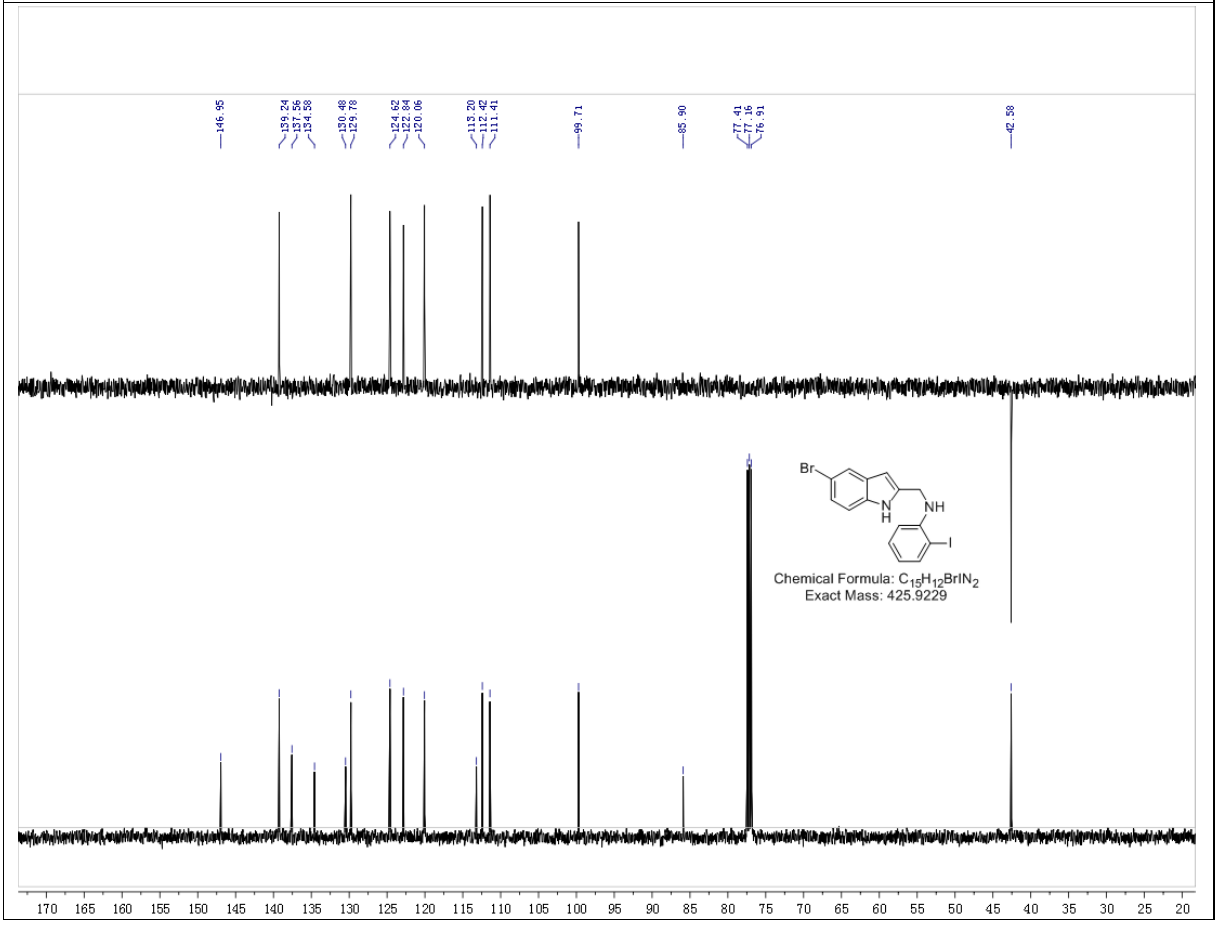

9-bromo-5,6-dihydroindolo[1,2-*a*]quinoxaline (2d)

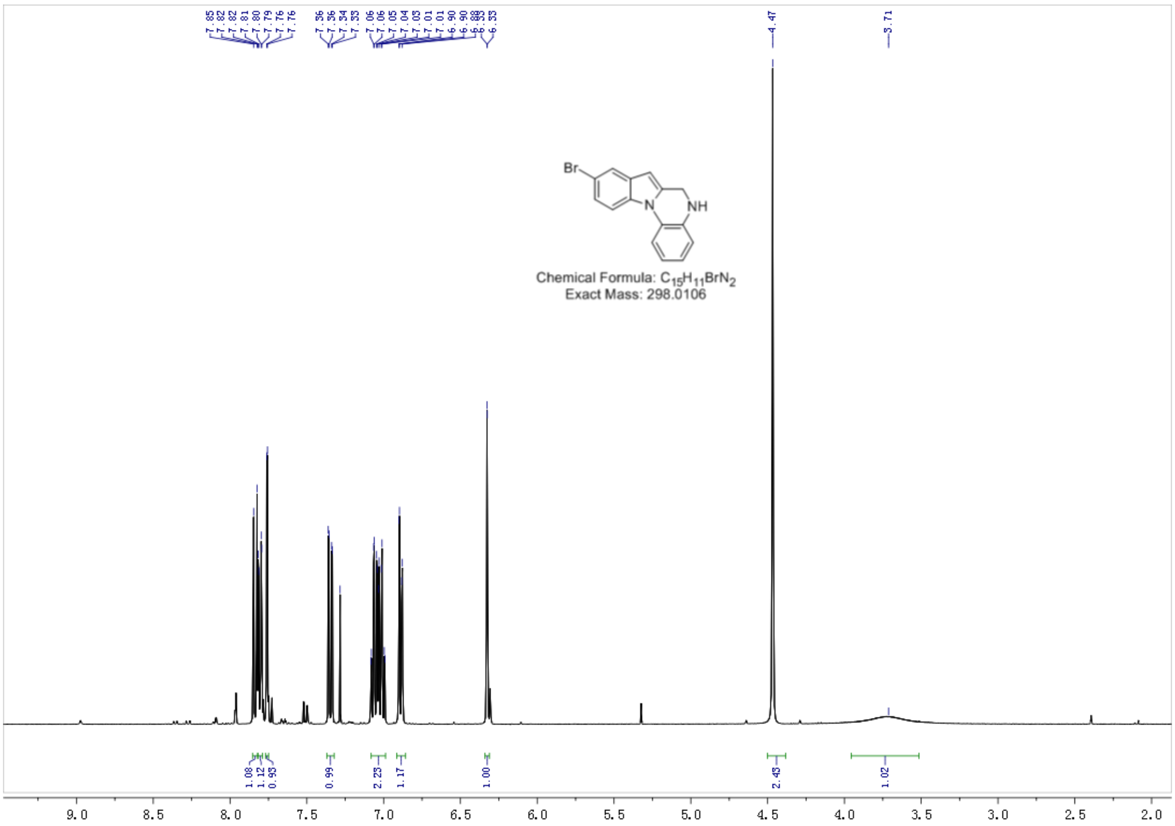

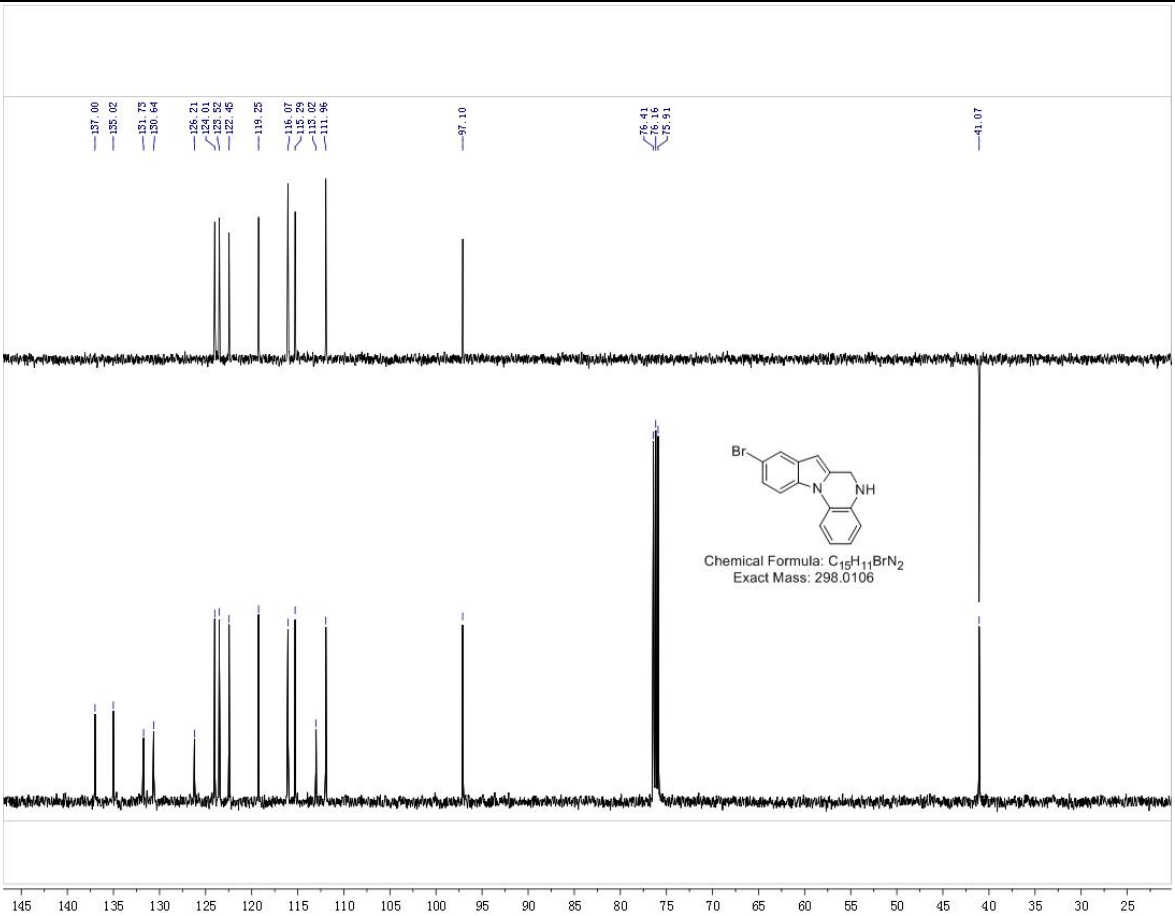

_N_-(5-nitro-1_H_-indol-2-ylmethyl)-2-iodoaniline (1e)

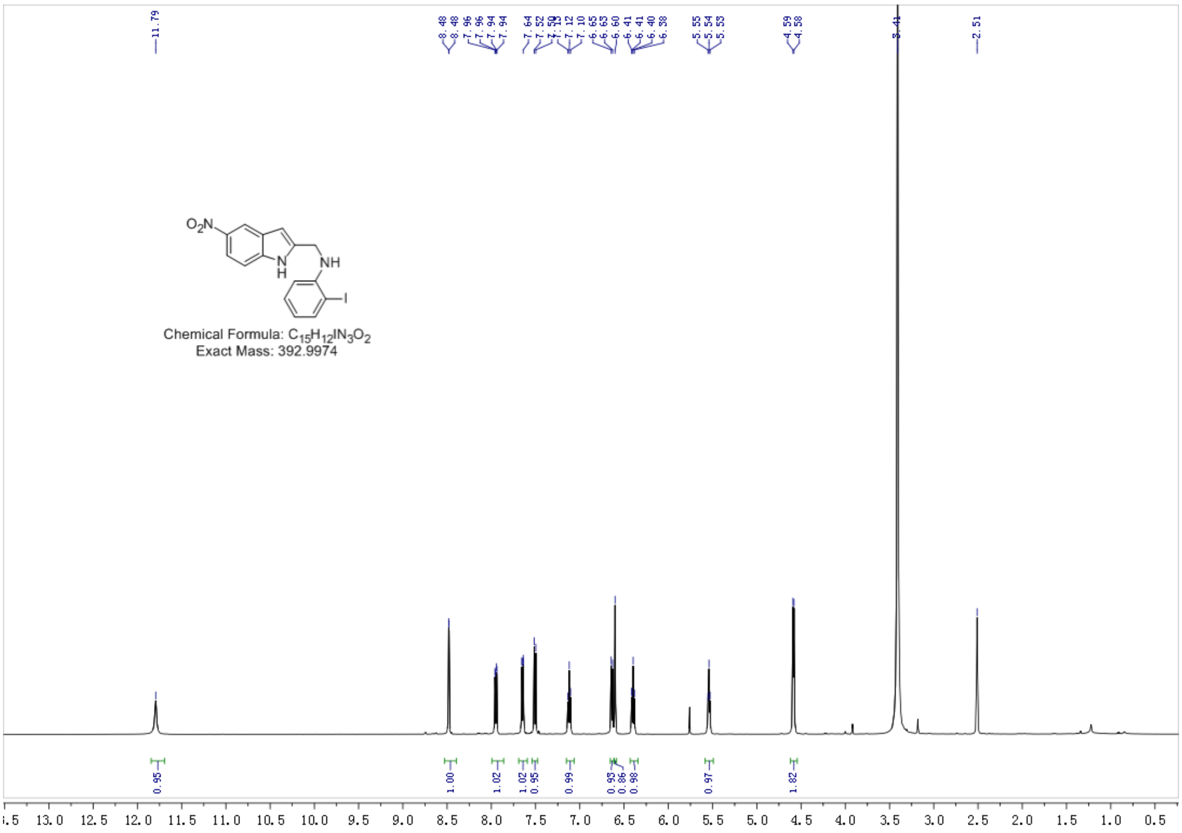

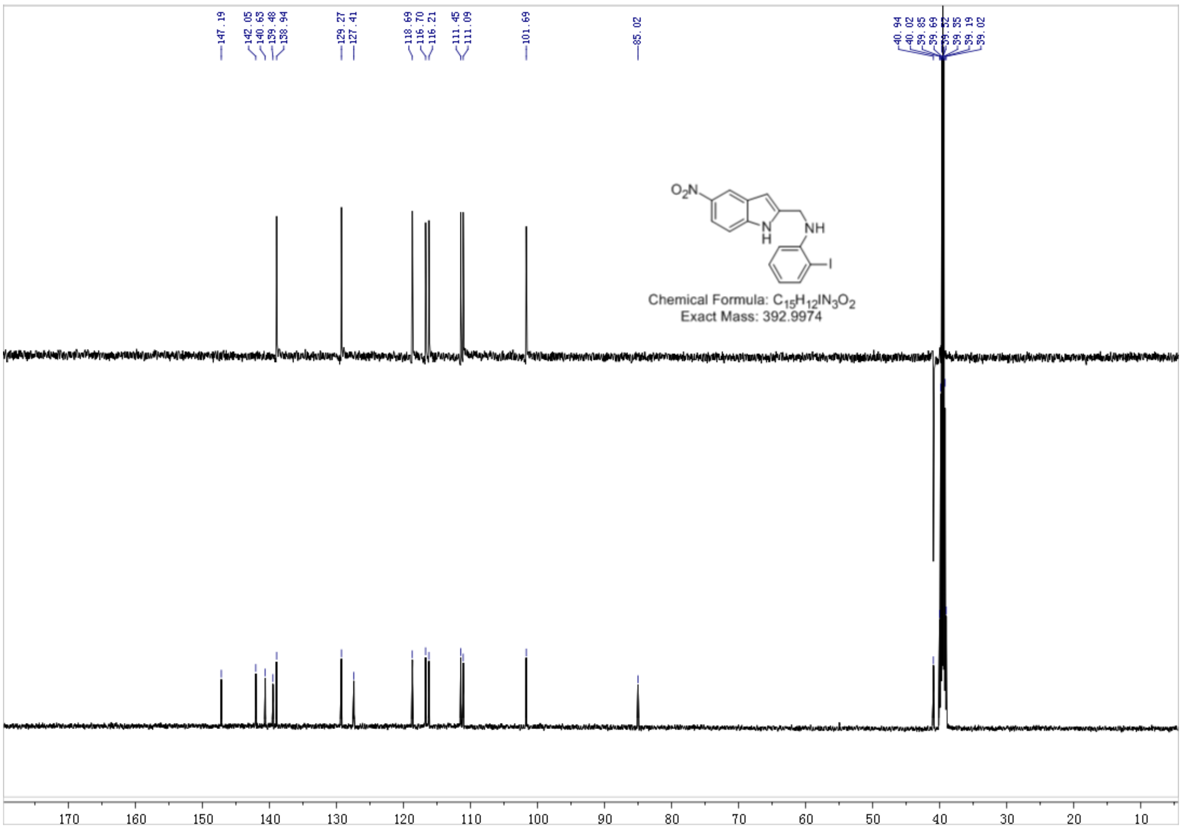

9-nitro-5,6-dihydroindolo[1,2-*a*]quinoxaline (2e)

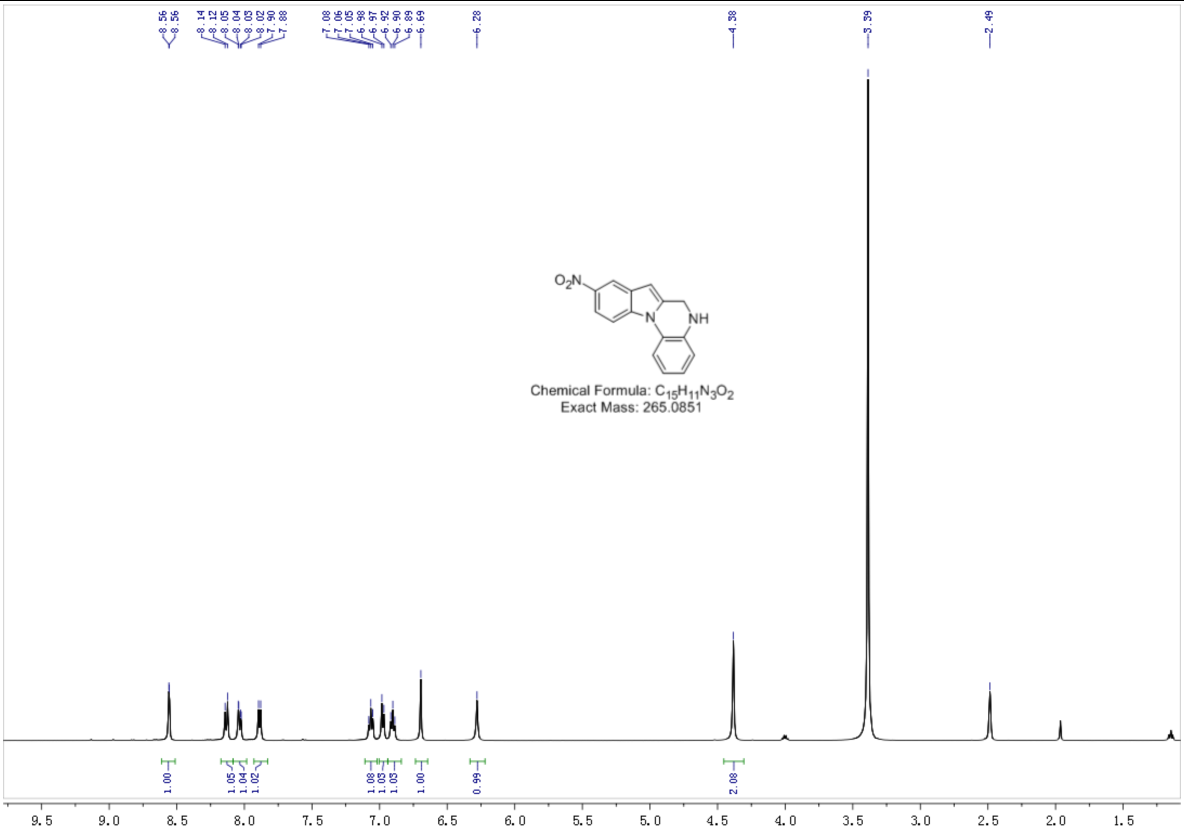

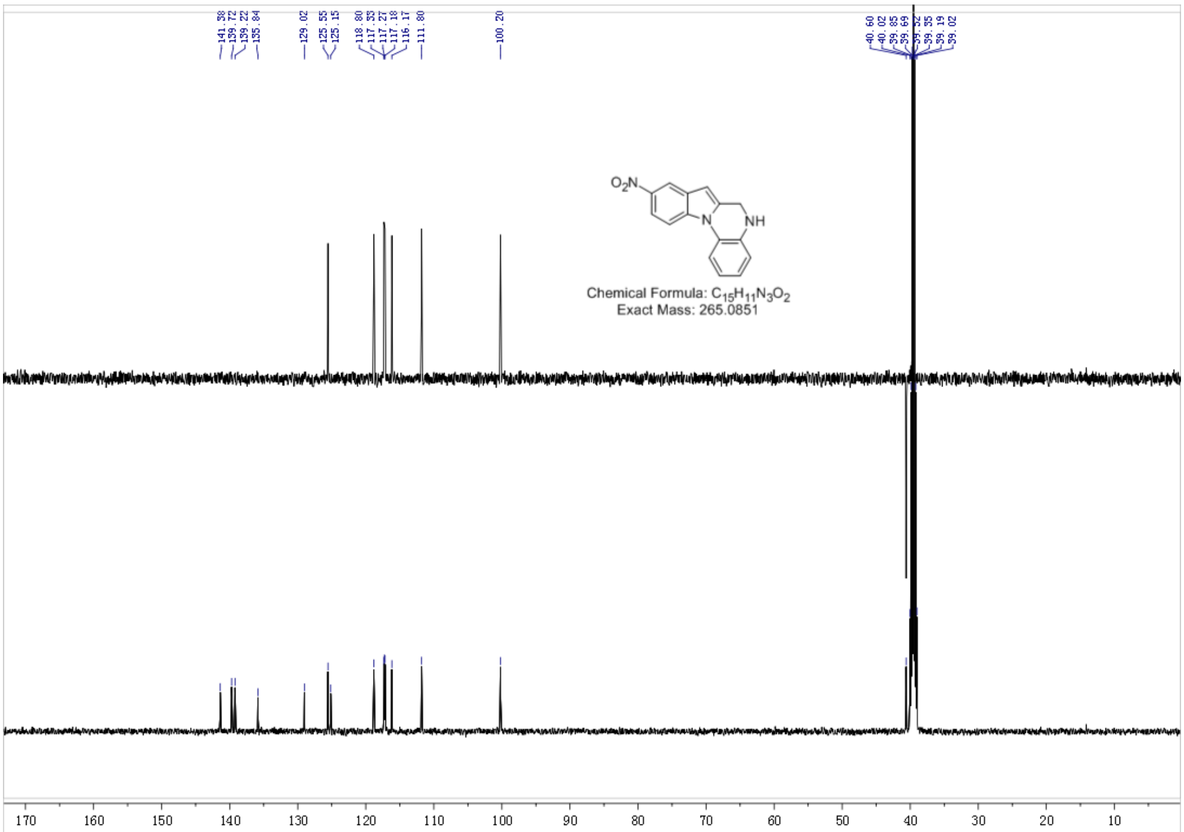

N-(5-methoxy-1H-indol-2-ylmethyl)-2-iodoaniline (1f)

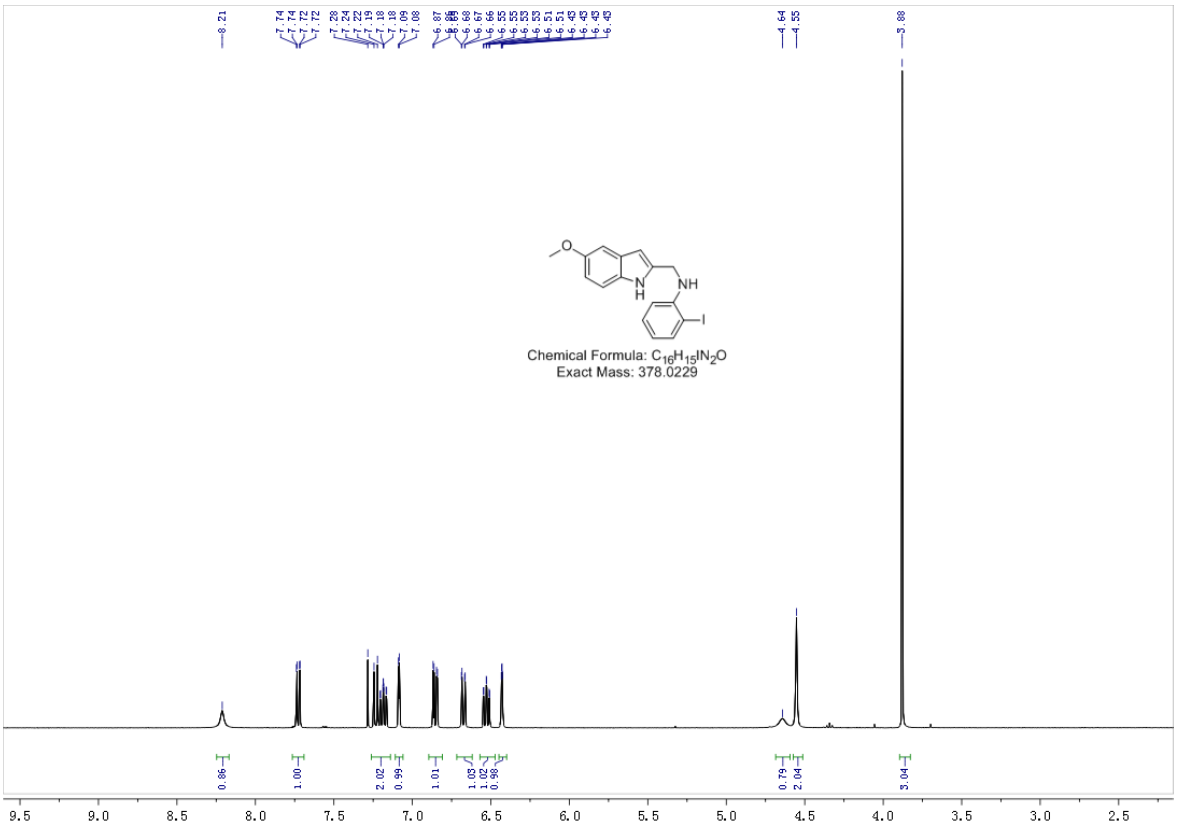

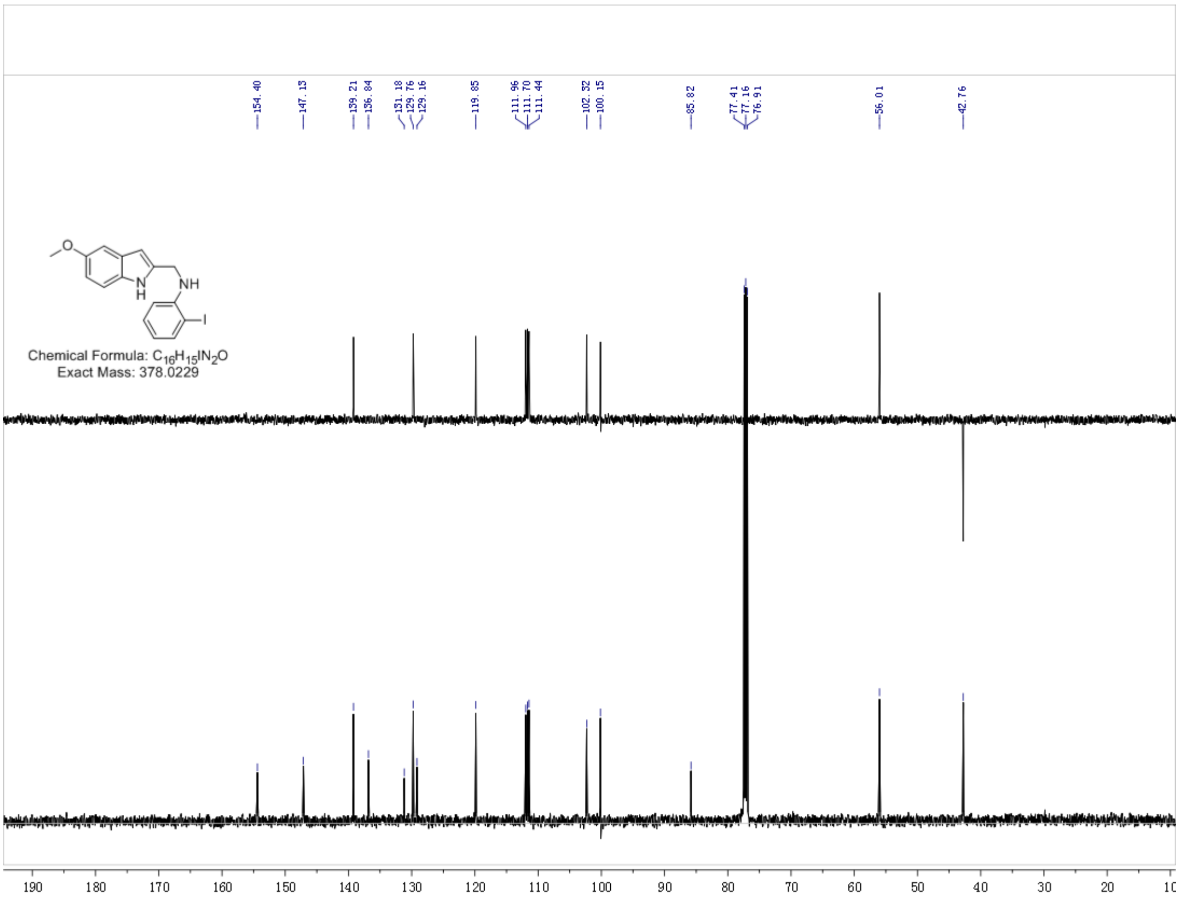

9-methoxy-5,6-dihydroindolo[1,2-*a*]quinoxaline (2f)

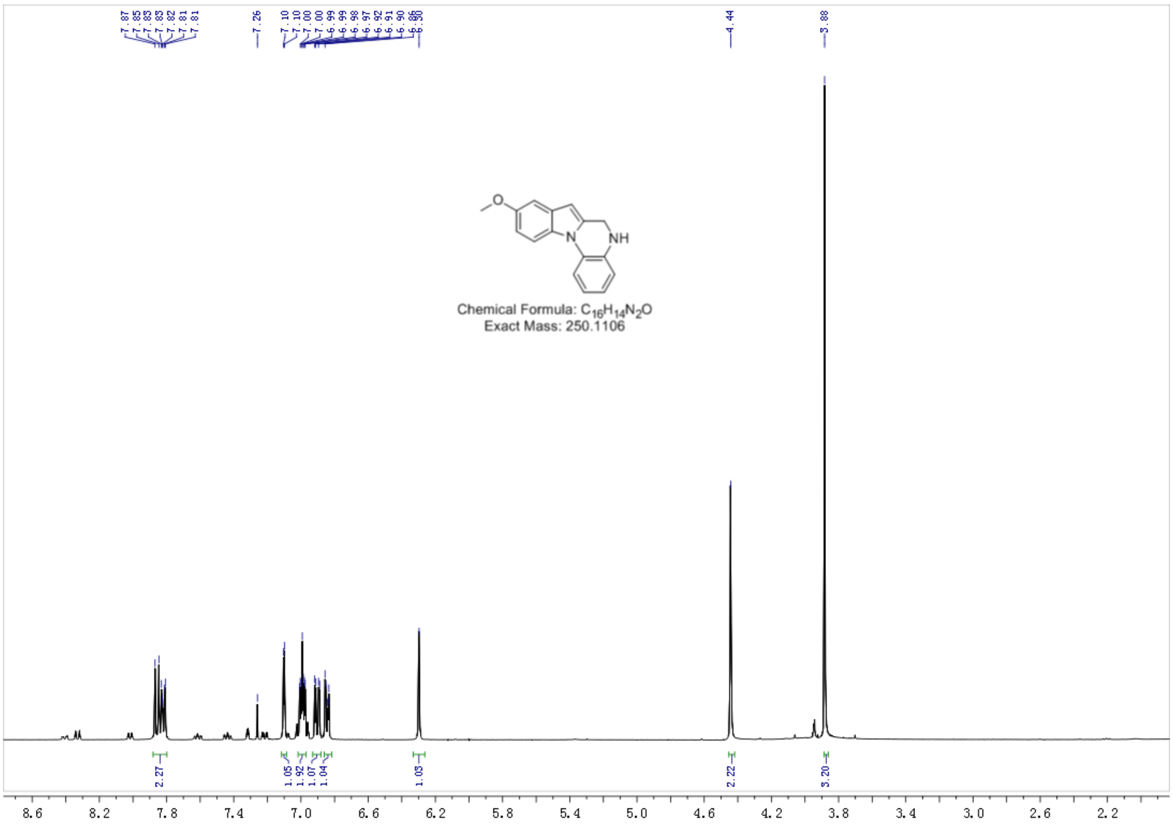

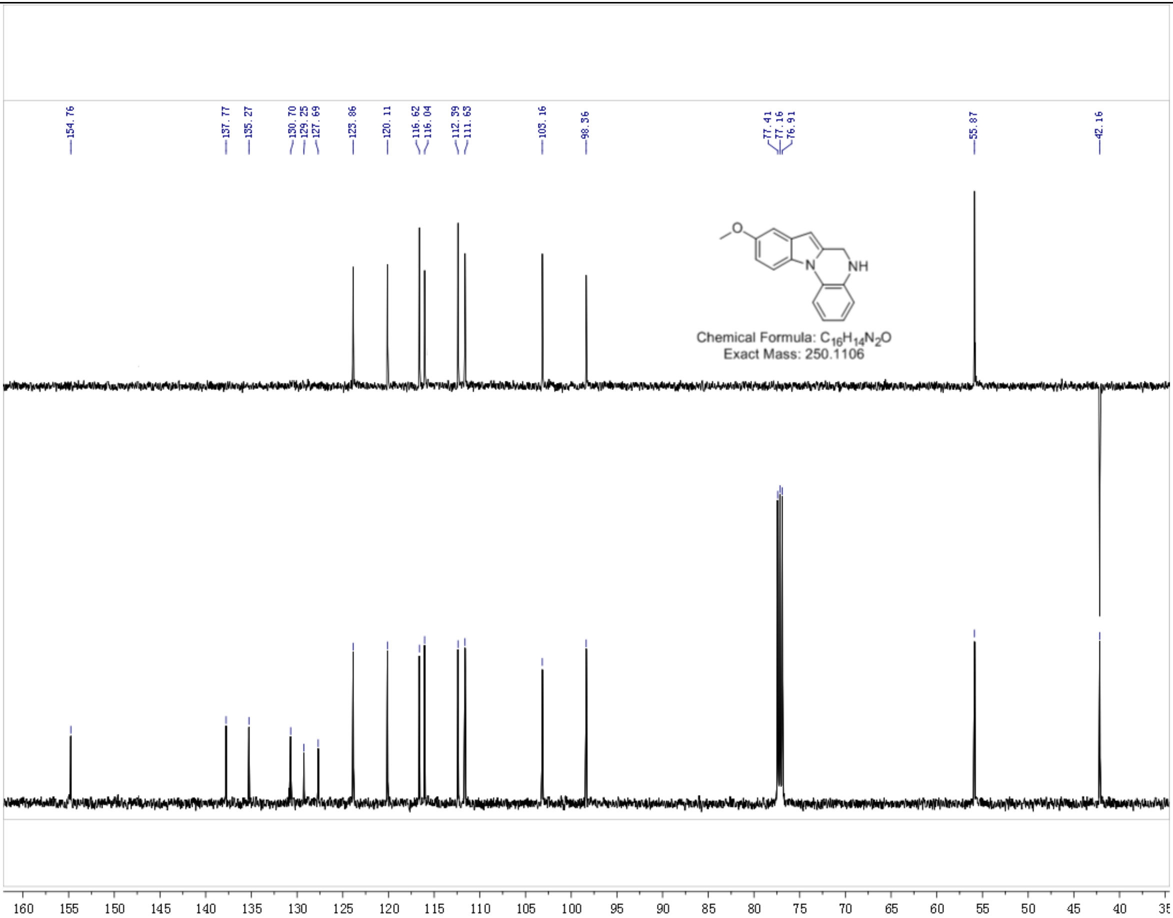

_N_-(5-methyl-1_H_-indol-2-ylmethyl)-2-iodoaniline (1g)

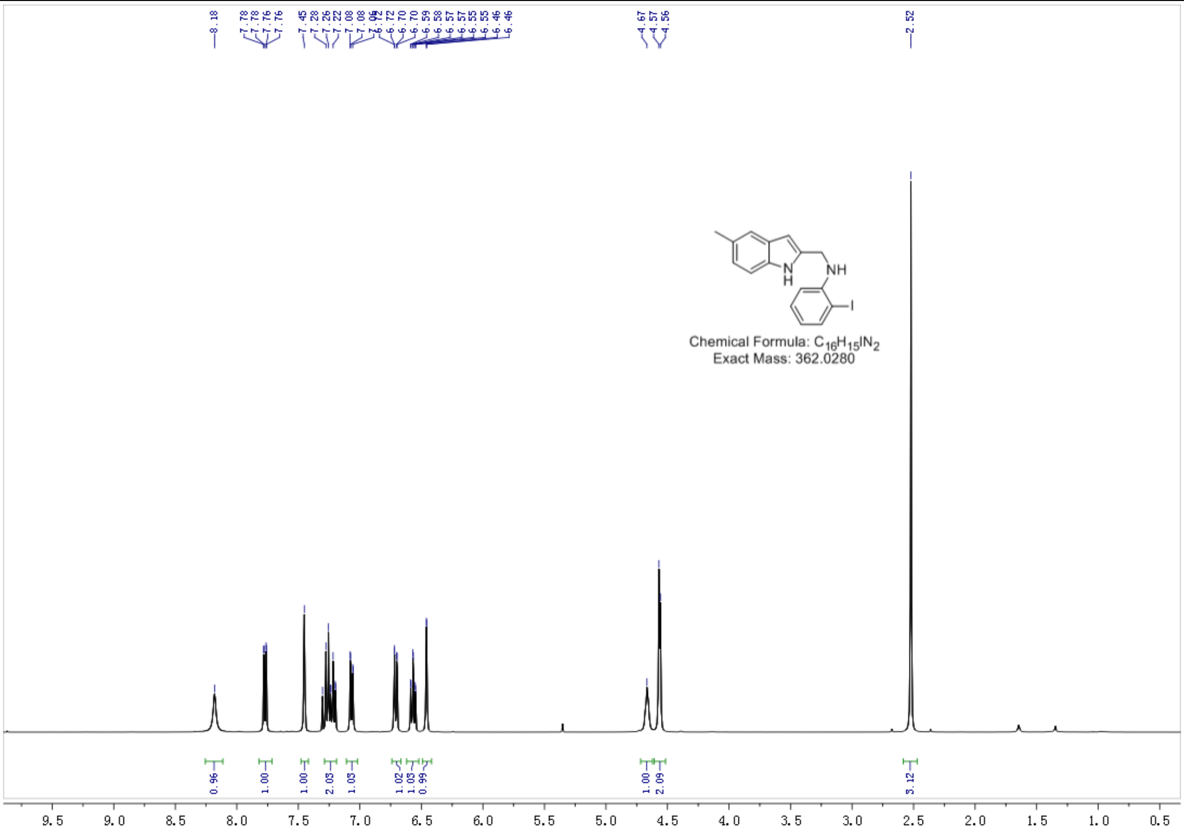

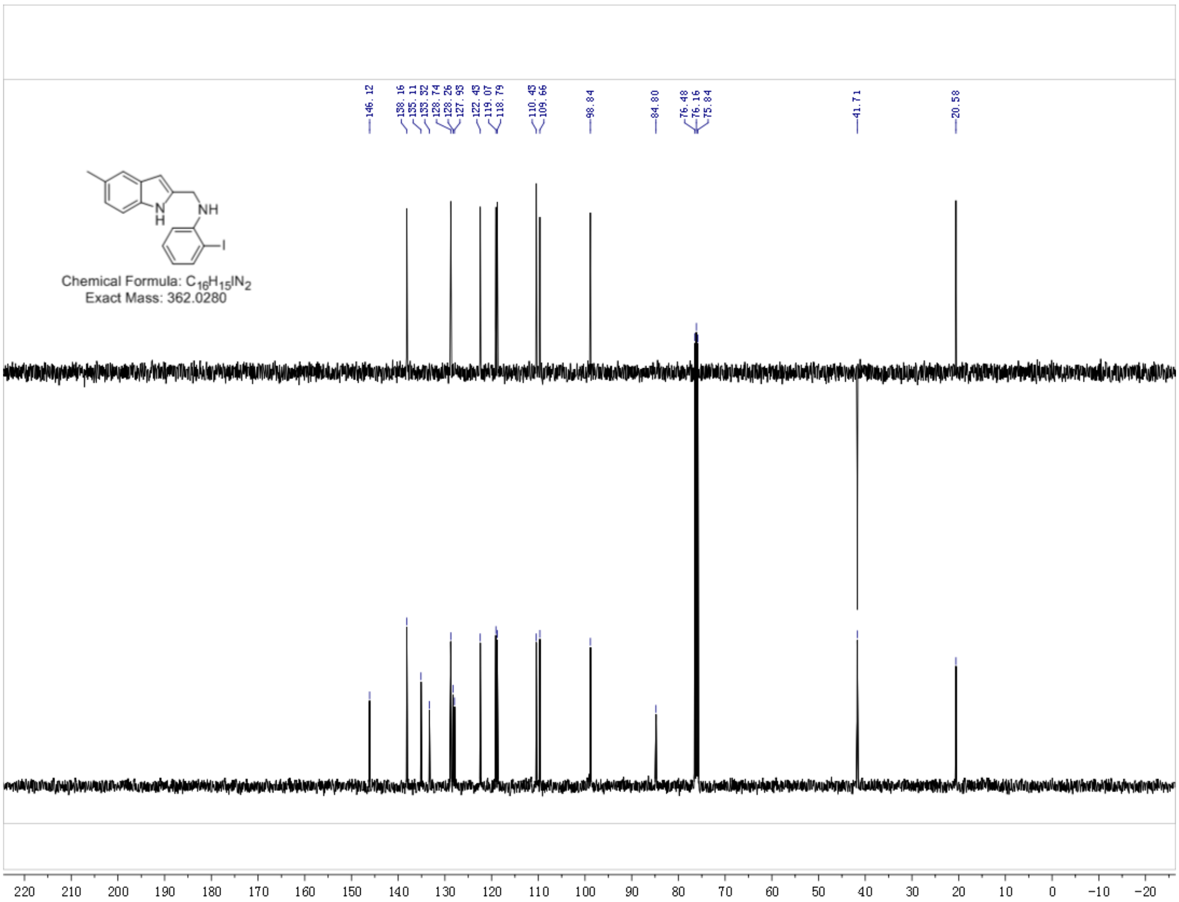

9-methyl-5,6-dihydroindolo[1,2-*a*]quinoxaline (2g)

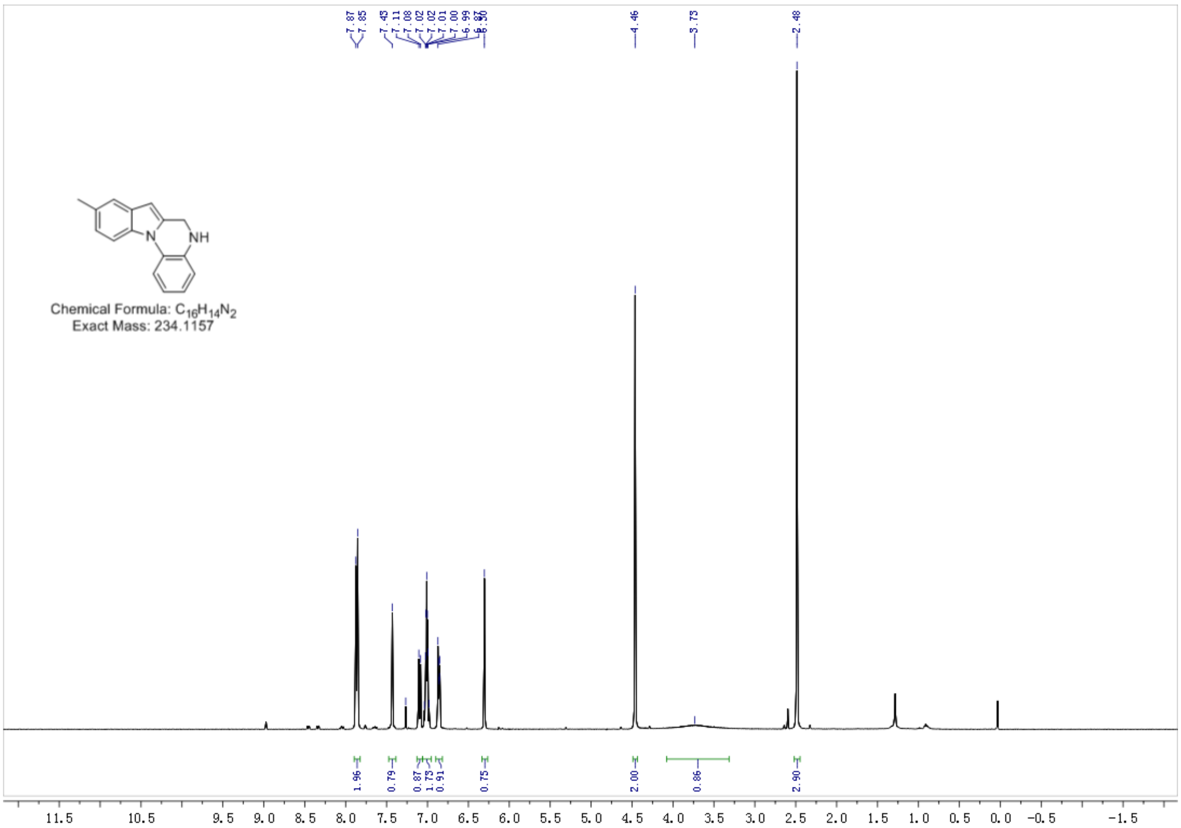

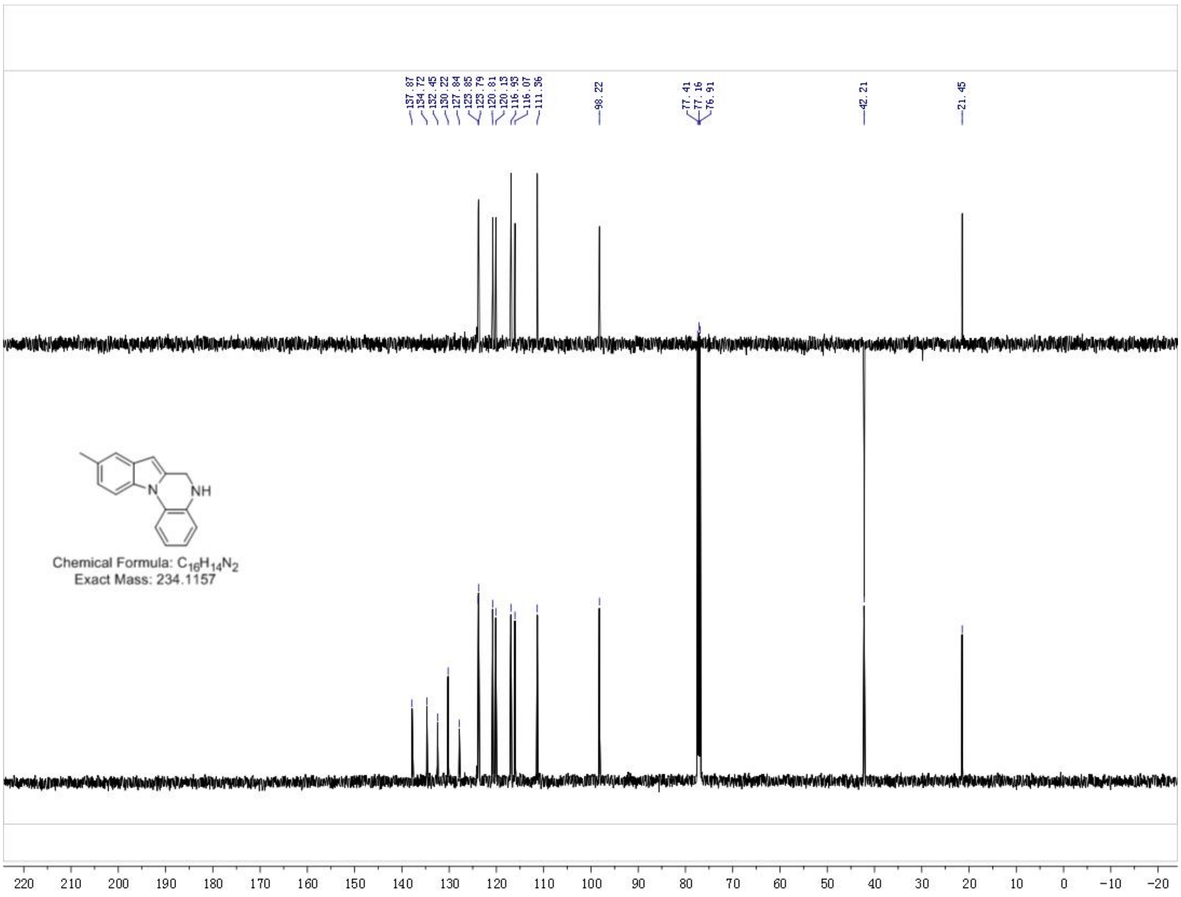

***N*-(5-methyl-1*H*-indol-2-ylmethyl)-5-chloro-2-iodoaniline (1h)**

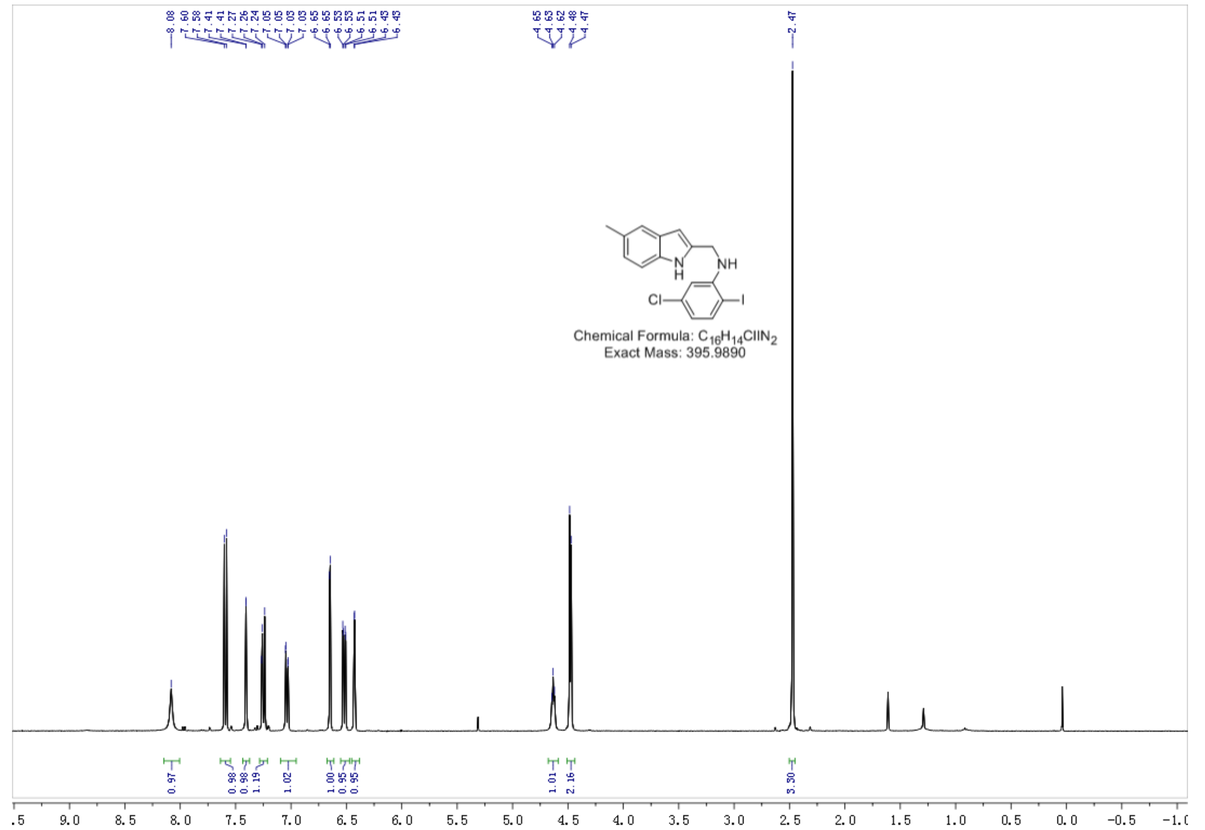

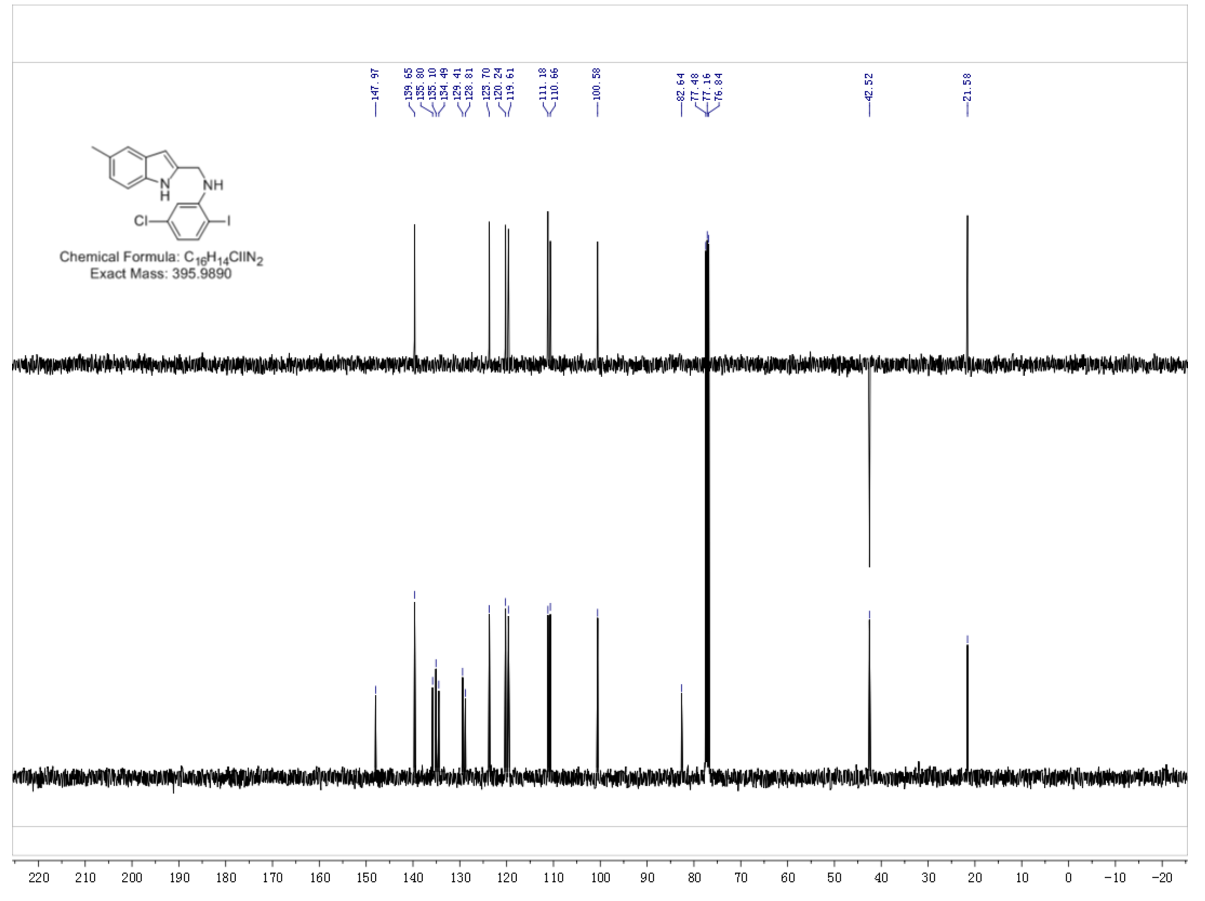

3-chloro-9-methyl-5,6-dihydroindolo[1,2-*a*]quinoxaline (2h)

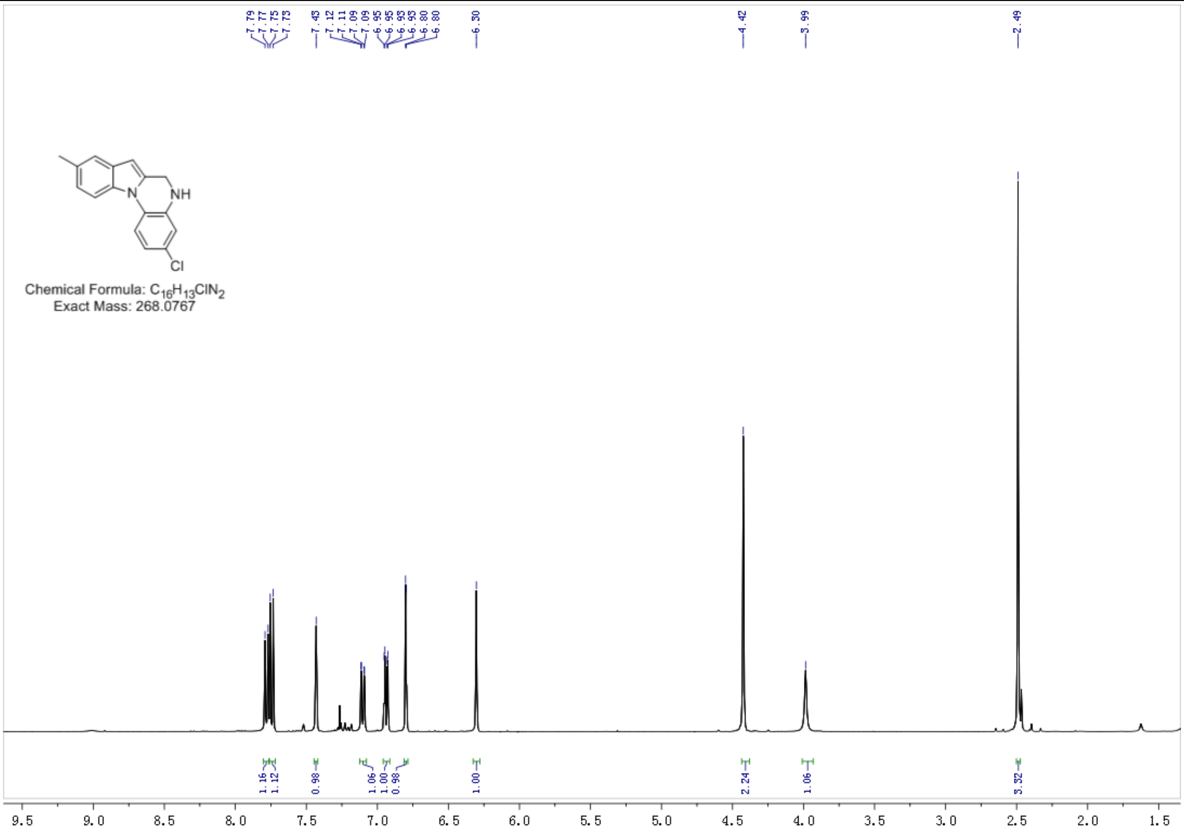

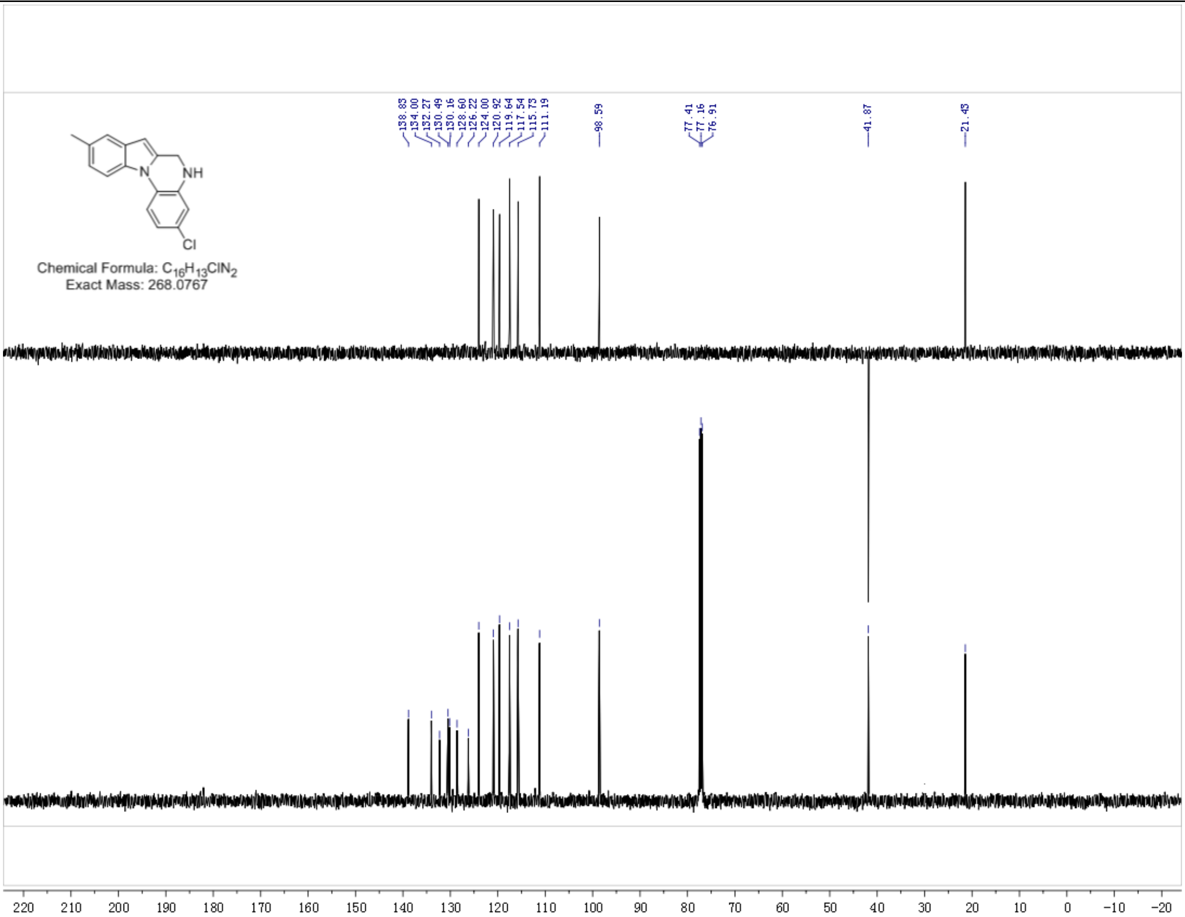

_N_-(1_H_-indol-2-ylmethyl)-4-trifluoromethyl-2-iodoaniline (1i)

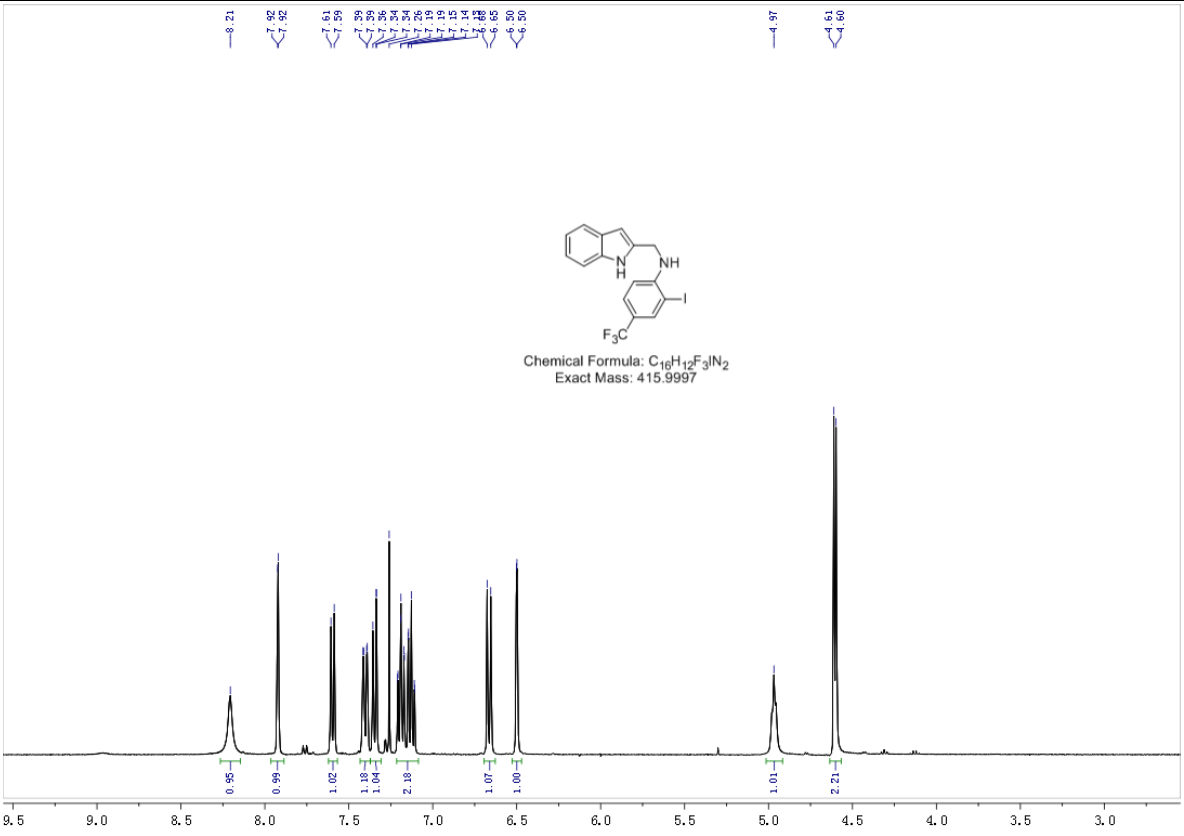

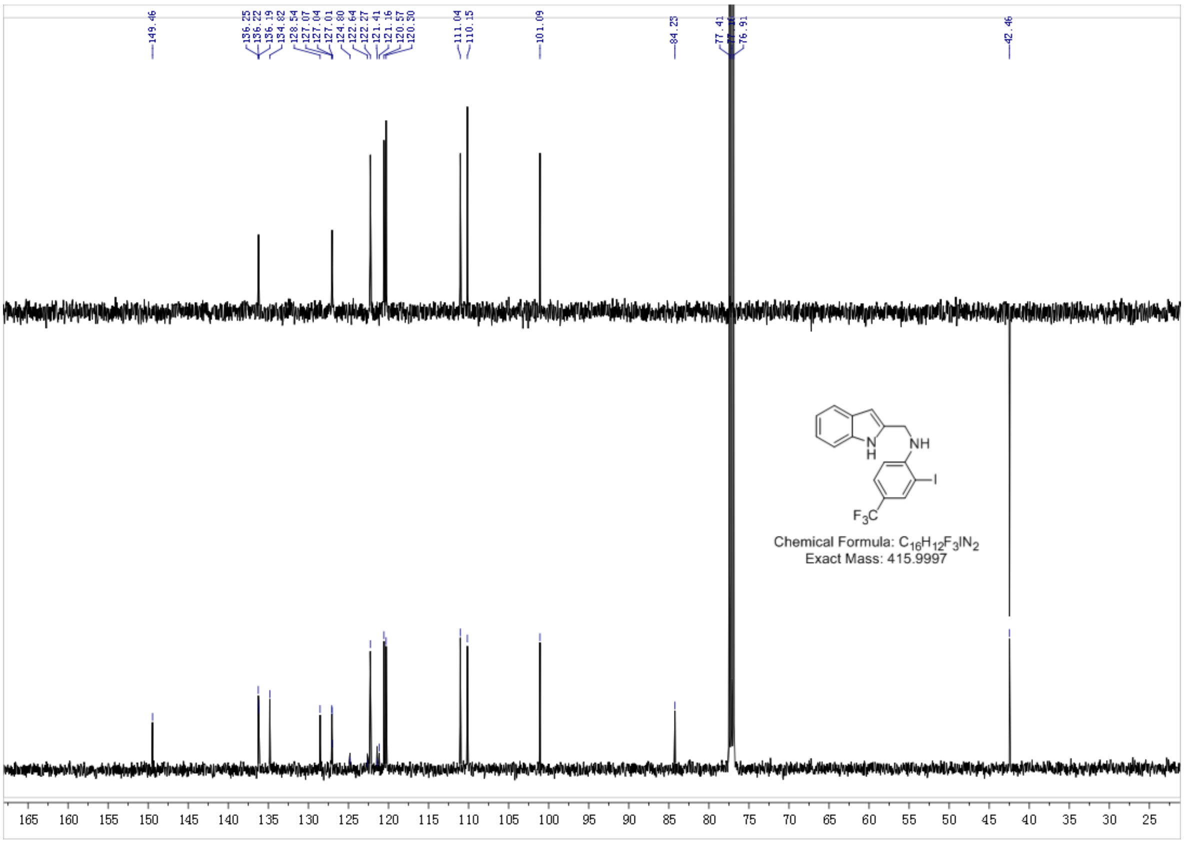

2-(trifluoromethyl)-5,6-dihydroindolo[1,2-*a*]quinoxaline (2i)

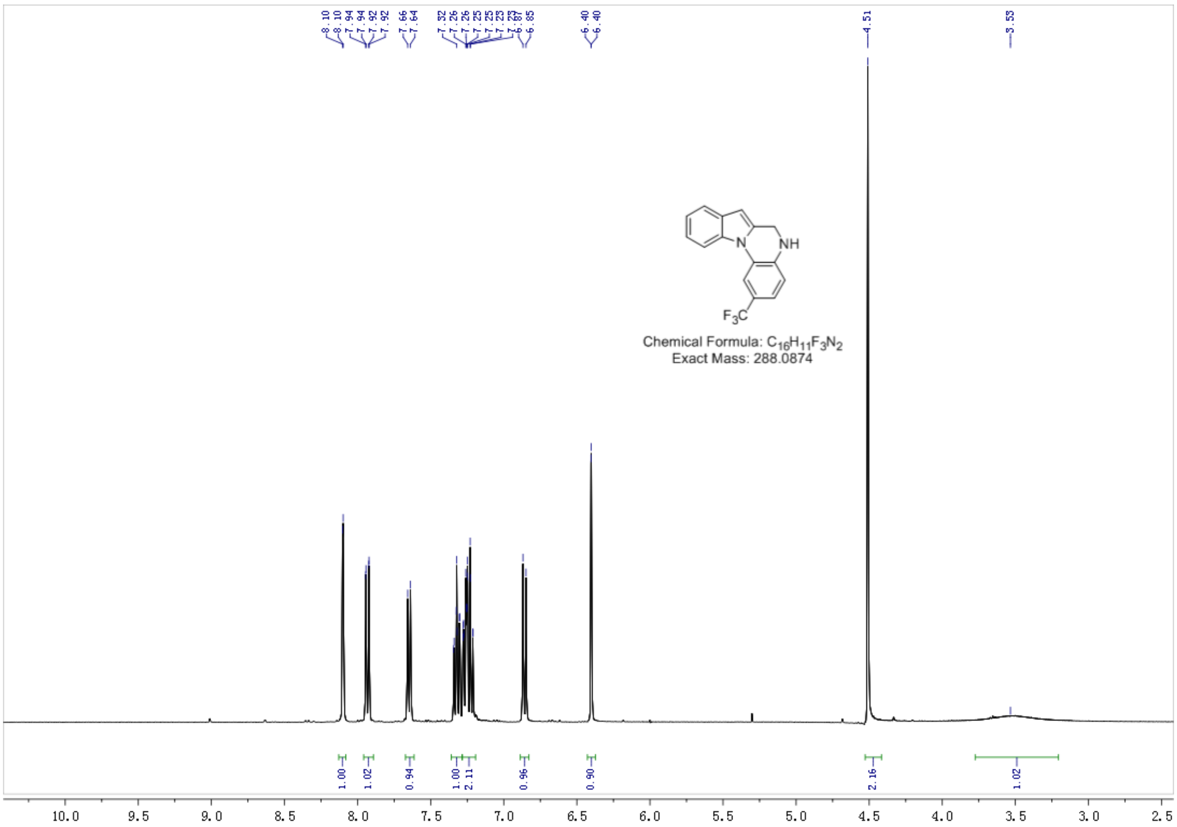

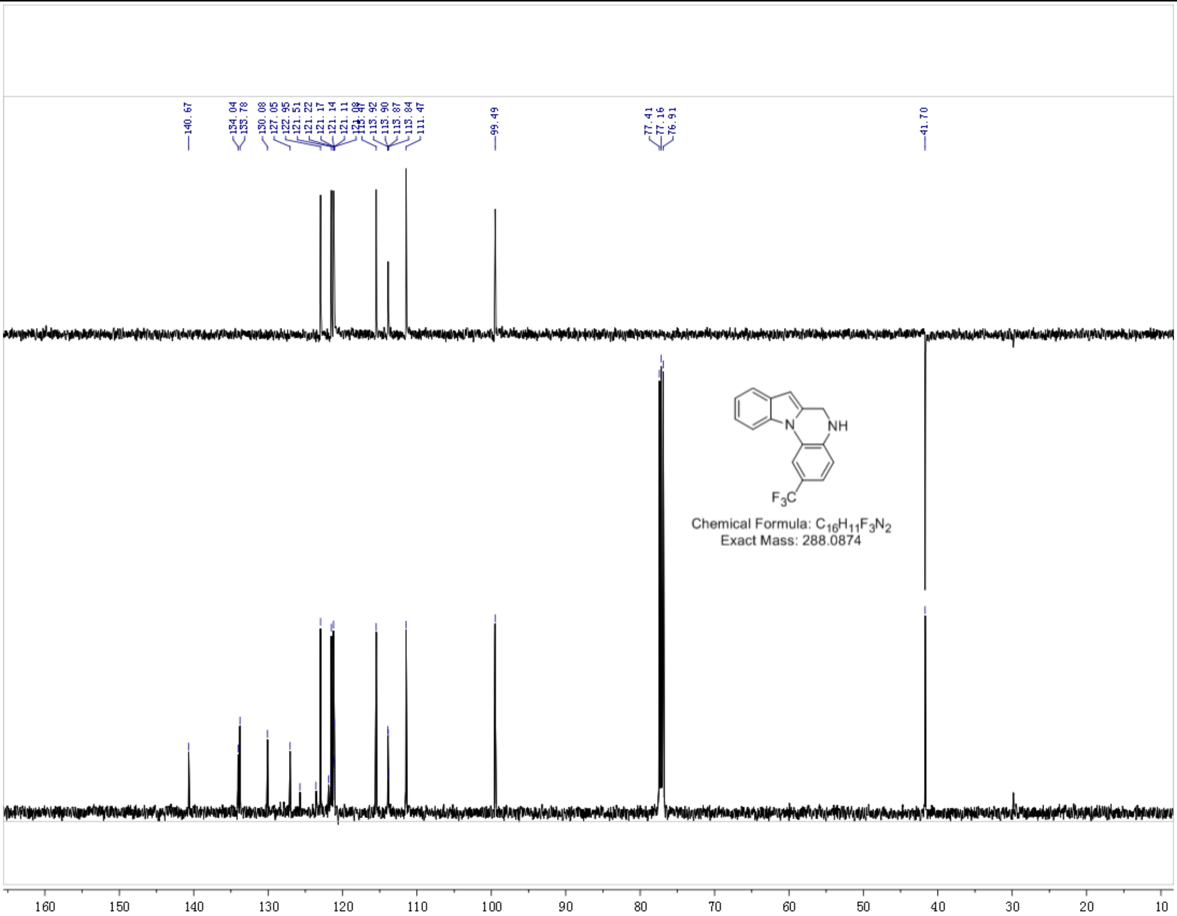

***N*-(1*H*-indol-2-ylmethyl)-5-chloro-2-iodoaniline (1j)**

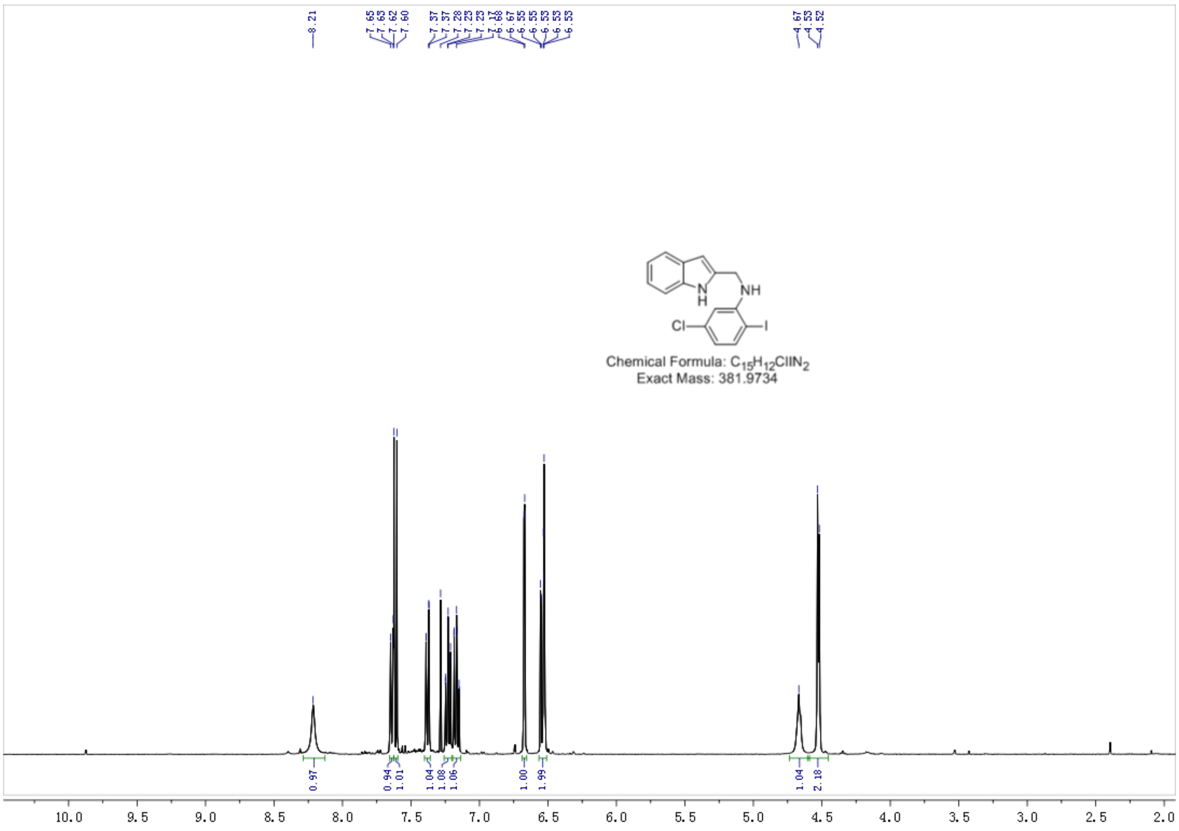

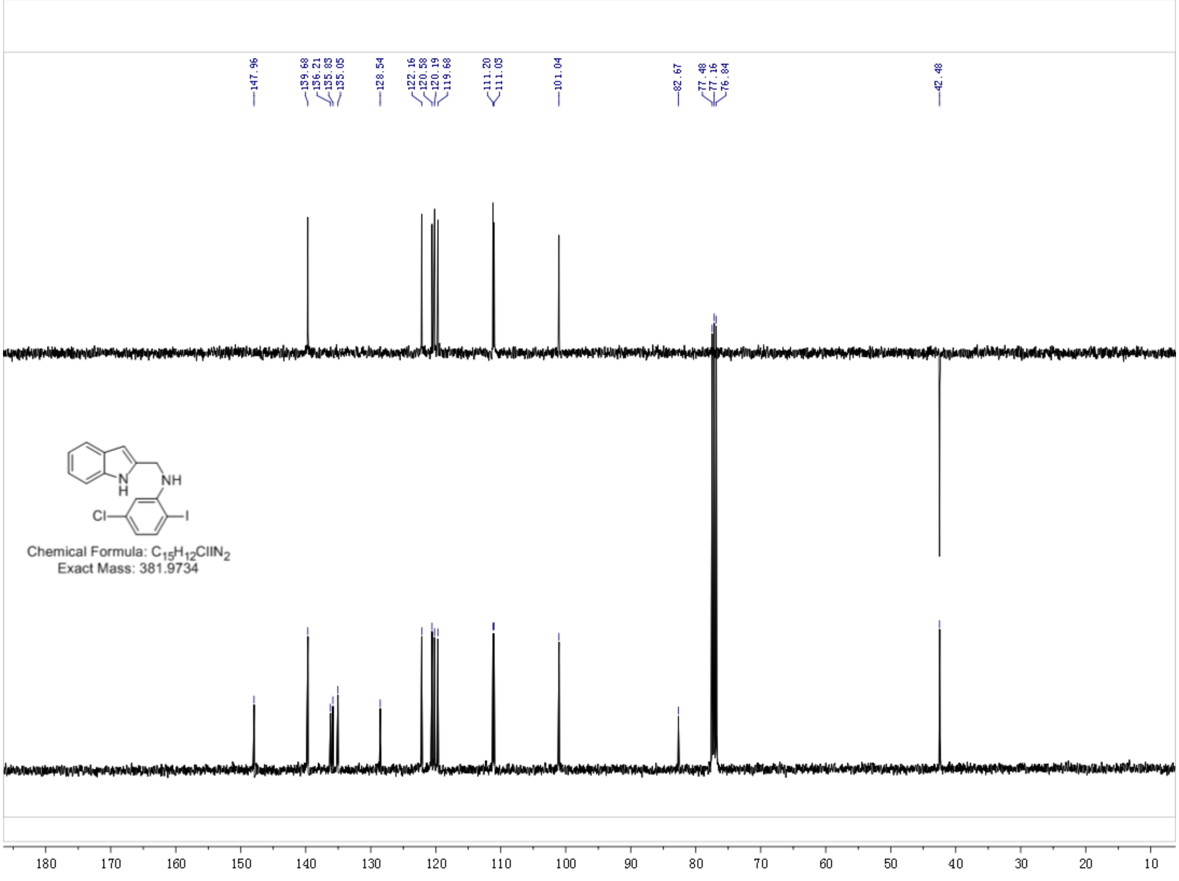

3-chloro-5,6-dihydroindolo[1,2-*a*]quinoxaline (2j)

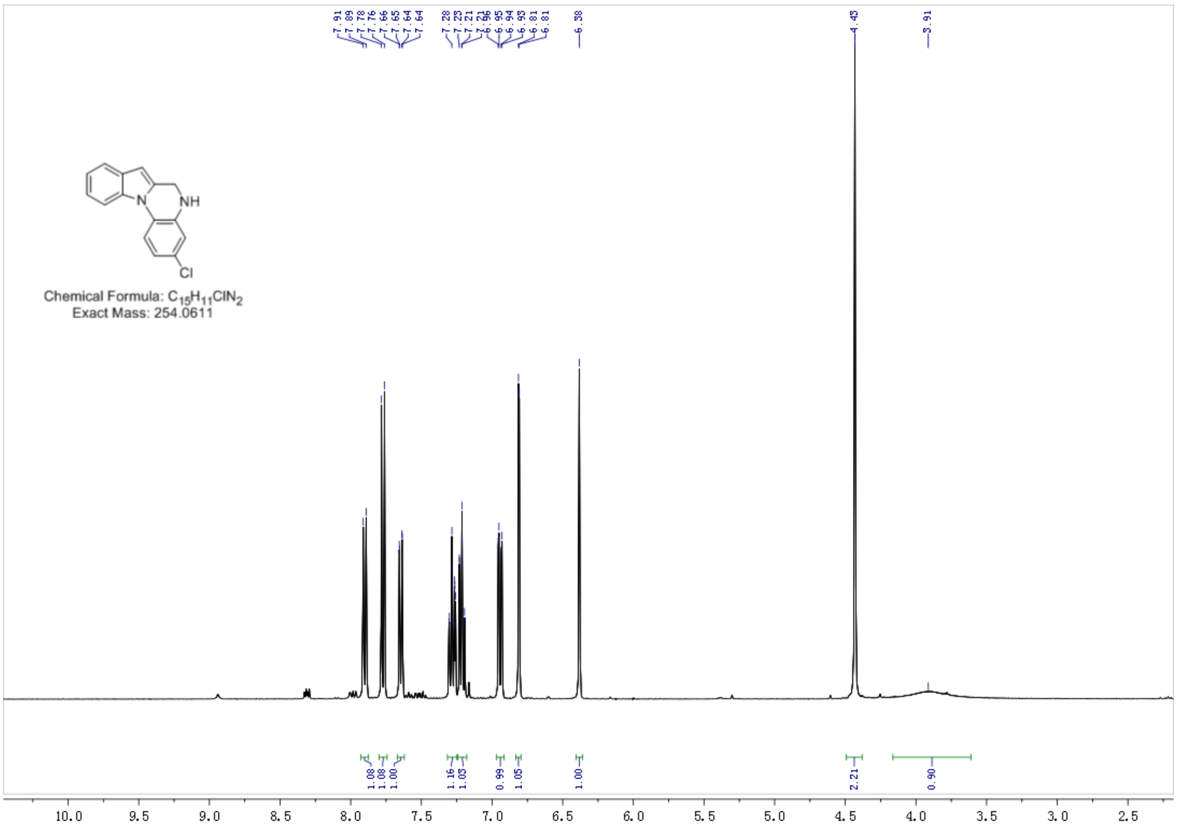

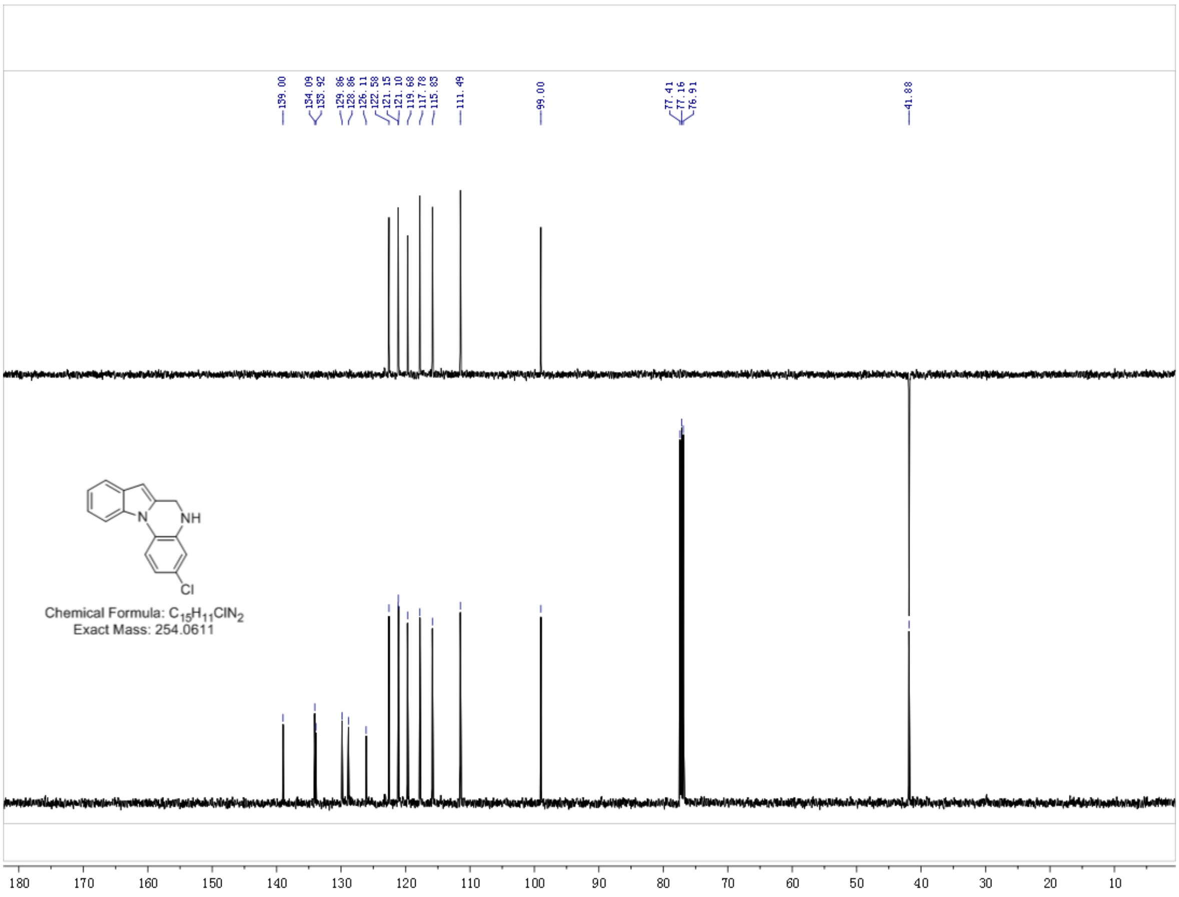

***N*-(1*H*-indol-2-ylmethyl)-5-fluoro-2-iodoaniline (1k)**

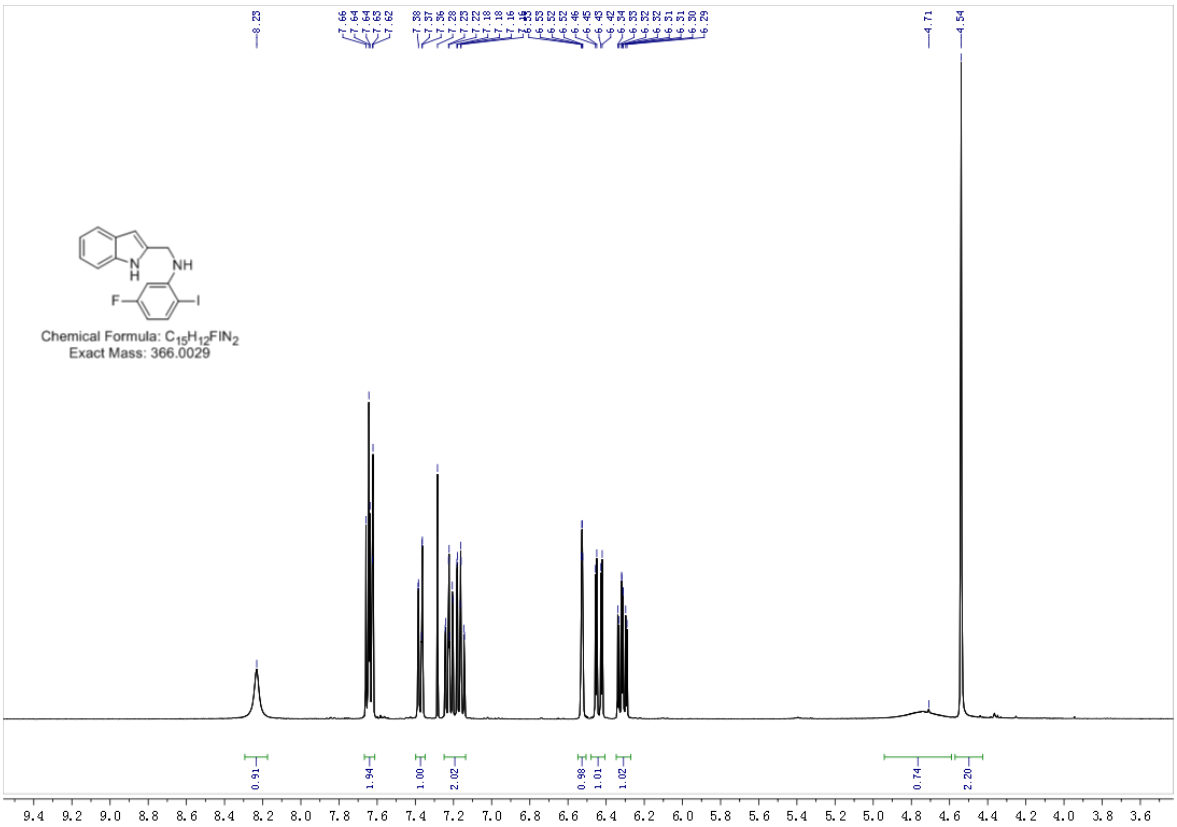

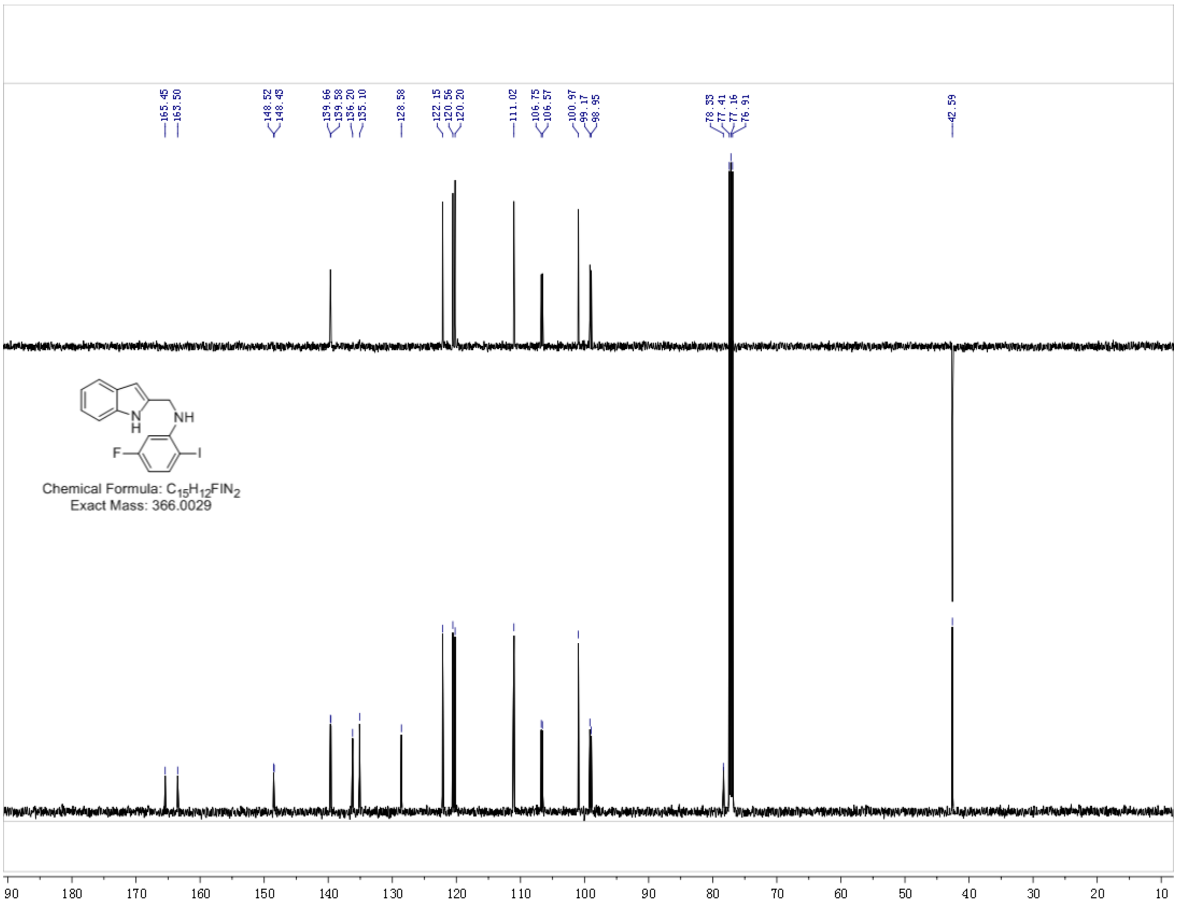

3-fluoro-5,6-dihydroindolo[1,2-*a*]quinoxaline (2k)

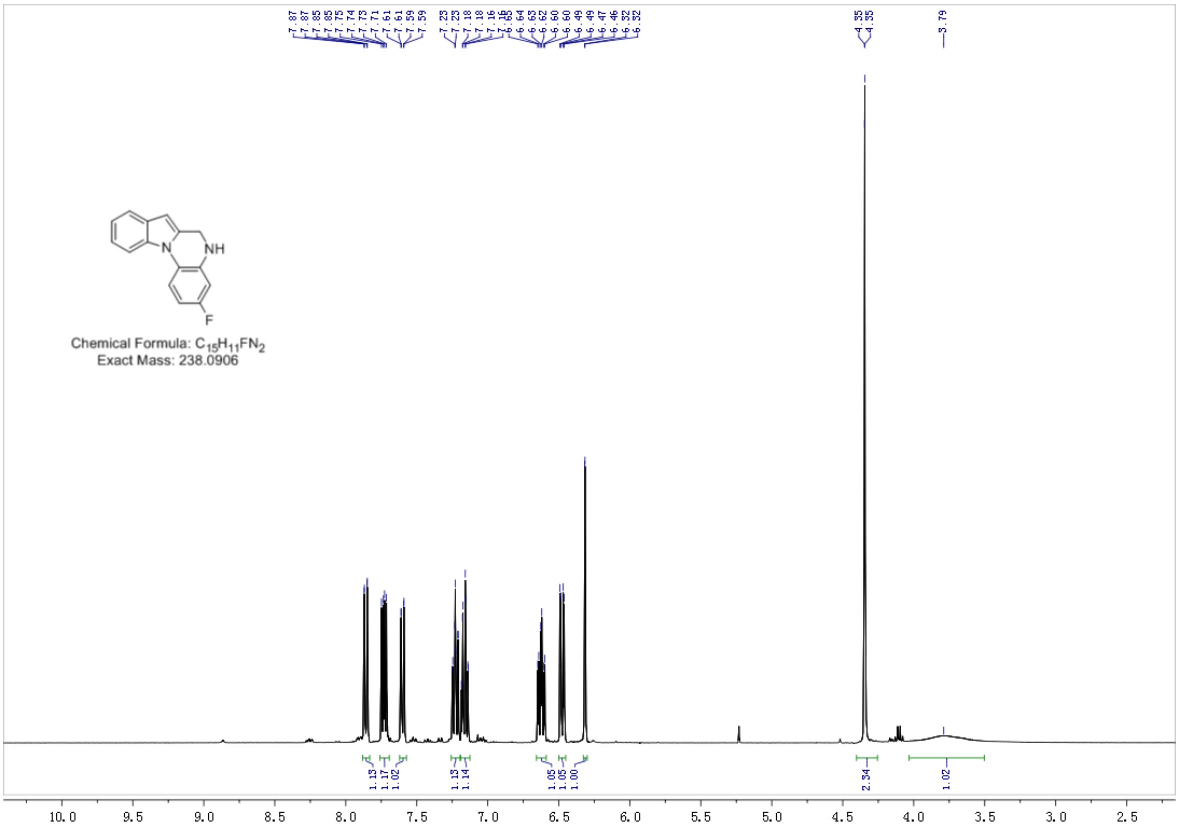

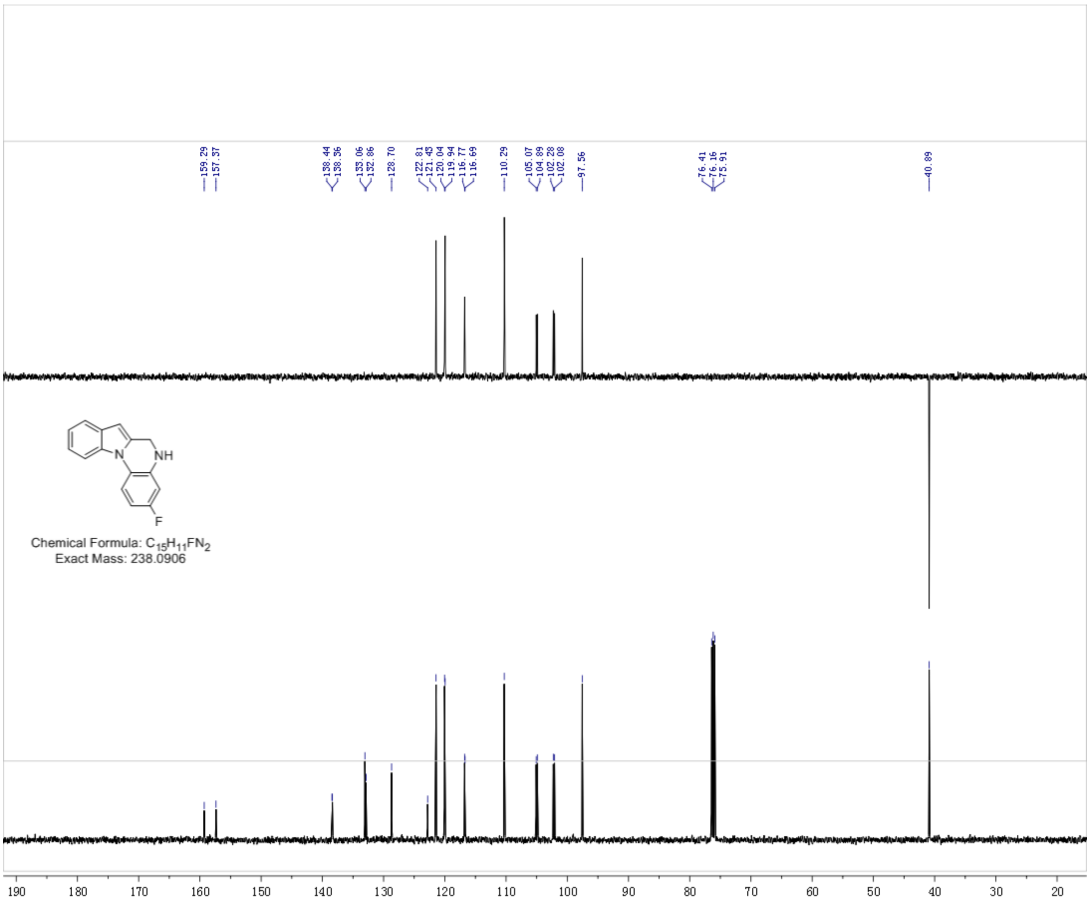

N-(1H-indol-2-ylmethyl)-4-methyl-2-iodoaniline (1l)

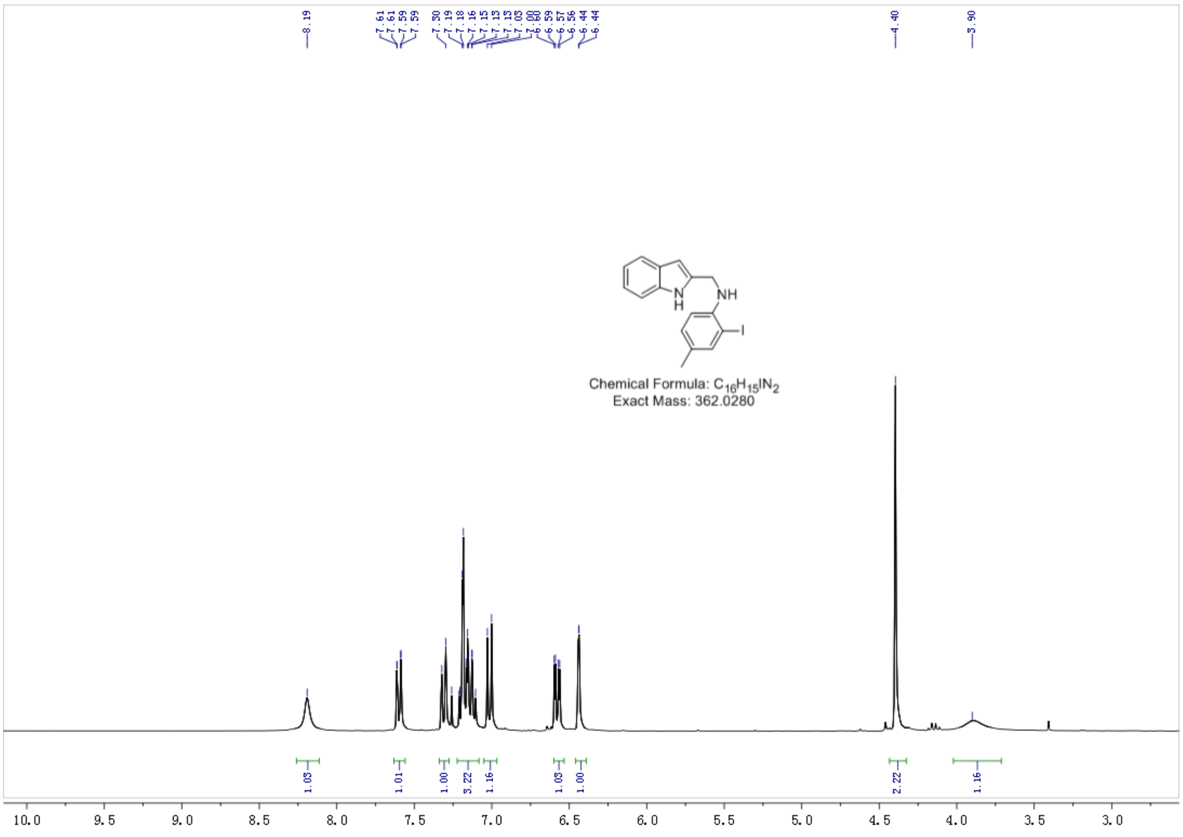

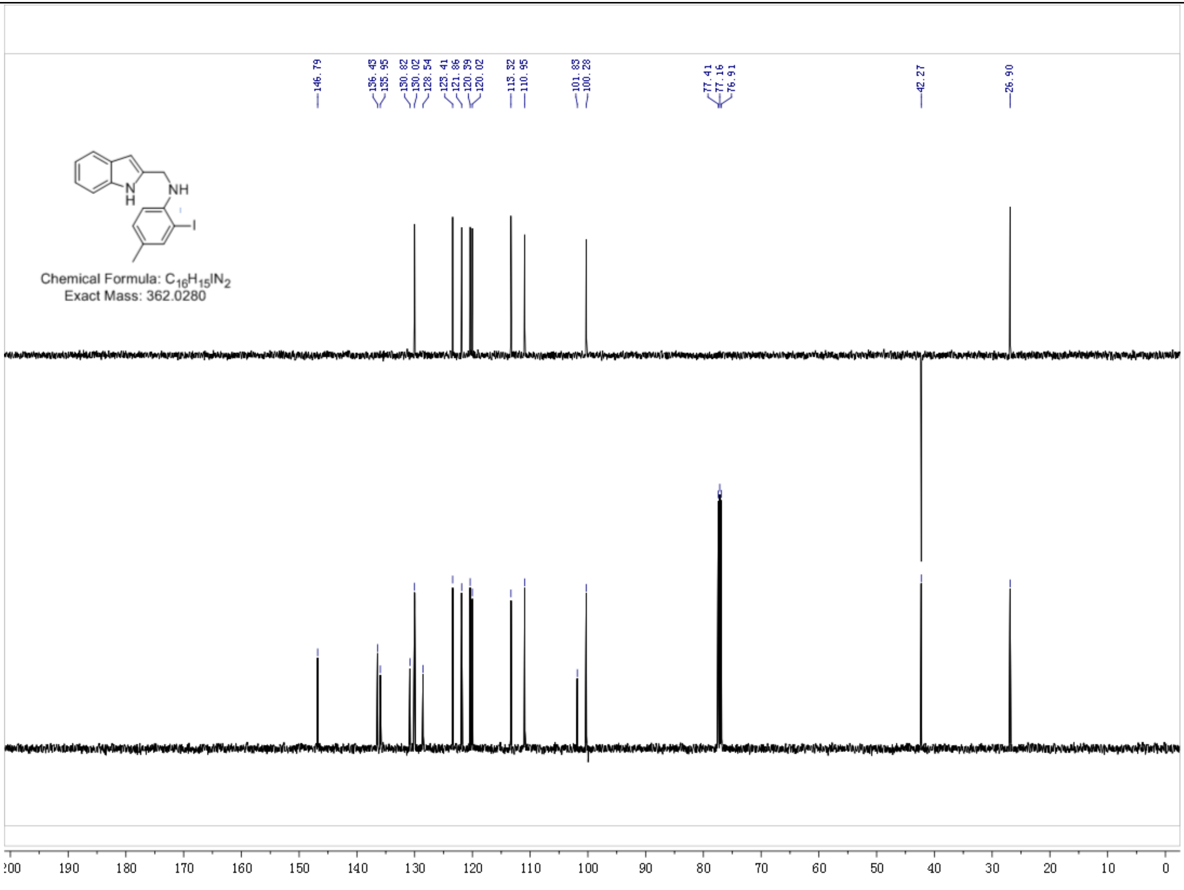

2-methyl-5,6-dihydroindolo[1,2-*a*]quinoxaline (2l)

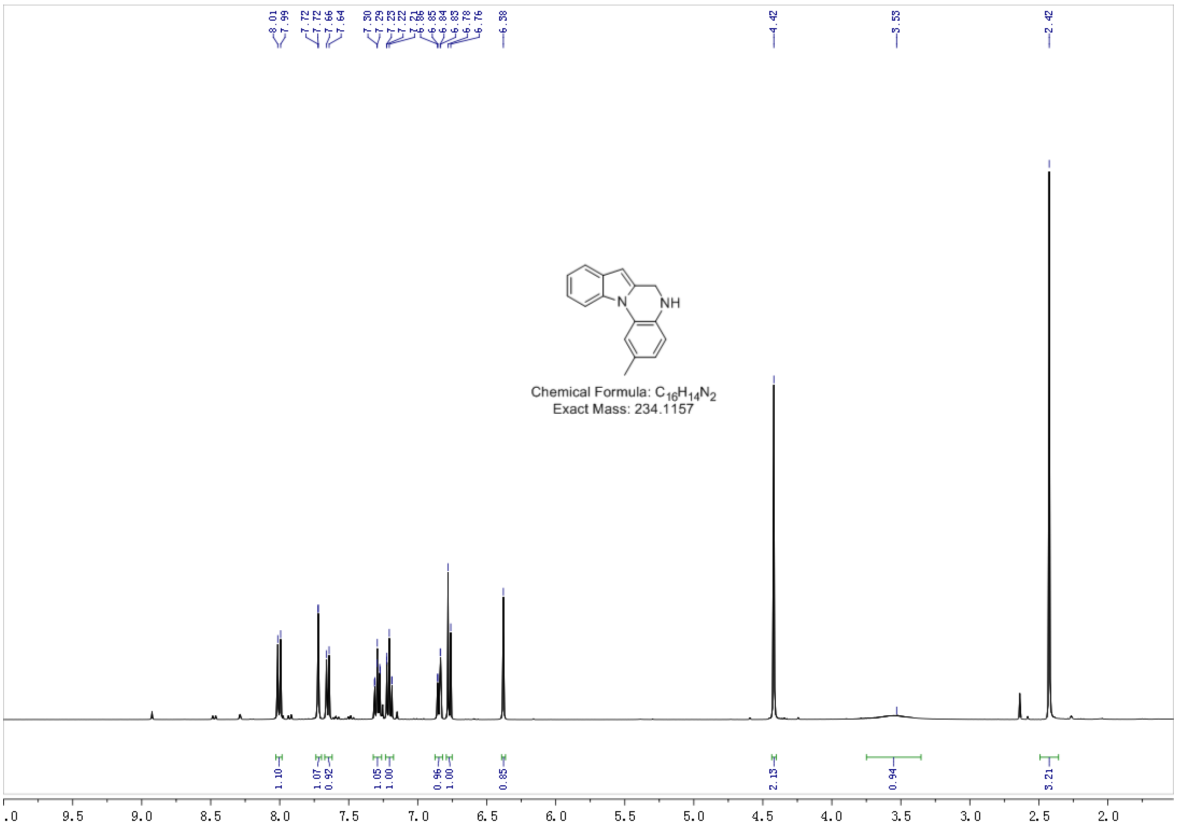

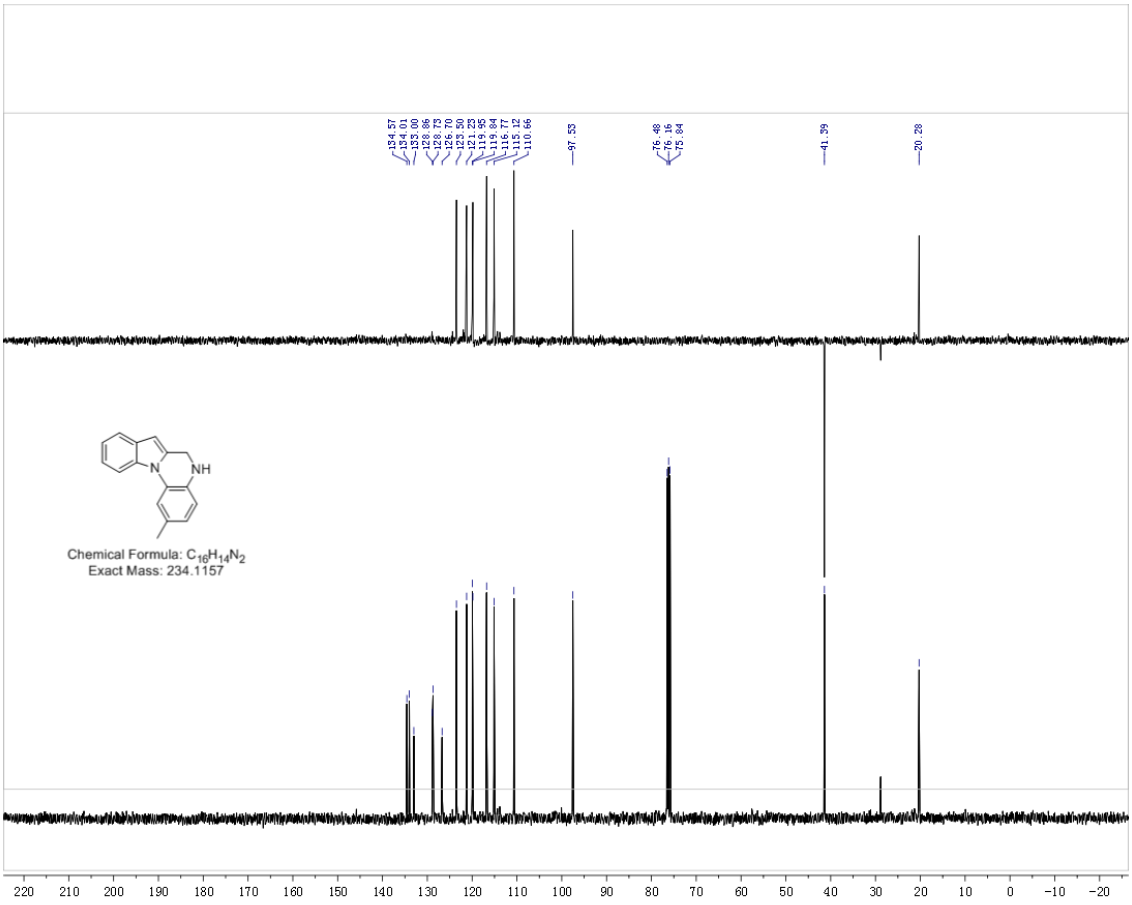

N-(1H-indol-2-ylmethyl)-2-bromoaniline (1m)

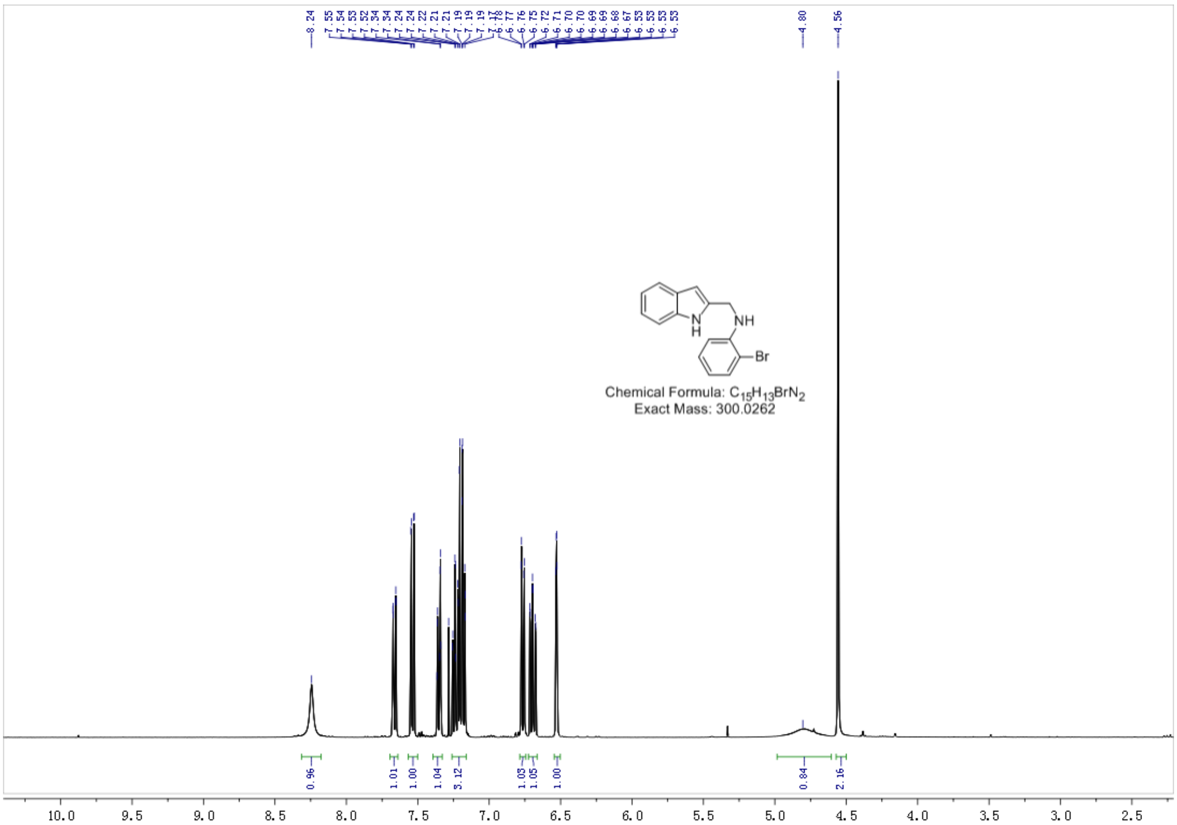

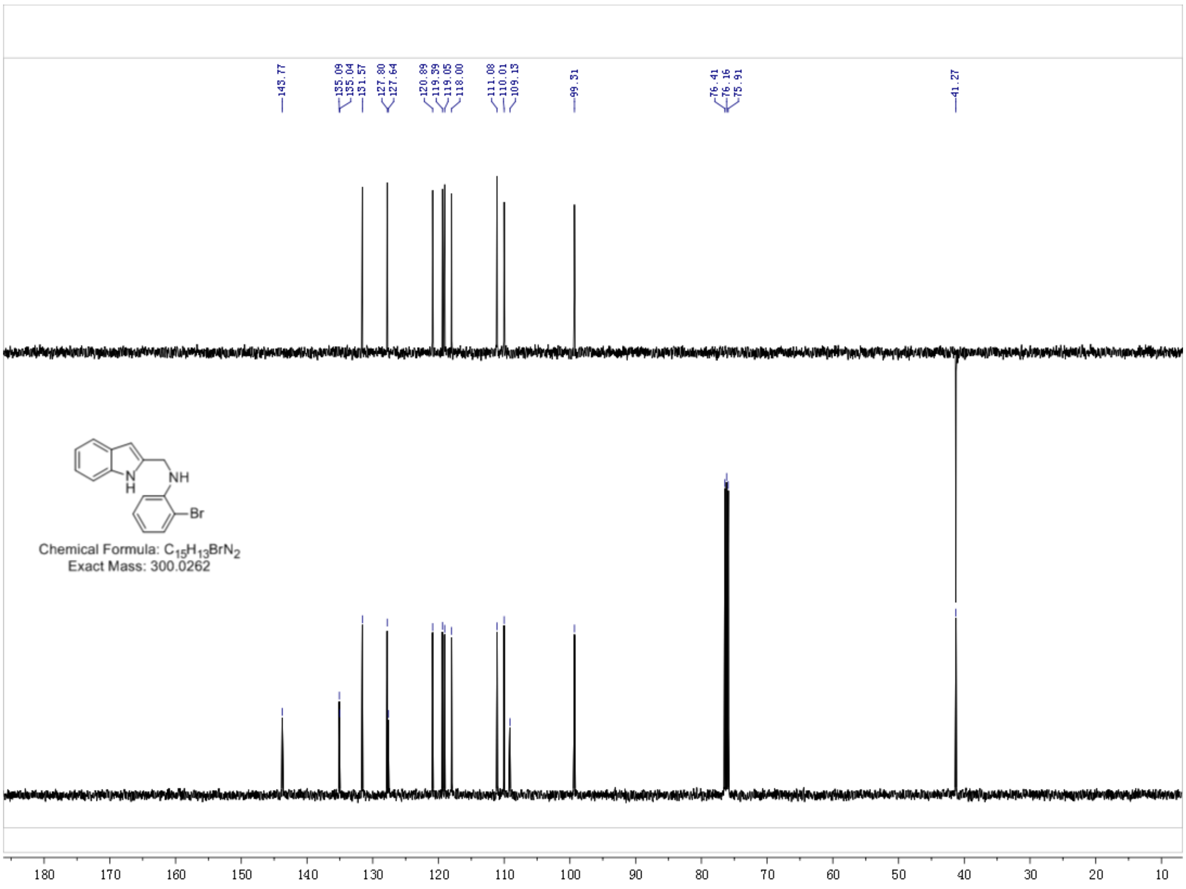

N-(1*H*-indol-2-ylmethyl)-2-bromo-4-chloroaniline (1n)

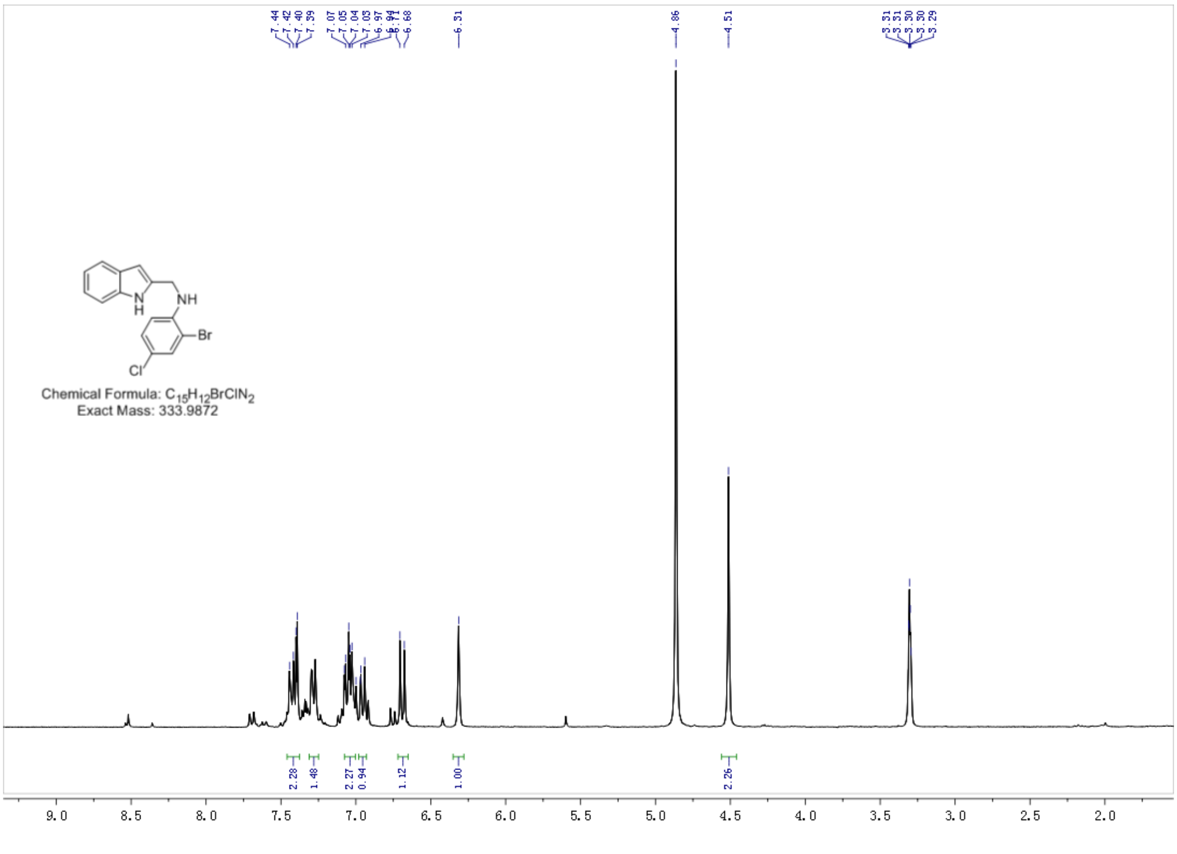

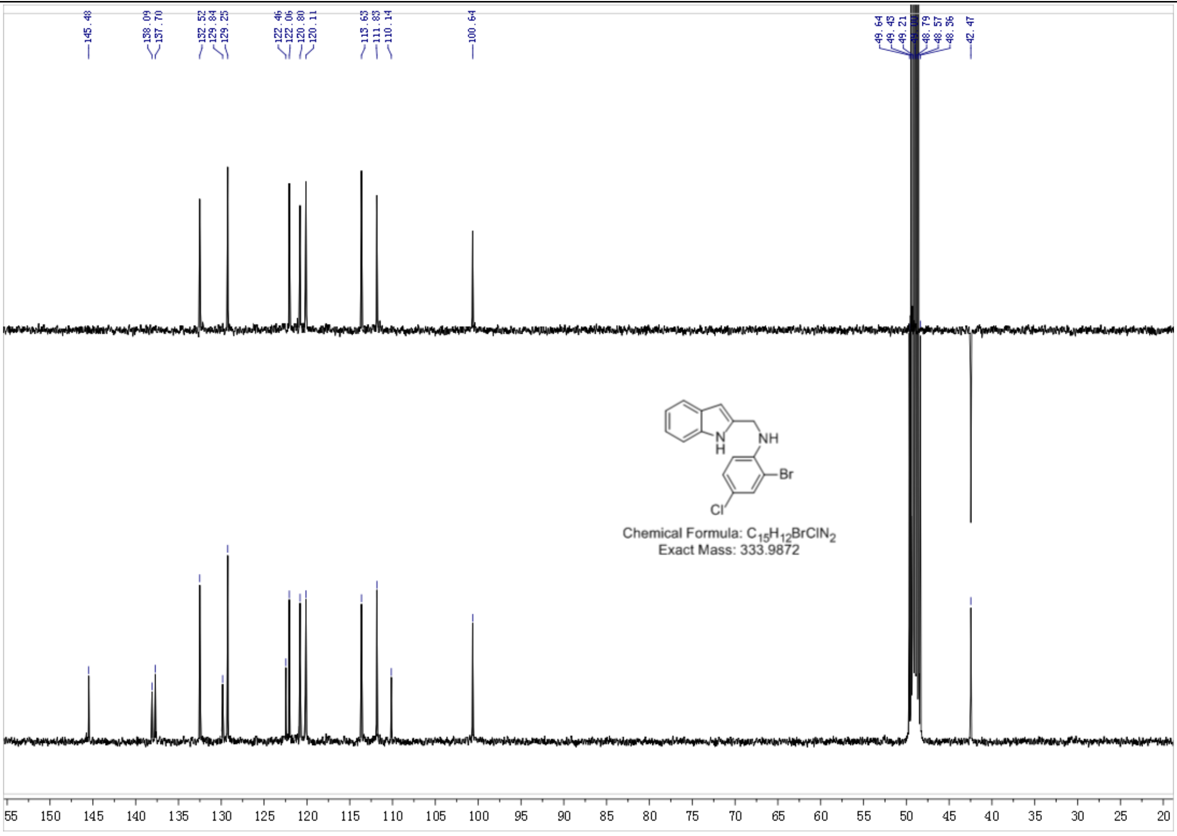

2-chloro-5,6-dihydroindolo[1,2-*a*]quinoxaline (2n)

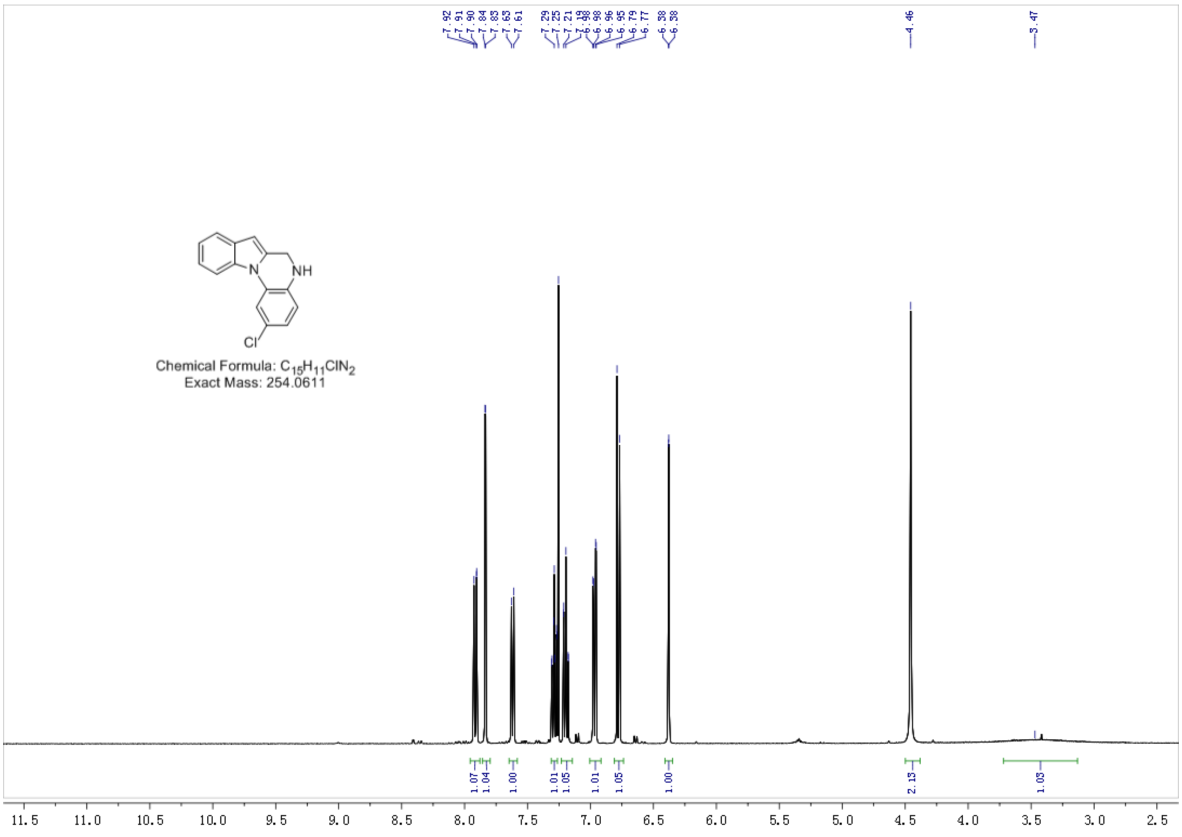

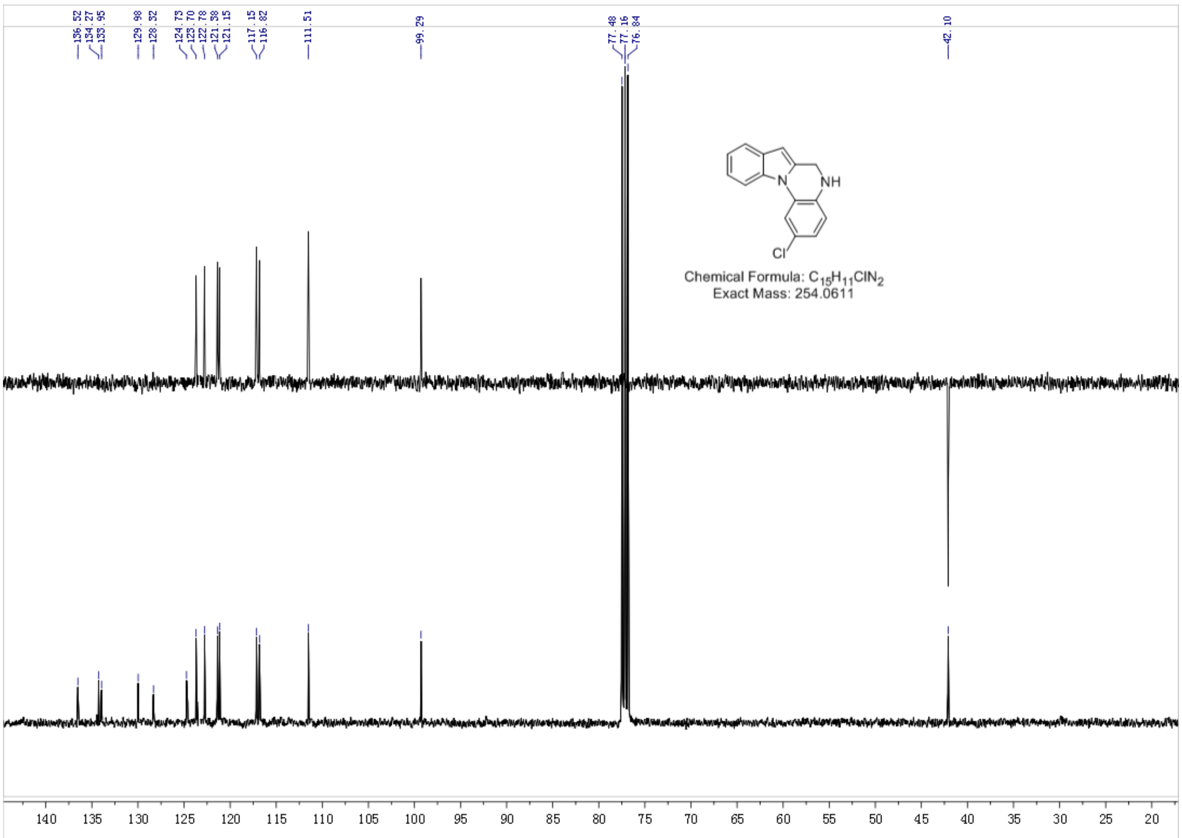

N-(1H-indol-2-ylmethyl)-2-bromo-4-methylaniline (1o)

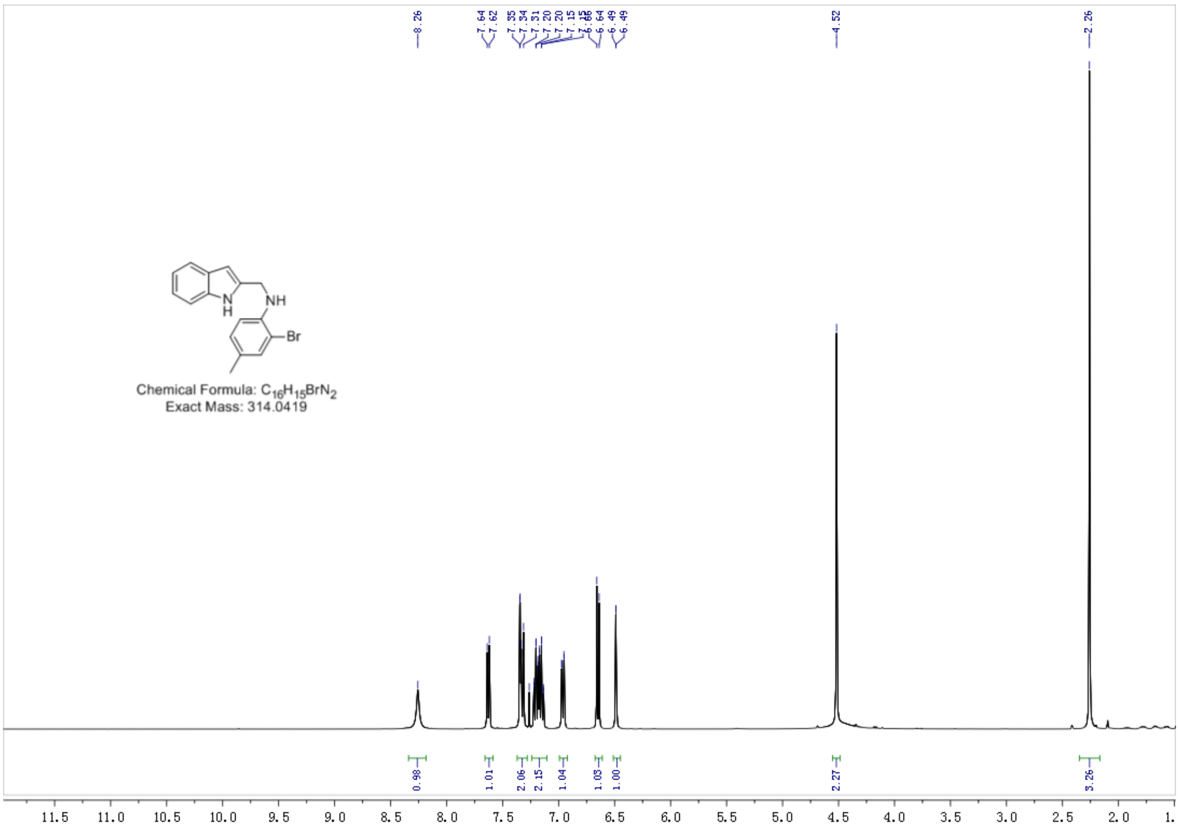

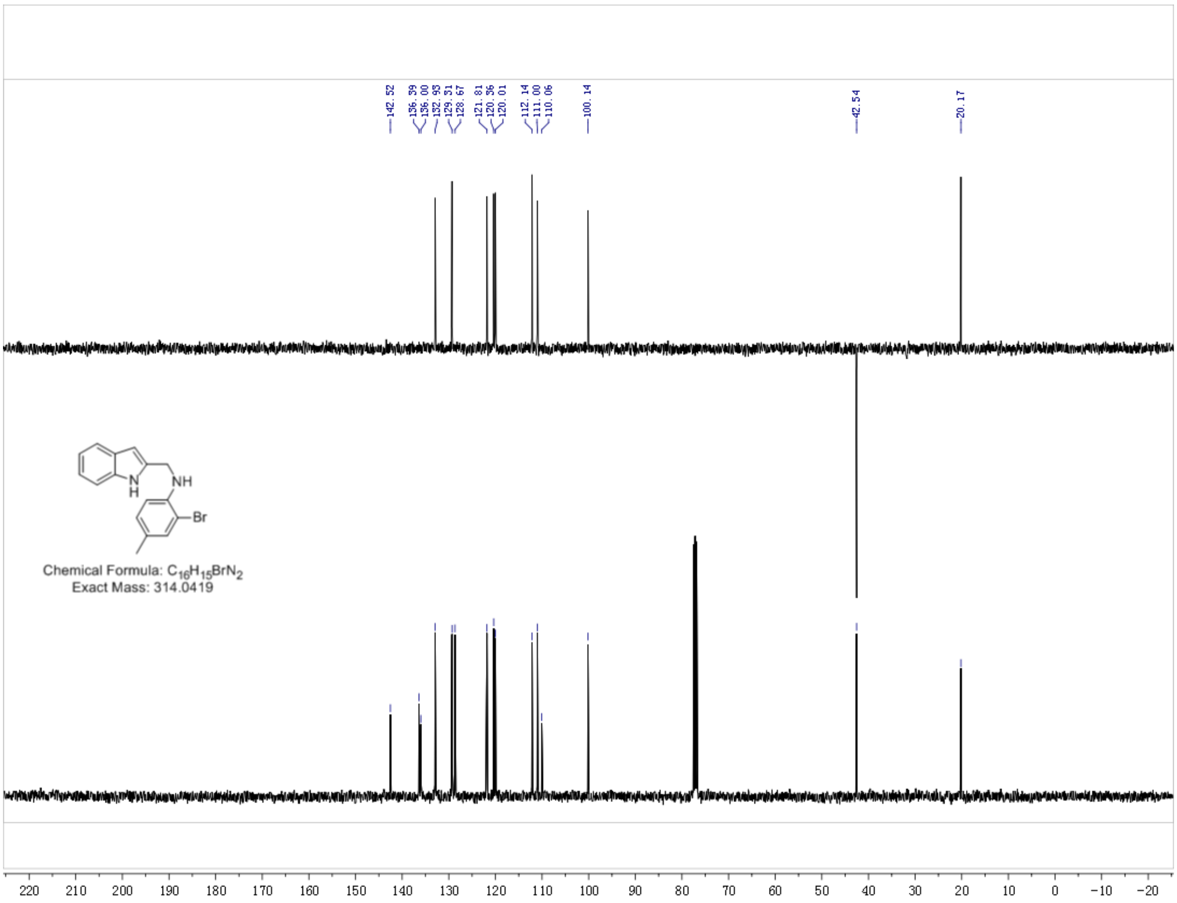

N-(1H-indol-2-ylmethyl)-2-bromo-5-fluoroaniline (1p)

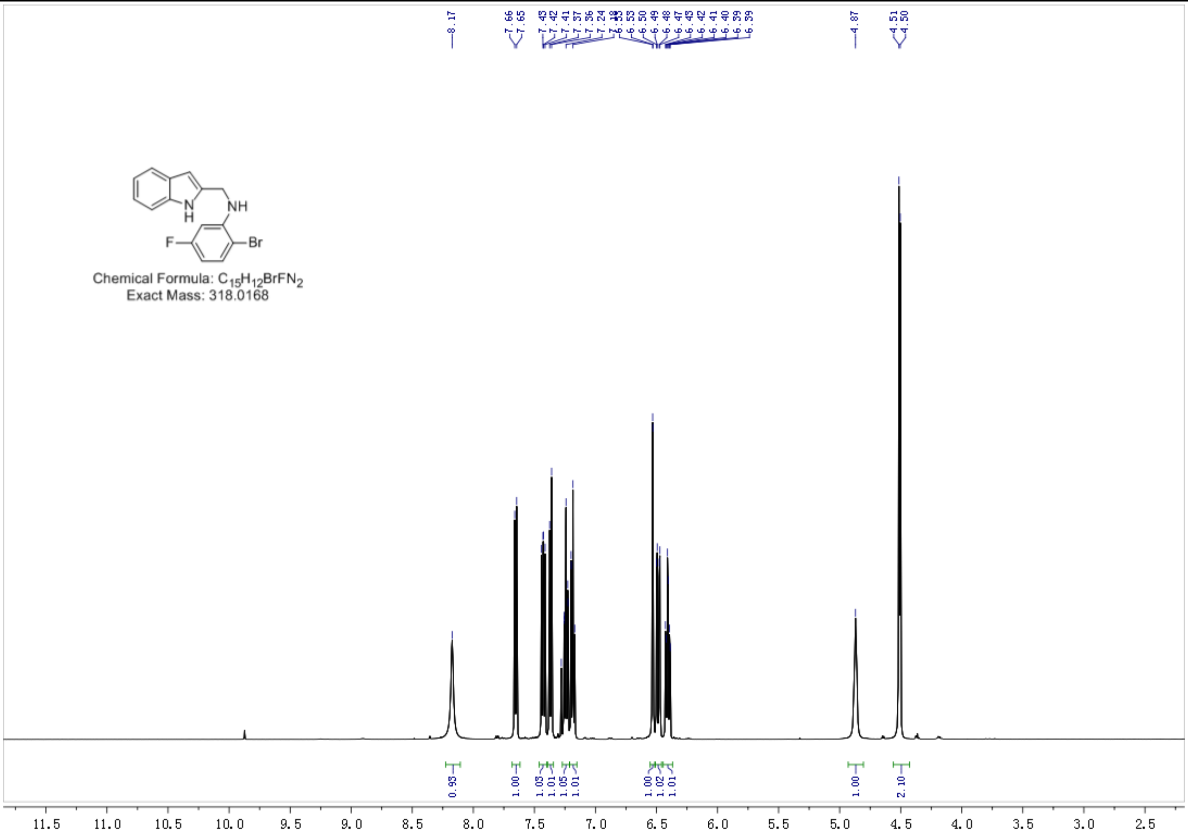

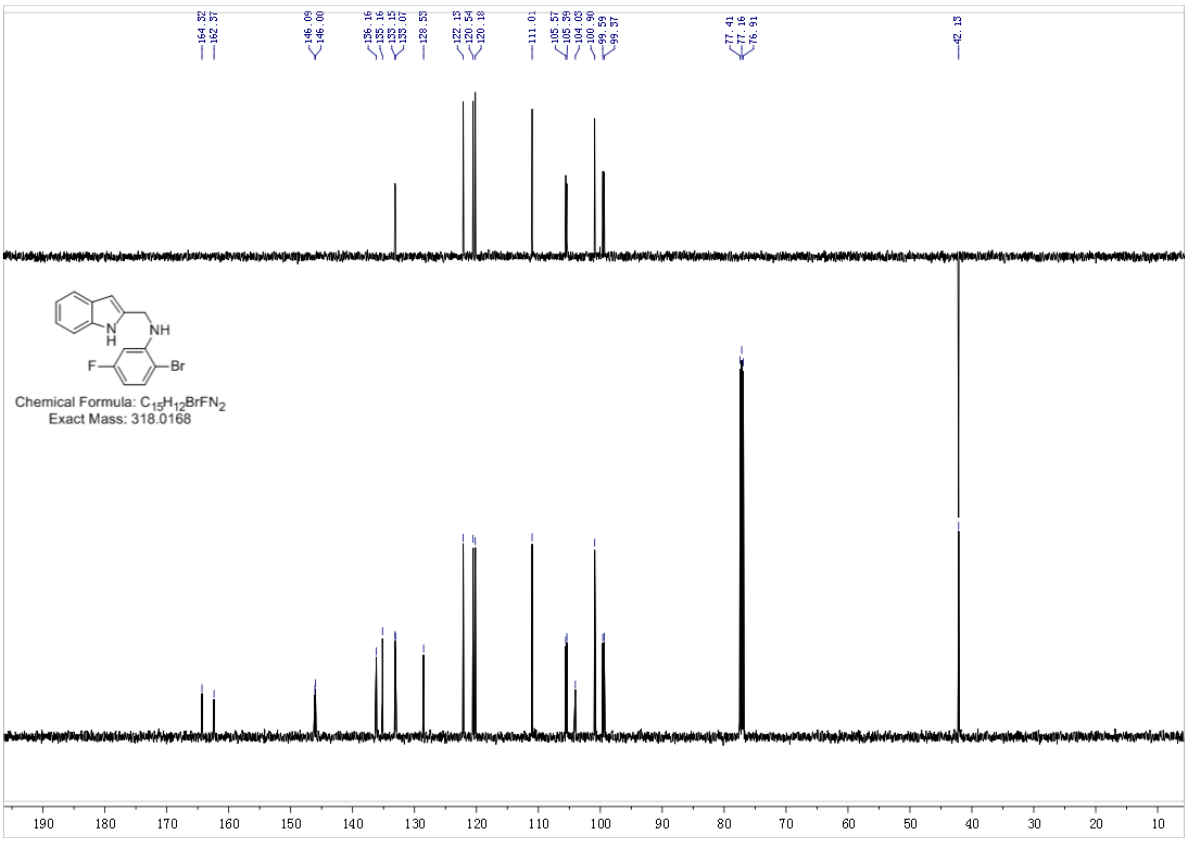

Supplement: File 1 — General information, experimental details, characterization data and copies of 1H and 13C NMR spectra. [file Beilstein_J_Org_Chem-09-2463-s001.pdf]
